# The translational chasm in machine learning for triple-negative breast cancer: a quantitative landscape assessment

**DOI:** 10.3389/fonc.2026.1912317

**Published:** 2026-09-24

**Authors:** Nadire Yiming, Yilina Saibaidoula, Kadeyanmu Abulimiti, Jianghua Ou

**Affiliations:** 1 Department of Breast Surgery, The Affiliated Tumor Hospital of Xinjiang Medical University, Urumqi, China; 2 Department of Cardiac Surgery, The First Affiliated Hospital of Xinjiang Medical University, Urumqi, China

**Keywords:** bibliometrics, deep learning, machine learning, multi-database analysis, triple-negative breast cancer

## Abstract

**Background:**

Triple-negative breast cancer (TNBC) is the most aggressive subtype of breast cancer and the one with the fewest therapeutic targets. The application of machine learning (ML) in TNBC research is becoming increasingly widespread; however, systematic bibliometric studies focusing on this rapidly evolving interdisciplinary field are still lacking.

**Methods:**

This study systematically searched the Web of Science Core Collection and Scopus databases for relevant literature on the application of ML in the field of TNBC published between January 2011 and January 2026. These publications were analyzed using VOSviewer, CiteSpace, and the R/Bibliometrix package. An independent external validation was conducted using the same search strategy in the PubMed database. Additionally, clinical trials and randomized controlled trials were identified through PubMed to quantify the clinical translation gap in this field.

**Results:**

We conducted a bibliometric analysis of 394 publications and found that global publication output exhibits a nonlinear growth trend. China ranked first in publication volume with 180 papers, while the United States ranked first in academic influence with an average of 39.09 citations per paper; the collaboration network revealed a global pattern with China and the United States as dual hubs. Co-occurrence analysis of keywords identified six major thematic clusters, broadly corresponding to three domains: disease understanding, methodological innovation, and clinical translation. Citation emergence analysis revealed that “deep learning,” “radiomics,” and “tumor-infiltrating lymphocytes” represent the most robust emerging frontiers at present. Cluster analysis of co-cited literature identified three dominant research themes: prognostic prediction models, radiomic diagnosis, and multi-omics integration. External validation results from PubMed showed high consistency with the main analysis; however, the clinical translation gap analysis revealed a 16:1 retrospective-validation-to-development ratio (24 retrospective validation studies versus 394 development studies) and a 44:1 prospective-trial-to-development ratio (9 registered trials versus 394 development studies). No prospective interventional trials were identified.

**Conclusion:**

Research on machine learning in the field of triple-negative breast cancer (TNBC) is currently undergoing rapid expansion, with current research hotspots centered on radiomics, deep learning, and the discovery of multi-omics biomarkers. However, clinical validation remains scarce: only 24 retrospective validation studies (6.1%) and 9 registered trials (2.3%) were identified relative to 394 computational development studies. Our bibliometric analysis reveals a substantial gap between computational publication output and clinical validation evidence, and the research paradigm remains primarily methodology-driven rather than clinically anchored. Multicenter, prospective clinical trials with patient-centered endpoints are needed to bridge this structural translation gap.

## 1 Introduction

As the most common malignant tumor among women worldwide, the incidence of breast cancer is becoming increasingly severe, and the resulting public health burden continues to rise. According to global cancer statistics for 2022, there were approximately 2.3 million new cases of breast cancer and approximately 666,000 deaths that year, making it one of the leading causes of cancer death among women globally (1); It is projected that by 2040, annual new cases will exceed 3 million, and annual deaths will surpass 1 million (2). Triple-negative breast cancer (TNBC) accounts for approximately 15%–20% of all breast cancers (3) and is the most aggressive subtype with the most limited treatment options. The core challenge lies in its high molecular heterogeneity and the lack of targetable therapeutic biomarkers: due to the absence of estrogen receptor, progesterone receptor, and HER2 expression, endocrine therapy and anti-HER2 targeted therapy are not applicable, leaving chemotherapy as the sole systemic treatment option for a long time (4, 5). This lack of therapeutic targets, compounded by significant molecular heterogeneity, results in highly variable treatment responses among patients, with a significantly higher risk of recurrence and mortality compared to other subtypes, creating an urgent clinical need for novel diagnostic and therapeutic strategies (6, 7).

Deep learning automatically performs feature extraction and representation learning through multi-layer neural networks, offering significant advantages in processing high-dimensional medical imaging data. Methods such as logistic regression, random forests, and support vector machines remain common tools for clinical predictive modeling. In the field of cancer in general, including breast cancer, particularly in TNBC, these methods have been deeply integrated into screening, diagnosis, treatment decision-making, and prognosis assessment (8). In recent years, artificial intelligence has established a multi-faceted presence across the entire TNBC diagnosis and treatment continuum: at the imaging diagnosis level, auxiliary diagnostic models based on mammography, ultrasound, and MRI have demonstrated potential for lesion detection and distinguishing between benign and malignant lesions (9–11); at the treatment decision-making level, predictive models integrating clinical pathology and multi-omics data are used to assess response to neoadjuvant chemotherapy and long-term recurrence risk (12, 13); at the molecular subtyping level, ML-based computational methods have been employed to identify prognostically significant subtype characteristics and biomarkers from genomic and transcriptomic data (14).

Although the number of papers on machine learning (ML) in the field of triple-negative breast cancer (TNBC) is growing rapidly, bibliometric studies specifically focused on this interdisciplinary field remain scarce. Existing bibliometric analyses of breast cancer and artificial intelligence have adopted one of two approaches: either they examined AI in breast cancer as a whole without subtype stratification, making it impossible to capture the unique molecular heterogeneity and therapeutic challenges specific to TNBC; or they focused on specific technical areas or clinical stages within TNBC, such as biomarker discovery or neoadjuvant therapy without treating machine learning as a cross-disciplinary methodological object of study. Consequently, three critical questions remain unanswered: which countries and institutions are leading this interdisciplinary direction; how the knowledge structure is evolving across methodological innovation and clinical translation; and whether the depth of clinical validation matches the scale of computational output. Most importantly, no prior bibliometric work has quantified the gap between algorithm development and clinical implementation in this field.

To address these gaps, this study conducts a comprehensive bibliometric analysis of 394 core publications on ML and TNBC from 2011 to 2026, with three specific contributions. First, by focusing exclusively on the TNBC subtype and using ML as the central methodological lens, this study bridges the gap between macro-level bibliometric analyses of breast cancer AI and specialized TNBC analyses focused on single technical areas. Second, it employs an analytical strategy combining merged database coverage (Web of Science and Scopus) with independent PubMed validation to ensure robustness. Third, and most distinctively, it quantifies the clinical translation gap for the first time through a stratified three-tier retrieval strategy that distinguishes retrospective validation studies from registered prospective trials, revealing a 16:1 retrospective-validation-to-development ratio and a 44:1 prospective-trial-to-development ratio. These features enable the study to map not only the global landscape and knowledge structure of ML-TNBC research but also the structural disconnect between algorithmic proliferation and clinical implementation, thereby providing quantifiable, evidence-based grounds for shifting the field from methodological exploration toward patient-centered validation.

## 2 Methods

### 2.1 Data retrieval and preprocessing

The literature search was conducted in the Web of Science Core Collection (WoSCC) and Scopus databases. WoSCC and Scopus were selected as primary databases because they provide: (a) comprehensive multidisciplinary coverage spanning biomedical and computational sciences, essential for capturing the interdisciplinary nature of ML-oncology research; (b) structured citation metadata and reference lists required for co-citation and bibliographic coupling analysis; and (c) peer-reviewed indexing standards that reduce the risk of including non-peer-reviewed publications. Embase was excluded because it focuses primarily on biomedical and pharmaceutical literature with limited coverage of computer science and engineering journals where AI methodology papers are frequently published. IEEE Xplore was excluded because it indexes predominantly engineering conference proceedings and technical standards rather than clinical oncology journals, making it unsuitable as a primary source for capturing the clinical validation component of this field. PubMed was not selected as a primary database because it lacks the structured citation network data required for bibliometric analysis, although it was used as a supplementary source for external validation (Section 2.3) and clinical trial identification (Section 2.4).

The WoSCC search parameters were set as follows: TS=((“triple negative breast cancer” OR “triple-negative breast cancer” OR “TNBC”) AND (“machine learning” OR “deep learning” OR “artificial intelligence” OR “neural network” OR “support vector machine” OR “random forest” OR “convolutional neural network” OR “CNN” OR “natural language processing” OR “NLP” OR “transfer learning” OR “ensemble learning” OR “gradient boosting” OR “XGBoost” OR “supervised learning” OR “unsupervised learning”)). The Scopus search query was: TITLE-ABS-KEY((“triple negative breast cancer” OR “triple-negative breast cancer” OR “TNBC”) AND (“machine learning” OR “deep learning” OR “artificial intelligence” OR “neural network” OR “support vector machine” OR “random forest” OR “convolutional neural network” OR “CNN” OR “natural language processing” OR “NLP” OR “transfer learning” OR “ensemble learning” OR “XGBoost”)). Only articles and reviews were included. The WoSCC search was conducted on February 19, 2026, with data exported on February 19, 2026. The Scopus search was conducted on February 21, 2026, with data exported on February 21, 2026. Both databases were accessed via institutional subscription platforms (Clarivate Web of Science platform for WoSCC, Elsevier Scopus platform for Scopus). To avoid bias from daily database updates, the primary search was completed within a 48-hour window.

The literature screening followed a structured process consistent with the PRISMA Extension for Scoping Reviews (PRISMA-ScR). Initial database retrieval yielded 1,614 raw records (WoSCC: n=491; Scopus: n=1,123). Database-level filtering by language (English), time range (January 1, 2011 to January 31, 2026), and document type (article, review article) excluded 217 records (WoSCC: n=44; Scopus: n=173), leaving 1,397 records. The screening flow presented in **Figure 1** begins at this stage. Two independent reviewers then conducted title and abstract screening against predefined inclusion criteria: (a) relevance to both machine learning and triple-negative breast cancer; (b) article or review publication type; (c) English language; (d) publication within the specified time range. A total of 655 records were excluded at this stage (WoSCC: n=54; Scopus: n=601), primarily for lack of relevance to the ML-TNBC intersection, including pan-cancer studies not specific to TNBC. The remaining 742 records (WoSCC: n=393; Scopus: n=349) were imported into Python and deduplicated using BibexPy (15), which identified and merged 348 duplicate entries. All 394 unique records underwent full-text assessment and were confirmed to meet the inclusion criteria; no additional exclusions were required because the criteria were fully assessable at the title and abstract level. During the screening process, two independent reviewers cross-checked the inclusion criteria, and any discrepancies were resolved through discussion or consultation. The complete screening flow is presented in **Figure 1**.

**Figure 1:**
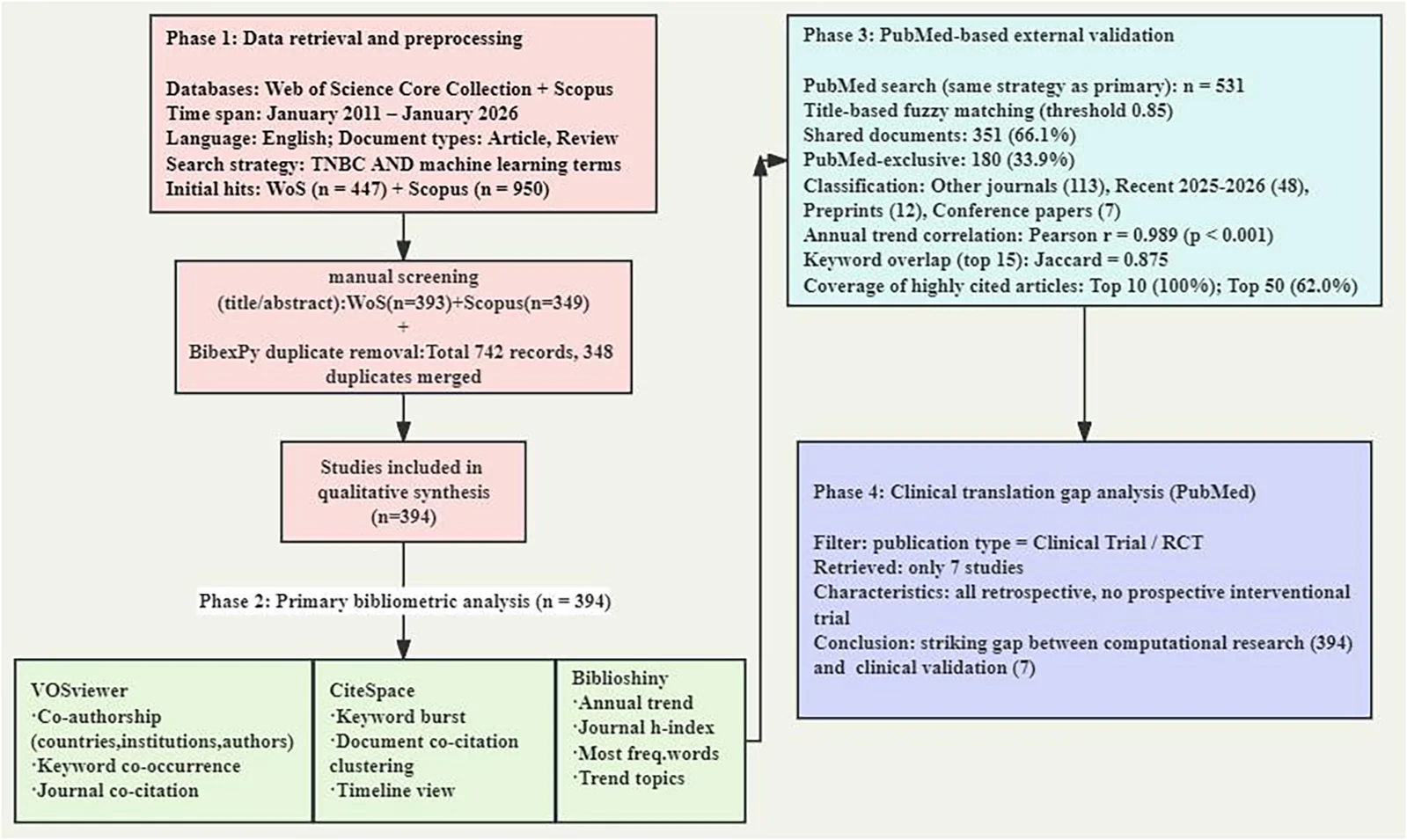
Flowchart.

### 2.2 Data analysis and visualization

This study employed VOSviewer (v1.6.20), CiteSpace (v6.4.R1), the R language (vR4.5.2), and its Bibliometrix package (v4.3.5, with the graphical interface Biblioshiny) to conduct bibliometric analysis and visualization of the 394 included articles. Each of these three tools has its own strengths and complements the others: VOSviewer excels at constructing large-scale co-occurrence and collaboration networks; CiteSpace focuses on citation network analysis and the detection of emerging research frontiers; and R/Bibliometrix provides rich statistical descriptions and topic evolution capabilities.

CiteSpace is a Java-based visual analysis tool developed by Professor Chen Chaomei, which excels at identifying the evolution and emerging trends in research fields through co-citation networks and keyword emergence detection (16). This study utilizes CiteSpace to perform co-citation clustering analysis, keyword emergence detection, and timeline visualization. The system parameters are set as follows: the time slice spans from January 2011 to January 2026, with each slice covering a one-year period; the node selection criterion uses the g-index (k=25), selecting the top 50 most frequently cited nodes for each time slice; network pruning employed the Pathfinder algorithm; cluster label extraction utilized the Log-Likelihood Ratio (LLR) algorithm; and keyword emergence detection employed the Kleinberg algorithm (γ=0.5) to identify keywords, references, or authors exhibiting abnormal surges in citation frequency (17).

VOSviewer is a computer program developed by VanEck and Waltman at Leiden University in the Netherlands that is particularly well-suited for constructing and visualizing large-scale bibliometric networks (18). This study utilized VOSviewer to construct networks of country/institutional co-authorship, author collaboration, keyword co-occurrence, and journal co-citation. Specific thresholds (such as minimum occurrence frequency and minimum citation count) and network pruning details varied depending on the object of analysis; relevant parameters are detailed in the captions or main text accompanying each network diagram in the Results section. In the network diagrams generated by VOSviewer, node size represents the weight of the item (e.g., number of publications or citation frequency), node color indicates cluster affiliation, and line thickness indicates the strength of co-occurrence, collaboration, or co-citation.

Biblioshiny is an open-source scientometrics tool based on the R language, developed by Aria and Cuccurullo, which provides an integrated analysis workflow ranging from data import and descriptive statistics to scientific visualization (19). In this study, the Rggplot2 package was used to directly count the annual number of publications. The LOESS method was employed for smoothing and fitting the 2011–2025 annual trend (**Figure 2**). The smoothing parameter (span) was selected through systematic sensitivity analysis: five candidate values (0.30, 0.35, 0.40, 0.45, 0.50) were evaluated against the bias–variance tradeoff. Span values below 0.4 produced near-zero residuals (ResidualSD ≈ 0 at span = 0.30) and R² approaching 1.0, indicating dangerous overfitting; span = 0.50 exhibited excessive smoothing with attenuated R² (0.987) and flattened the rapid-expansion trajectory. Span = 0.40 achieved the optimal balance (ResidualSD = 12.38, R² = 0.991), corresponding to a ~3-year smoothing window (6.0 equivalent parameters for n = 15) that aligns with the observed three-phase growth pattern without tracking annual stochasticity. The fitted curve presents a 95% confidence envelope (± 2 SE; **Figure 2**). Sensitivity analysis confirmed robustness: predicted 2025 publication counts varied by less than 2.1% across the full span range, and the S-shaped growth direction was preserved in all scenarios (**Supplementary Table 8**, **Supplementary Figure 1**). Pearson’s correlation coefficient was calculated to quantify the linear trend component (r = 0.845, 95% CI: 0.587–0.947, p < 0.001), and Spearman’s rank correlation (ρ = 0.929, p < 0.001) was used as a non-parametric sensitivity check to confirm robustness under non-linearity assumptions. Biblioshiny was also used to calculate the h-index and g-index, extract high-frequency terms, and perform descriptive statistics. High-frequency terms were extracted using the unigrams mode from the title and abstract fields. After removing English stop words, synonyms were merged (e.g., “triple-negative breast cancer” was standardized to “TNBC,” and “machine learning” to “ML”).

**Figure 2:**
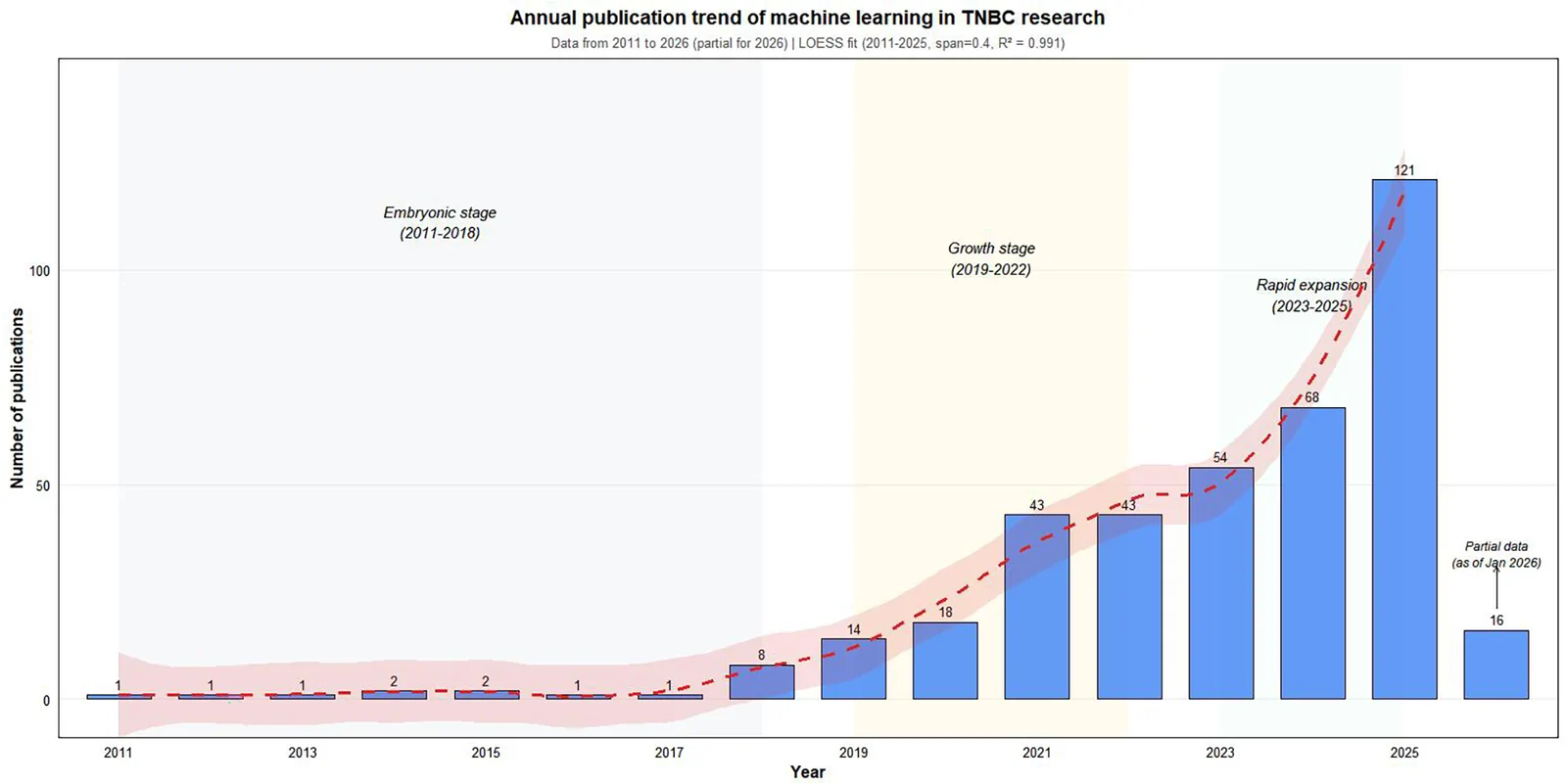
Annual publication trend of machine learning in triple-negative breast cancer research. Bars represent observed publication counts from Web of Science and Scopus (2011–2026; 2026 is partial-year data). The red dashed line shows the LOESS fit (span = 0.4, degree = 2) to 2011–2025 data (R² = 0.991), with the shaded red band indicating the 95% confidence envelope (± 2 SE). Key predicted values: 2020 = 23.3 (95% CI: 16.0–30.7), 2023 = 50.6 (95% CI: 43.2–58.0), 2025 = 118.5 (95% CI: 108.6–128.5). Background shading demarcates three growth phases: embryonic (2011–2018, gray), growth (2019–2022, yellow), and rapid expansion (2023–2025, green).

### 2.3 PubMed-based external representativeness validation

To assess the representativeness of the primary analysis dataset (WoSCC+Scopus) and to determine whether the conclusions were influenced by database selection, we conducted an independent search in PubMed. The search terms were consistent with those used in the main analysis:((“Triple Negative Breast Neoplasms”[Mesh] OR “triple negative breast cancer”[tiab] OR “triple-negative breast cancer”[tiab] OR “TNBC”[tiab]) AND (“Machine Learning”[Mesh] OR “Deep Learning”[Mesh] OR “Neural Networks, Computer”[Mesh] OR “Artificial Intelligence”[Mesh] OR “Support Vector Machine”[Mesh] OR “machine learning”[tiab] OR “deep learning”[tiab] OR “artificial intelligence”[tiab] OR “neural network*”[tiab] OR “CNN”[tiab] OR “random forest”[tiab] OR “XGBoost”[tiab] OR “natural language processing”[tiab])) AND (journal article[pt] OR review[pt]) AND 2011/01/01:2026/01/31[pdat] AND english[lang], and the search was restricted to journal articles or reviews (journal article[pt] OR review[pt]), with a time range from January 1, 2011, to January 31, 2026, and language set to English. A total of 531 articles were identified and used as the validation set. We calculated the overlap rate between the PubMed validation set and the main dataset using title-based fuzzy string matching (Python’s difflib library, with a similarity threshold of 0.85). For articles unique to PubMed, we classified them based on journal title, publication year, and whether they were preprints or conference papers. Regarding keyword comparison, we extracted high-frequency keywords from the titles and abstracts. The preprocessing steps include: (1) preserving compound words (e.g., treating “machine learning” as a single unit without splitting it); (2) removing NLTK stopwords and custom academic noise words (e.g., “study,” “results,” etc.); (3) not performing any synonym merging (e.g., not unifying “TNBC” with “triple-negative breast cancer,” nor standardizing “breast cancer”) to preserve the original differences between databases and provide conservative consistency estimates. The top 15 high-frequency keywords from each dataset were extracted, and the Jaccard similarity coefficient and Spearman’s rank correlation coefficient were calculated. Publication volume trends were visualized using R to plot annual publication volume overlay charts, and Pearson’s correlation coefficient was calculated to assess trend consistency. Highly Cited Paper Coverage: This was determined by verifying, one by one, whether the top 10 highly cited papers from the main analysis were included in PubMed. Additionally, the 50 most frequently cited papers were extracted from PubMed to verify their inclusion rate in the main dataset. These validations aim to comprehensively assess the robustness of the main analysis results and ensure that conclusions are not influenced by bias from a single database source. To assess matching accuracy, all 31 fuzzy-matched pairs were manually verified against title concordance, publication year, and first-author identity (estimated false-positive rate = 0%). Ten randomly sampled unmatched articles were manually searched using full titles, DOIs, and author-year combinations to estimate false-negative rate (≈ 0%).

### 2.4 Clinical translation gap analysis

To assess the clinical translation status of machine learning in triple-negative breast cancer, we employed a tiered retrieval strategy.

Tier 1. PubMed was searched using the same terms as the main analysis with publication type restricted to “Clinical Trial” or “Randomized Controlled Trial” (time range: January 1, 2011 to January 31, 2026; language: English). The search string was:((triple-negative breast cancer[Title/Abstract]) OR (TNBC[Title/Abstract])) AND ((machine learning[Title/Abstract]) OR (artificial intelligence[Title/Abstract]) OR (deep learning[Title/Abstract])) AND ((Clinical Trial[Publication Type]) OR (Randomized Controlled Trial[Publication Type]));

However, relying solely on PubMed publication-type filters might miss clinically relevant studies lacking explicit trial indexing. Therefore, Tier 2 used an expanded PubMed search without publication-type restriction. The search string was:((triple-negative breast cancer[Title/Abstract]) OR (TNBC[Title/Abstract])) AND ((machine learning[Title/Abstract]) OR (artificial intelligence[Title/Abstract]) OR (deep learning[Title/Abstract])) AND ((prospective[Title/Abstract]) OR (multicenter[Title/Abstract]) OR (external validation[Title/Abstract]) OR (diagnostic accuracy[Title/Abstract]) OR (clinical cohort[Title/Abstract]) OR (radiomics[Title/Abstract]) OR (digital pathology[Title/Abstract]) OR (neoadjuvant response[Title/Abstract]) OR (immunotherapy stratification[Title/Abstract]));

A descriptive analysis was conducted covering first author, publication year, journal, sample size, machine learning application scenario, key performance metrics (e.g., AUC), study design type (e.g., retrospective cohort or diagnostic accuracy study), and multicenter versus single-center status. Articles retrieved from Tier 1 and Tier 2 were deduplicated by title and DOI, and the combined results were compiled in **Supplementary Table 5**.

Tier 3. To identify ongoing or registered prospective trials not yet published in the peer-reviewed literature, ClinicalTrials.gov and the WHO International Clinical Trials Registry Platform (ICTRP) were searched. ClinicalTrials.gov was searched in two rounds using the following field combinations: Condition: “triple-negative breast cancer” OR “TNBC”; Intervention: “machine learning” OR “artificial intelligence” OR “deep learning”/Condition: “triple-negative breast cancer” OR “TNBC”; Intervention: “radiomics”; The WHO ICTRP global search was conducted using: “triple-negative breast cancer” AND (“machine learning” OR “artificial intelligence” OR “deep learning” OR “radiomics”); Records from ClinicalTrials.gov and WHO ICTRP were deduplicated by NCT or ChiCTR identifiers, and the combined results were compiled in **Supplementary Table 6**.

By comparing the clinical validation studies from all tiers with the 394 computational studies in the main analysis dataset, we stratified the translation gap from algorithm development to clinical validation into retrospective validation and prospective trial stages.

## 3 Results

### 3.1 Publication trends

As shown in **Figure 2**, this study included a total of 394 academic articles on the application of machine learning in the field of triple-negative breast cancer (2011–2026). The global number of published papers has shown an overall upward trend. Between 2011 and 2018, the annual publication rate was extremely low (less than 2 papers per year on average), with a cumulative total of only 17 papers, accounting for 4.3% of the total publications, indicating that the application of machine learning in the TNBC field was still in its infancy. From 2019 to 2022, the field entered a period of rapid growth, with the annual number of publications surging from 8 to 43, for a cumulative total of 118. In 2023, the number of publications exceeded 50 (54), marking the onset of an exponential growth phase; by 2025, this figure reached 121, approximately 15 times the 2018 level (8). Over the past three years (2023–2025), the number of publications was 243, accounting for 61.7% of the total. We used LOESS (locally weighted regression) to perform nonparametric smoothing on the publication volume from 2011 to 2025 (span = 0.4), with a coefficient of determination (R²) of 0.991 between the fitted curve and the observed values. The fitted curve is presented with a 95% confidence envelope (± 2 SE; **Figure 2**, red shaded band).Pearson correlation confirmed a significant linear upward trend (r = 0.845, 95% CI: 0.587–0.947, p < 0.001), and Spearman’s rank correlation as a nonparametric sensitivity check yielded an even stronger association (ρ = 0.929, p < 0.001).This indicates a highly fitted nonlinear growth relationship between the temporal trend and annual publication volume, suggesting that machine learning research in the field of triple-negative breast cancer has entered a phase of rapid expansion, and this trend continues to advance.

### 3.2 Country and institutional analysis

#### 3.2.1 Countries

We analyzed the number of publications, citation frequency, and average citations per paper in the interdisciplinary field of artificial intelligence and triple-negative breast cancer across various countries. **Table 1** lists the top 10 countries by number of publications. China had the highest number of publications (180), with a total of 3,429 citations and an average of 19.05 citations per paper; the United States published 116 papers, with a total of 4,534 citations and an average of 39.09 citations per paper, indicating that individual U.S. studies have a greater impact. India ranked third (35 papers), with an average of 17.91 citations per paper. Among European countries, France had the highest average citations per paper (47.07), while Germany (32.35), Sweden (31.82), and the United Kingdom (22.33) also performed notably well. Overall, China and the United States are the leading contributors to research in this field, but the United States holds a greater advantage in terms of impact metrics.

**Table 1:** Top 10 countries with most publication.

Rank	Country	Documents	Citations	Average citations	Total link strength
1	China	180	3429	19.05	21860
2	USA	116	4534	39.09	23004
3	India	35	627	17.91	9028
4	South Korea	19	155	8.16	3798
5	England	18	402	22.33	7710
6	Germany	17	550	32.35	5181
7	France	15	706	47.07	4735
8	Saudi Arabia	12	375	31.25	4630
9	Australia	11	374	34	5596
10	Sweden	11	350	31.82	5548

At the same time, we used VOSviewer to conduct an analysis of co-authorship among countries, setting a threshold of at least two published papers per country. A total of 31 countries met the criteria and were included in the network (**Figure 3**). Node size represents the number of publications, and line thickness indicates the intensity of collaboration. **Figure 3** shows that a collaborative landscape has emerged in this field with the United States and China as dual cores. The connection between the two countries is the thickest, indicating that Sino-U.S. collaboration is the primary axis of transnational cooperation in this field. Additionally, the green cluster centers on China and connects with developing countries such as Brazil and Iran; the yellow cluster centers on the United States and connects with developed countries such as Sweden and Germany; the red cluster exhibits characteristics of regional cooperation among South Asia, the Middle East, and Southeast Asia (India-Saudi Arabia-Singapore); and the blue cluster represents a collaboration circle among traditional academic powerhouses in Europe and Oceania (France-Australia-Canada). The total link strength between the United States and China (23,004 vs. 21,860) far exceeds that of other countries (**Table 1**), constituting the primary bridge for global collaboration. Some countries with lower publication volumes (such as Singapore, Malaysia, Austria, and Iran) also occupy a place in the collaboration network, indicating that research in the interdisciplinary field of machine learning and triple-negative breast cancer is attracting participation from multiple regions worldwide. This demonstrates a dual nature of both North-South cooperation and regional collaboration, with the geographical scope of research collaboration continuing to expand.

**Figure 3:**
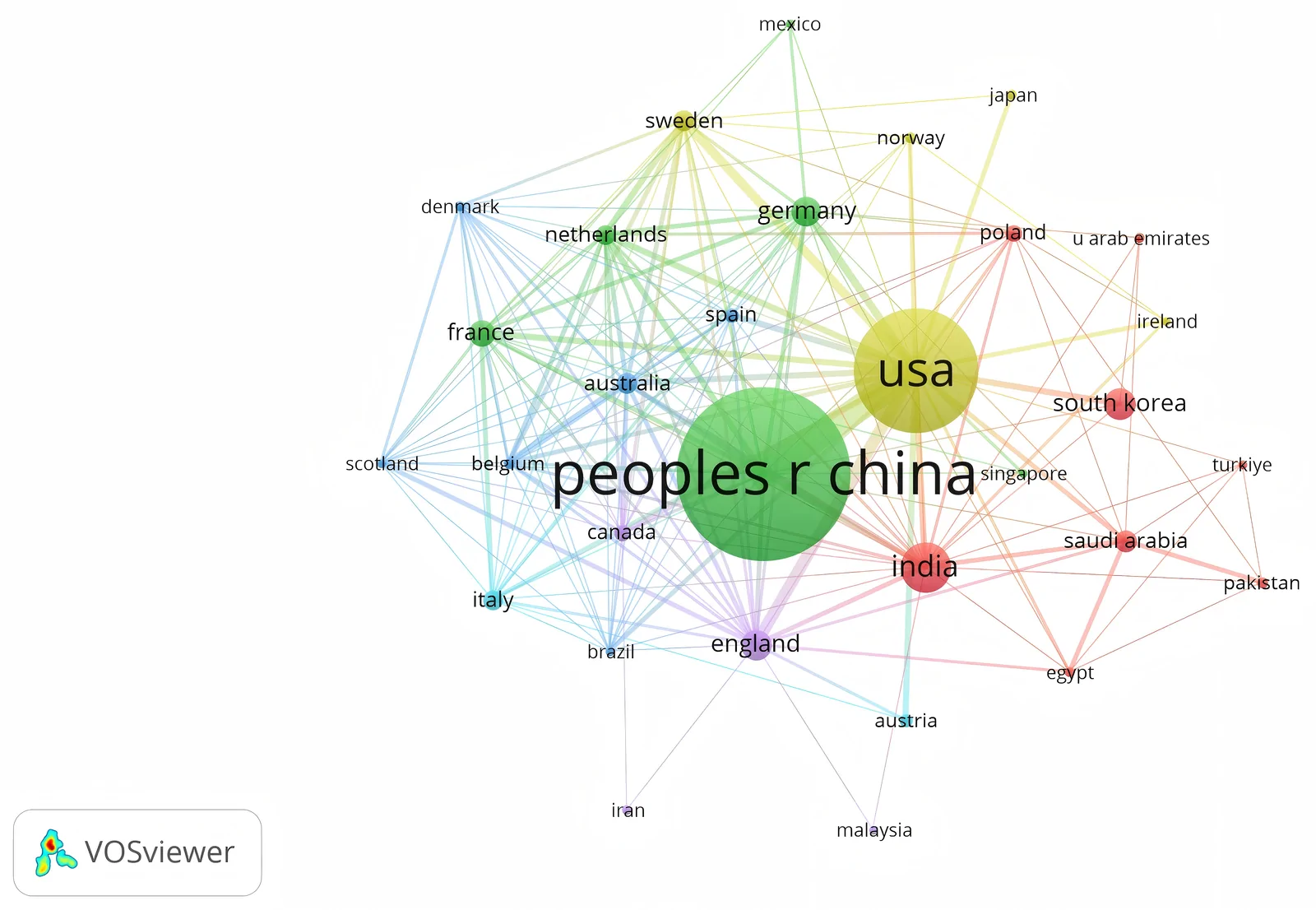
Co-authorship network of countries (threshold: ≥ 2 documents per country).

#### 3.2.2 Institutions

To reveal direct research collaboration relationships among institutions, this study conducted a co-authorship-based network analysis using VOSviewer. Prior to the analysis, institutional names were standardized and merged to reduce node fragmentation caused by multiple names for the same institution. A threshold was set at ≥3 publications and ≥10 total citations per institution; 77 institutions meeting these criteria were included in the network (**Figure 4**). The network reveals that Chinese institutions form a tight-knit collaborative cluster, with core nodes including Sun Yat-sen University, Fudan University, Sichuan University, and Southern Medical University. U.S. institutions such as Stanford University, Baylor College of Medicine, and Case Western Reserve University exhibit transnational collaborative links with Chinese institutions; however, the overall density of international collaboration is lower than that of domestic collaboration.

**Figure 4:**
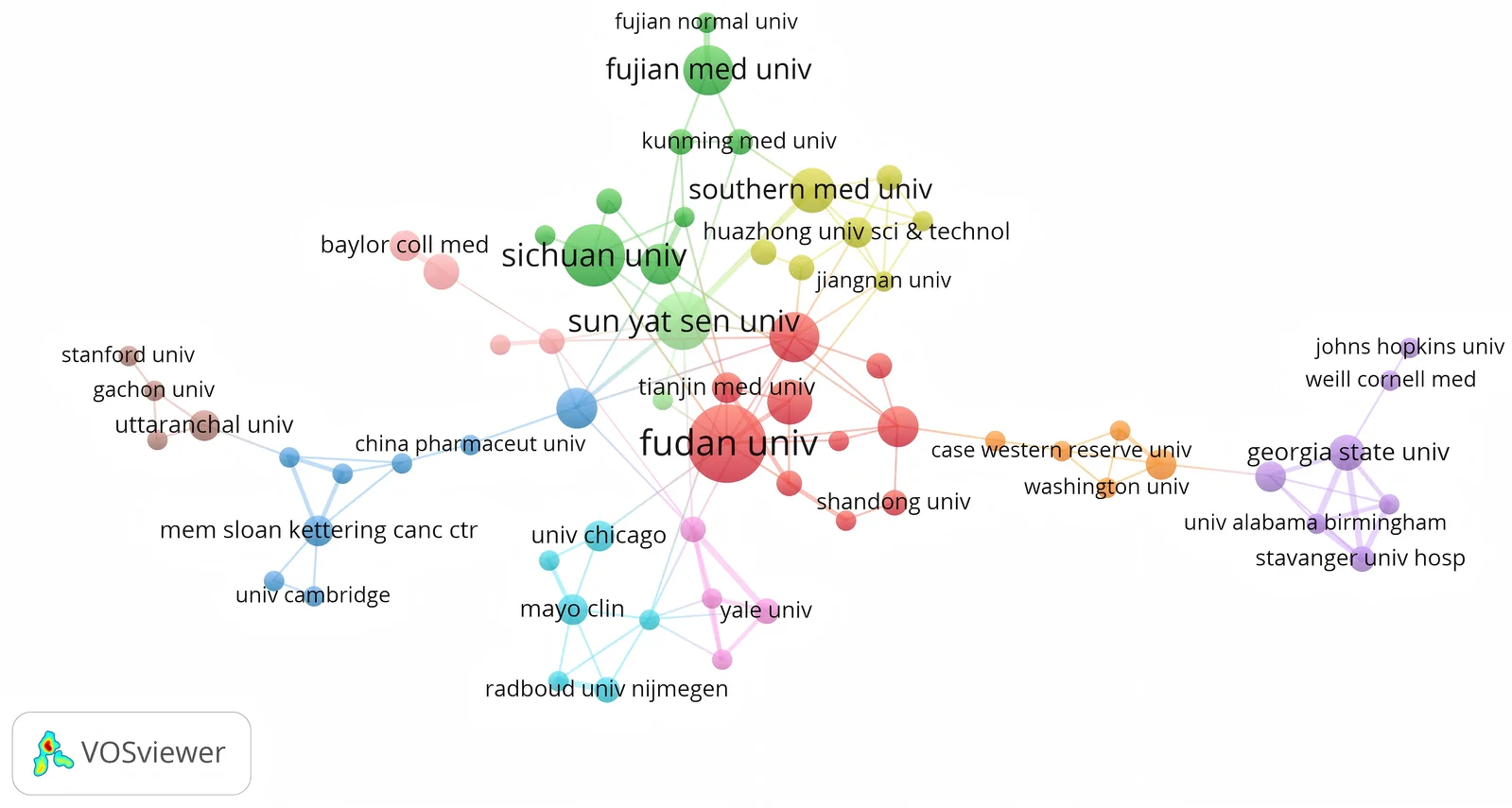
Co-authorship network of institutions (threshold: ≥ 3 documents and ≥ 10 citations).

However, while institutional mergers improve the readability of the network structure, they may obscure the independent output characteristics of high-impact subunits. To ensure the assessment of top research institutions remains unbiased, this study used Biblioshiny to separately calculate the total number of publications for each institution (without any threshold) without merging institutional names. **Table 2** lists the top ten institutions by publication volume. The University of Texas System (57 papers) and MD Anderson Cancer Center (42 papers) rank first and second, demonstrating the high output of leading U.S. cancer centers in this field; Fudan University (39 papers) and Sichuan University (38 papers) follow closely behind, while European institutions such as Unicancer in France (34 papers) and the University of London (30 papers) are also active. This ranking indicates that research at the intersection of machine learning and triple-negative breast cancer exhibits a three-way balance of output among the United States, China, and Europe. Although Chinese institutions are numerous, U.S. and European institutions also hold significant positions. A comparison of the collaboration network (**Figure 4**) and the output ranking (**Table 2**) reveals that while MD Anderson Cancer Center ranks second with 42 publications, it does not form an independent node in the collaboration network. This suggests that while grouping MD Anderson under the University of Texas System to reduce node noise prior to the collaboration network analysis was effective, it also created an information gap: **Table 2** shows MD Anderson as a highly productive and active research institution, but **Figure 4** fails to reflect its true cross-regional collaboration characteristics. Therefore, regarding the specific collaboration patterns of sub-institutions such as MD Anderson, this study cannot draw conclusions based on the current network data.

**Table 2:** Top 10 most productive institutions.

Rank	Institution	Articles
1	UNIVERSITY OF TEXAS SYSTEM	57
2	UTMD ANDERSON CANCER CENTER	42
3	FUDAN UNIVERSITY	39
4	SICHUAN UNIVERSITY	38
5	UNICANCER	34
6	UNIVERSITY OF LONDON	30
7	SUNYATSEN UNIVERSITY	24
8	HARVARD UNIVERSITY	22
9	FUJIAN MEDICAL UNIVERSITY	21
10	INSTITUT NATIONAL DE LA SANTEET DELARECHERCHE MEDICALE(INSERM)	21

While **Table 2** reveals the publication volume and geographic distribution of leading institutions, it does not distinguish between clinically affiliated hospitals or cancer centers and pure computational or academic research units. To assess whether the translational gap between algorithm development and clinical validation stems from an institutional disconnect, we classified the corresponding author affiliations of all 394 studies into four broad categories based on institutional primary focus (**Supplementary Table 9**): clinical or medical affiliated (hospitals, cancer centers, clinical departments, and medical universities), academic or research (general universities and research institutes), engineering or technical (pure engineering and computer science departments), and other or industry. Of the 393 studies with identifiable corresponding author affiliations, 233 (59.3%) originated from clinical or medical affiliated institutions, 117 (29.8%) from academic or research institutions, 43 (10.9%) from engineering or technical institutions, and 1 (0.3%) from other or industry entities. The proportion of clinically affiliated institutions increased from 4 studies in 2018 to 83 in 2025, indicating growing involvement of clinical and medical centers in ML-TNBC research. The most productive institutions included Fudan University (11 studies), Sichuan University (8 studies), Fujian Medical University (7 studies), and Shanghai Jiao Tong University (5 studies), all of which were classified as clinical or medical affiliated. Only 2 of the top 15 most productive institutions were classified as academic or research (Sun Yat-sen University, 4 studies) or engineering or technical (University of Electronic Science and Technology of China, 3 studies). This institutional distribution demonstrates that the translational gap in ML-TNBC is not attributable to a lack of clinical involvement in research. Clinical and medical institutions are the dominant contributors to the literature, yet even these clinically affiliated outputs remain predominantly retrospective proof-of-concept studies rather than prospective clinical validations.

At the same time, to assess the knowledge-level connections between institutions (i.e., the frequency with which publications from two institutions are co-cited in the same paper), we conducted an institutional co-citation analysis, setting the following thresholds: institutions must have published at least 5 papers and received a total of at least 10 citations. Of the 22 institutions that met these criteria, the top 10 ranked by total link strength (TLS) are listed in **Table 3**. Sichuan University had the highest total link strength (21), but its citations per paper were only 9.67, indicating that its research findings are frequently cited as background knowledge but have relatively limited influence on a per-paper basis. Sun Yat-sen University ranked second in total link strength (19) and also had the highest citations per paper (61.09) and total citations (672), making it a dual leader in the field as both a knowledge hub and a producer of high-quality output. Uttarachal University (India) achieved a total link strength of 17 with only 5 publications, suggesting that its small number of papers may focus on a highly interconnected topic, positioning it as an emerging knowledge node. Fudan University and Southern Medical University both have high publication volumes and total citations, with a total link strength of 15 each, demonstrating stable knowledge influence. U.S. institutions (Emory University, Georgia State University) also rank among the top ten, indicating their significant presence in the co-citation network. Combining the collaboration network diagram (**Figure 4**) with the co-citation analysis (**Table 3**) reveals that institutions with close collaborations (such as Sichuan University and Sun Yat-sen University) often exhibit high co-citation centrality. However, some institutions (such as Sichuan University) demonstrate stronger co-citation performance than their collaboration performance, while others (such as Fudan University) show balanced performance across both analyses. These differences reveal that different institutions play distinct roles in this field.

**Table 3:** Top 10 organizations ranked by total link strength based on co-citation analysis.

Rank	Organization	Documents	Citations	Average citations	Total link strength
1	Sichuan University	12	116	9.67	21
2	SunYat-sen University	11	672	61.09	19
3	Uttaranchal University	5	56	11.2	17
4	Fudan University	16	493	30.81	15
5	Southern Medical University	8	292	36.5	15
6	Emory University	5	93	18.6	9
7	Georgia State University	6	103	17.17	9
8	Tianjin Medical University	5	40	8	9
9	Harbin Medical University	7	82	11.71	8
10	Huazhong University of Science and Technology	5	30	6	8

### 3.3 Journal analysis

We used Biblioshiny to calculate each journal’s h-index, g-index, m-index, number of publications (NP), total citations (TC), and 2025 JCR impact factor (IF) to assess their local influence in the field of machine learning research on triple-negative breast cancer. **Table 4** lists the top 10 most influential journals in this field. Cancers ranks first, demonstrating the highest publication output and cumulative impact. Frontiers in Oncology and Scientific Reports rank second and third, respectively. Although Nature Communications and Advanced Science have limited publication volumes, they exhibit significant citation impact, indicating that the articles they publish have extremely high individual impact. Meanwhile, Breast Cancer Research, as a specialized journal on breast cancer, maintains relatively stable influence in this field.

**Table 4:** Top 10 most impactful journals in machine learning and TNBC research (2011–2026).

Source	h_index	g_index	m_index	TC	NP	PY_start	IF(JCR)
CANCERS	9	17	1.29	307	24	2020	4.0
FRONTIERS IN ONCOLOGY	8	10	1.33	277	10	2021	3.5
SCIENTIFIC REPORTS	7	15	1.00	251	22	2020	3.8
FRONTIERS IN IMMUNOLOGY	5	8	0.83	97	8	2021	5.3
NATURE COMMUNICATIONS	5	7	0.83	280	7	2021	14.7
BREAST CANCER RESEARCH	4	5	0.5	42	5	2019	5.9
ADVANCED SCIENCE	3	3	0.5	87	3	2021	14.3
ANNALS OF SURGICAL ONCOLOGY	3	3	0.38	33	3	2019	3.5
BMC CANCER	3	4	0.43	22	5	2020	3.4
BREAST CANCER RESEARCH AND TREATMENT	3	7	0.2	111	7	2012	3.0

The column named “PY_start” stands for “Year of first publication”; for the infinite loop values in m_index, round to two decimal places.

The co-citation network of journals constructed using VOSviewer (**Figure 5**) reveals four major clusters within the field’s knowledge base. The yellow cluster, centered around the *Journal of Clinical Oncology* (the largest node at the network’s center), along with *Lancet Oncology*, *Cancers*, and *Annals of Oncology*, represents the mainstream journals in clinical oncology and constitutes the foundation of clinical evidence. *The New England Journal of Medicin*e, *The Lancet*, and *Nature Medicine* are also included in this cluster, indicating that the research has broad medical relevance. The red cluster includes *Cancer Research*, *Clinical Cancer Research, Frontiers in Immunology, Cancer Cell*, and *Nature Reviews Cancer*, reflecting the dimensions of basic cancer research and immunotherapy. The green cluster includes breast cancer specialty journals (*Breast Cancer Research and Treatment, NPJ Breast Cancer)* and medical imaging journals (*Radiology, European Radiology*), reflecting research in disease-specific and diagnostic dimensions. The blue cluster covers basic biomedical and computational biology journals (*Nature, Cell, Radiomics, Nucleic Acids Research*), highlighting the algorithms and biological foundations of machine learning applications. The dense connections between clusters underscore the highly interdisciplinary nature of this research field.

**Figure 5:**
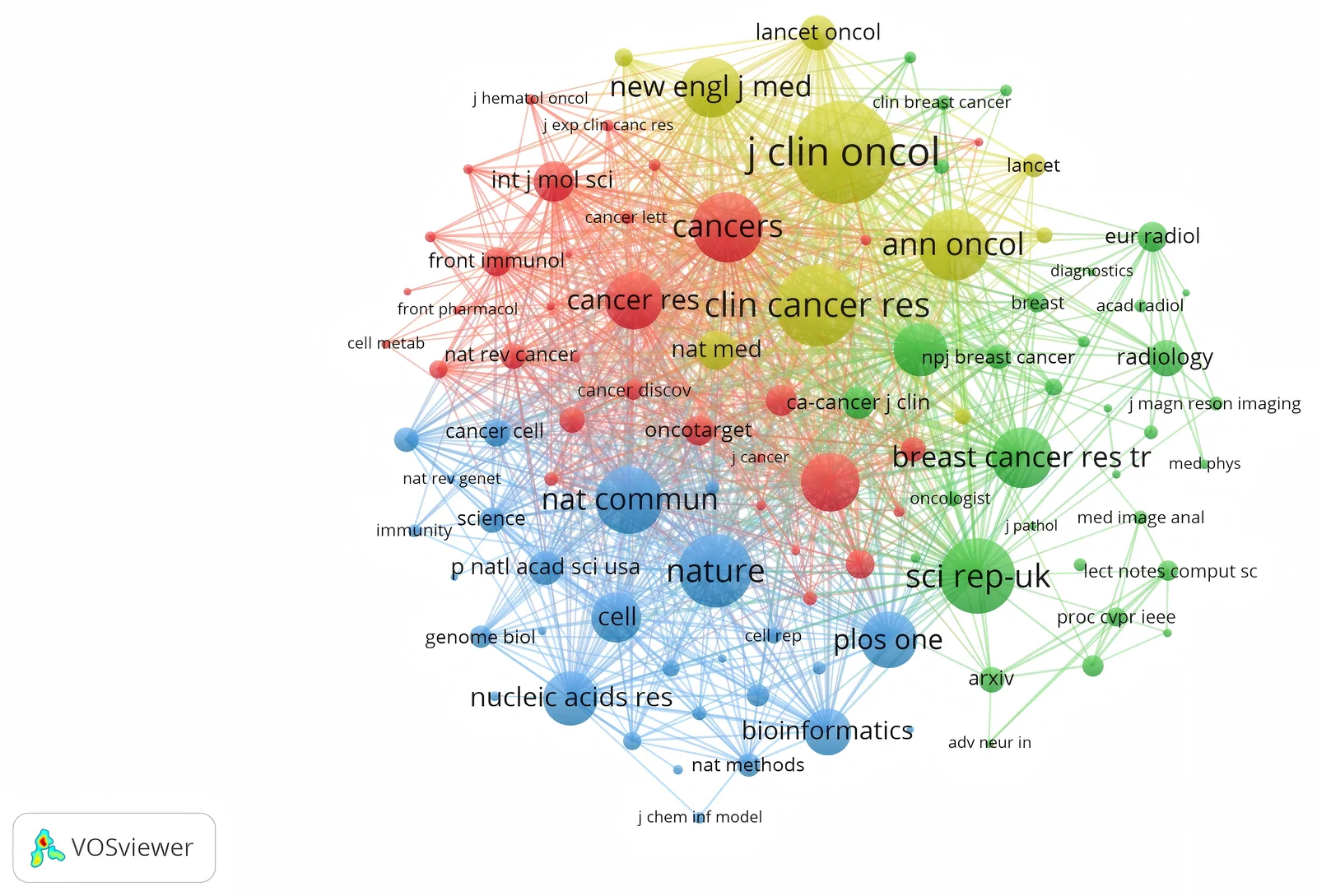
Journal co-citation network. Only journals with ≥ 40 citations are shown (104 sources).

### 3.4 Author analysis

**Table 5** lists the top 10 most influential authors in the field of machine learning research on triple-negative breast cancer, ranked by h-index. Ciompi F and Jiang Y are tied for first place (h-index = 5), but their academic output patterns differ significantly: Ciompi F has a higher total citation count (TC = 288), indicating that his research has a broader academic impact; Jiang Y, despite having the same number of publications (NP = 6), has a relatively lower total citation count (TC = 107); combined with a later start year (PY_start=2021), this suggests that his research may be in the early stages of building influence. Chen C ranks at the top with the highest total citations (TC = 751). Although his h-index is 4, his average citations per paper reach 150.2, indicating that his published articles include high-impact, foundational work. Among them, Janssen EAM and Kong J (both of whom began publishing in 2022) have rapidly accumulated academic influence in a short period (h-index = 4), indicating that their research has garnered significant immediate attention.

**Table 5:** Top 10 authors with the highest h-index.

Author	h_index	g_index	m_index	TC	NP	PY_start
Ciompi F	5	6	0.625	288	6	2019
Jiang Y	5	6	0.833	107	6	2021
Aneja R	4	4	0.571	89	4	2020
Bhattara S	4	4	0.571	89	4	2020
Chen C	4	5	0.444	751	5	2018
Cooper Lad	4	4	0.571	184	4	2020
Hartman J	4	5	0.571	201	5	2020
Huang Y H	4	4	0.667	211	4	2021
Janssen Eam	4	5	0.8	97	5	2022
Konj J	4	4	0.8	64	4	2022

This study used VOSviewer to analyze author collaboration networks, setting the inclusion threshold at authors who had published at least two papers and received at least 10 citations. Of the 2,806 authors, 155 (5.5%) met the criteria; this low proportion reflects a high concentration of research activity, with a small number of authors publishing frequently and collaborating closely, while the majority remain isolated or on the periphery. To further enhance the visibility of clusters and improve the interpretability of the network structure, the network file exported from VOSviewer was optimized using Pajek (v6.02). The Kamada-Kawai algorithm was applied to adjust node positions, ensuring that clusters with strong internal cohesion were spatially aggregated while preserving the original collaborative relationships and connection strengths. The optimized network was then re-imported for final visualization (**Figure 6**).

**Figure 6:**
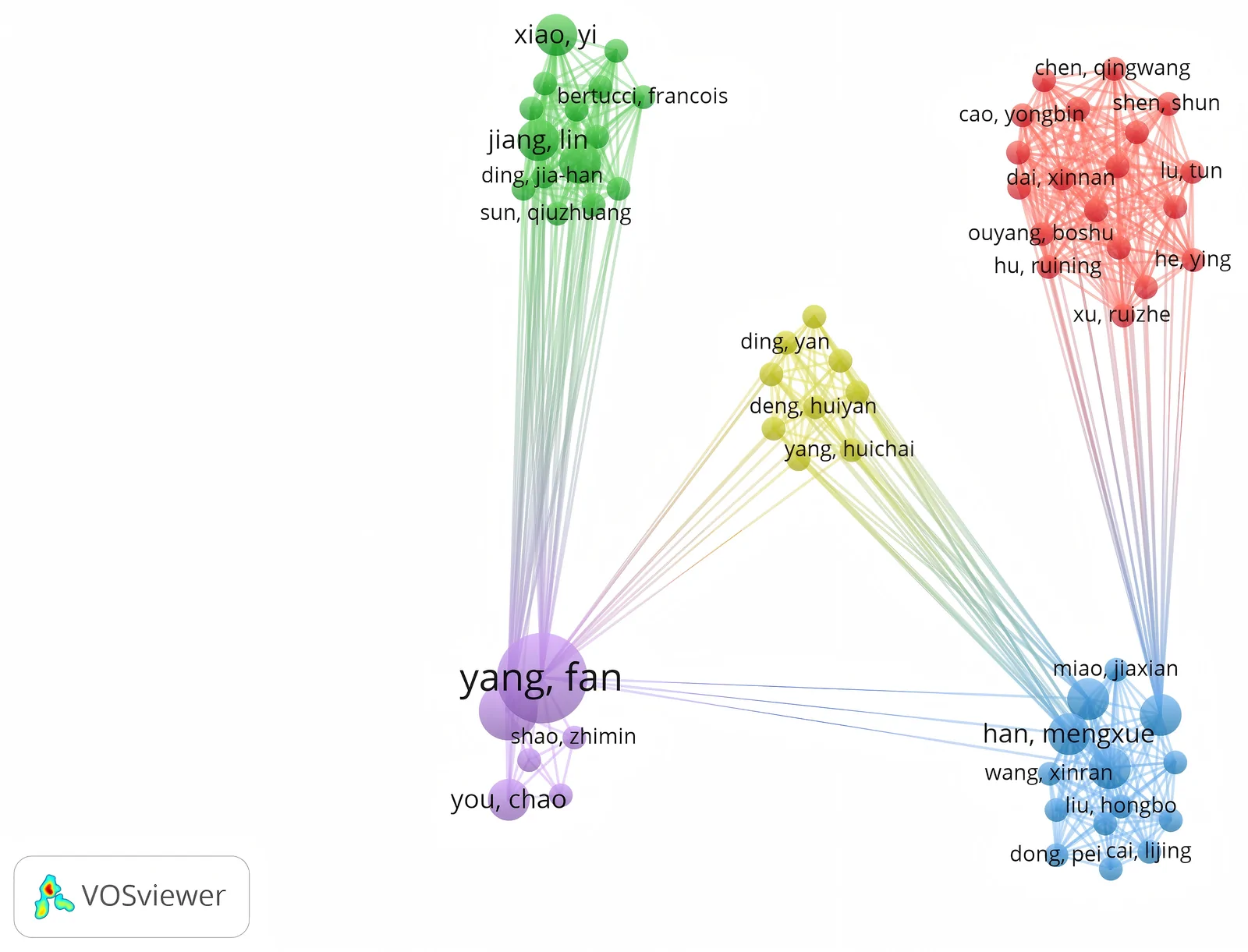
Co-authorship network of researchers in machine learning and TNBC research.

An analysis of the authors’ collaboration network (**Figure 6**) reveals the organizational structure of research teams in this field. The network can be divided into five major clusters: Purple cluster: Centered around Yang F, this cluster includes Shao Z M, You C, and others. It occupies a central position in the network, connecting multiple research teams, which demonstrates its pivotal role in the field; Green cluster: Represented by Xiao Y and Bertucci F, this cluster reflects characteristics of international collaboration; Red cluster: Primarily composed of Chinese scholars such as Chen Q W, Shen S, and Cao Y B, this is the largest research team in the field; Yellow cluster: A small collaborative group represented by Ding Y, Deng H Y, and Yang H C; Blue cluster: Another active research group represented by Miao J X, Han M X, and Wang X R. Network analysis reveals that connections within each cluster are tight, but cross-cluster collaboration is relatively sparse, suggesting that synergy among different research teams needs to be strengthened. This structural characteristic indicates that the field remains in a stage where research teams operate independently; future integration of the field could be advanced by promoting cross-team and cross-institutional collaboration.

### 3.5 Keyword co-occurrence and trend evolution

To gain insight into the research hotspots and knowledge structure in this field, we conducted a keyword co-occurrence analysis using VOSviewer (**Figure 7**). In the figure, node size represents the frequency of a term’s occurrence, while line thickness indicates the strength of co-occurrence. The network centers around “triple-negative breast cancer” and “machine learning,” forming six clusters that broadly correspond to three domains: disease understanding, methodological innovation, and clinical translation. Currently, high-frequency terms remain concentrated on disease concepts (TNBC, breast cancer) and AI methods (machine learning, deep learning), indicating that current research primarily focuses on applying ML tools to TNBC problems and has not yet fully established a closed-loop process where clinical needs drive methodological innovation.

**Figure 7:**
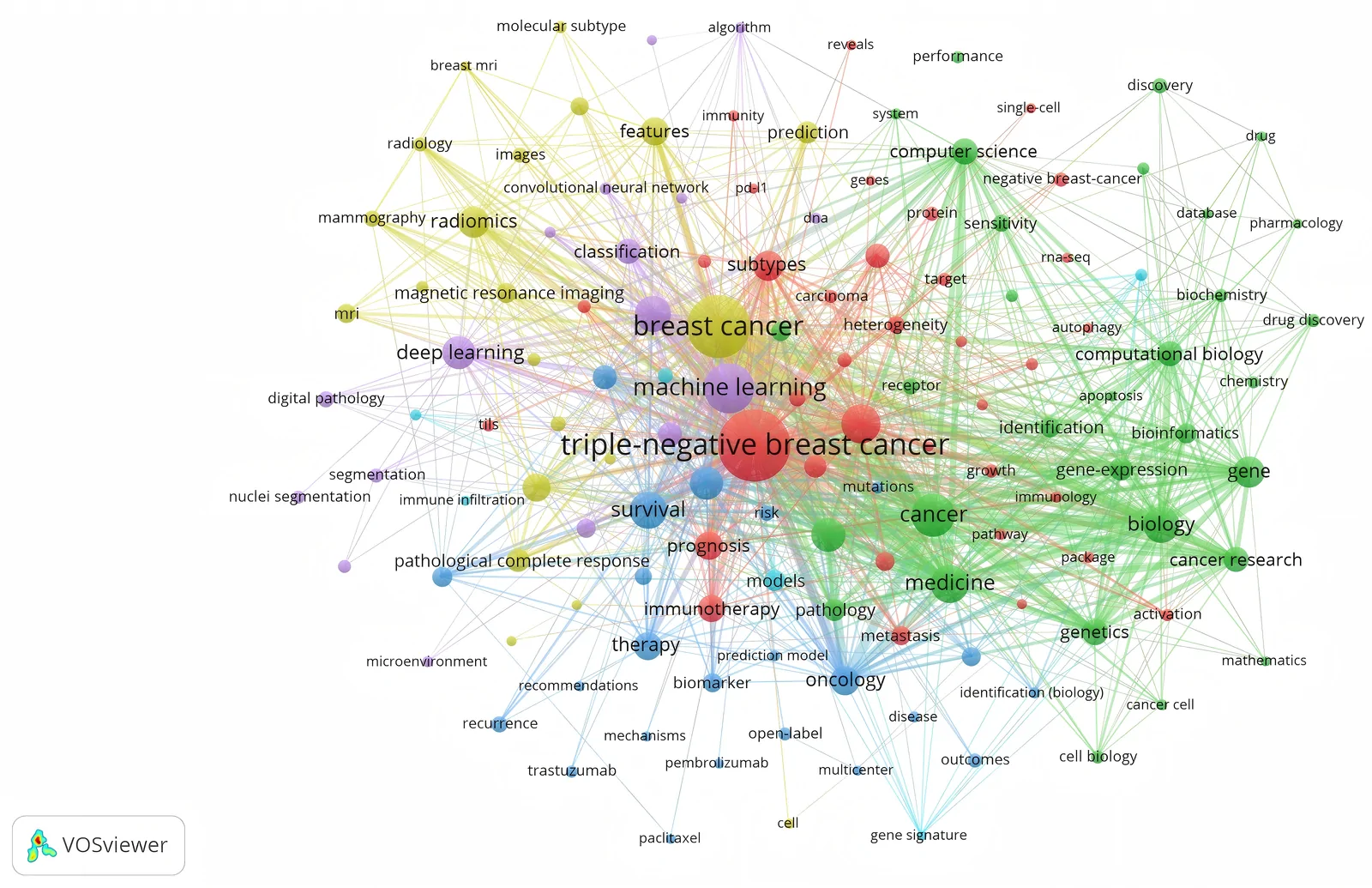
Co-occurrence keyword network.

The red cluster centers on the TNBC disease ontology, with keywords extending from subtype classification to the tumor microenvironment (tumor microenvironment, immunotherapy, PD-L1, TILS), as well as metabolic reprogramming, metastasis and resistance, and single-cell analysis (single-cell, RNA-seq). This reflects the shift in TNBC research from traditional histological classification toward a precision understanding based on molecular characteristics and the immune microenvironment.

The green cluster represents the foundational layer with the broadest scope of connections within the network, bringing together computational biology, radiomics, and computer science alongside cancer research, cell biology, genetics, and pharmacology. This co-occurrence pattern indicates that innovation in TNBC machine learning research stems not only from algorithmic iterations but also relies on computational modeling of tumor biological mechanisms. The presence of “internal medicine” and ‘medicine’ indicates that these computational studies remain firmly anchored in a clinical medical context rather than being purely engineering-driven. The co-occurrence of “drug discovery” and “random forest” suggests that machine learning has begun to enter the early stages of drug repurposing and target discovery.

The purple cluster centers on machine learning, deep learning, and artificial intelligence, connecting outward to specific algorithms such as convolutional neural networks and support vector machines, as well as applications such as digital pathology and image segmentation, thereby forming the methodological core. The yellow cluster, meanwhile, forms a radiomics chain extending from image acquisition (mammography, ultrasound, MRI) to feature extraction (radiomics, features) and on to clinical prediction (prediction, pathological complete response). These two clusters are closely intertwined, illustrating that radiomics serves as the primary data carrier and clinical interface for AI methods in the TNBC field. Within the yellow cluster, neoadjuvant chemotherapy and pathological complete response occupy central positions, anchoring image analysis to the specific clinical decision-making context of neoadjuvant therapy rather than general screening or auxiliary diagnosis.

The dark blue cluster focuses on the evaluation of treatment response and long-term prognosis, with chemotherapy, survival, and recurrence as its core themes. It encompasses concepts such as precision medicine and prediction models, and also includes specific drugs such as pembrolizumab, trastuzumab, and paclitaxel. The presence of “multicenter” and “outcomes” indicates that multicenter validation and clinical outcome evaluation have entered the research landscape, but the solitary occurrence of “open-label” suggests that randomized controlled trials have not yet reached a significant scale. This cluster connects to the red cluster via ‘biomarker’ and to the yellow cluster via “prediction model,” forming a pathway from disease characterization to treatment decision-making.

The light blue cluster is the smallest, centered on biomarkers, gene signatures, the transcriptome, and immune infiltration, and focuses on biomarker discovery and immune phenotype identification. However, it is located on the periphery of the network and has weak connections to the deep learning methods in the purple cluster and the image feature extraction in the yellow cluster. This suggests that biomarker research based on the immune microenvironment and the transcriptome has not yet established effective algorithmic integration with mainstream AI-driven imageomics.

Overall, the green and red clusters constitute the upstream knowledge generation layer, providing biological hypotheses and computational tools; the purple and yellow clusters form the midstream technology translation layer, bridging the gap between multimodal data and predictive models; and the dark blue cluster represents the downstream clinical decision-making layer, linking treatment response and prognosis management. Meanwhile, the light blue cluster remains isolated on the periphery, revealing that the current discovery and validation of biomarkers have not yet been effectively integrated into the machine learning-driven multimodal integration process. In other words, although multimodal data and deep learning methods are widely applied, research on prospective biomarker validation and clinical trial design has not yet achieved knowledge integration spanning from method development to clinical decision support, and a significant knowledge gap remains.

To reveal the dynamic evolution of the research topic, this study used CiteSpace to generate a timeline view of keyword co-occurrence clusters (**Figure 8**). The horizontal axis represents the years (2011–2026), and the vertical axis represents cluster IDs (a total of 12 valid clusters were identified, distinguished by different colors). Node size indicates keyword frequency, and the lines reflect co-occurrence strength. Detailed characteristics of each cluster (including the silhouette score, average year, LLR labels, and mutual information terms) are provided in **Supplementary Table 1**. Evaluation shows that the silhouette score for all clusters are >0.8, indicating that the clustering structure is reliable. Based on the temporal periods of activity for each cluster, the evolution of the field can be divided into four phases.

**Figure 8:**
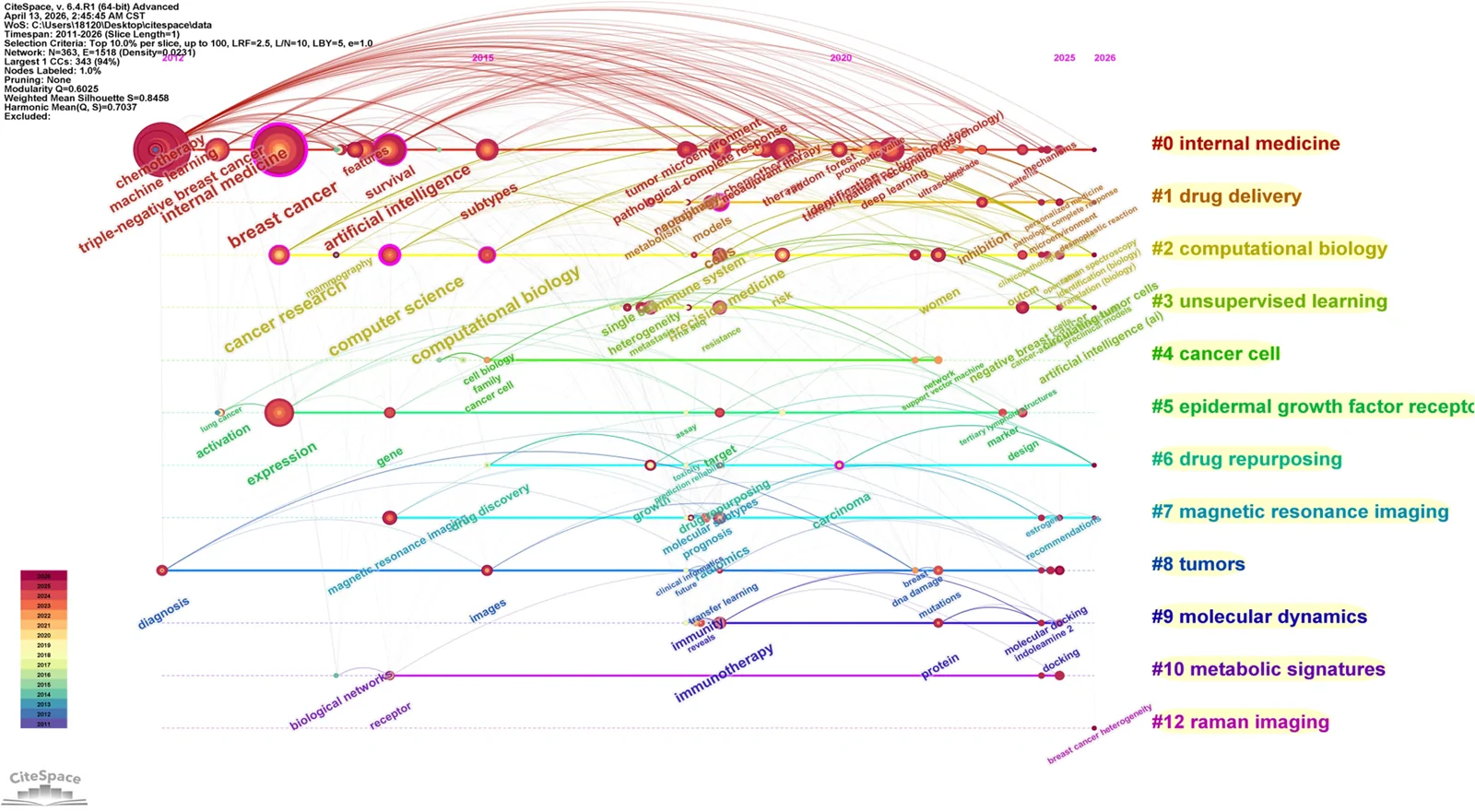
Timeline view of keyword clusters.

1. Early Foundational Phase (2011–2015): The active clusters during this phase primarily included #4 cancer cell, #5 epidermal growth factor receptor, #0 internal medicine, and #4 cancer cell. These clusters focused on tumor cell biology, cytoskeletal dynamics, and signal transduction pathways. Their LLR-specific terms included cancer cell, cell biology, and transcription factor, while LSI analysis also revealed terms such as differential equation and signal transduction, indicating that early research was dominated by mathematical modeling and molecular and cellular experiments. #5 epidermal growth factor receptor centers on the EGFR signaling pathway, involving EGFR inhibitors, cancer stem cells, and virtual screening, reflecting early exploration of TNBC driver genes and targeted therapies. #0 Internal Medicine: Anchored by internal medicine and clinical questions, terms such as “internal medicine,” “pathological complete response,” and “neoadjuvant therapy” have already appeared in the LLR, suggesting that awareness of clinical endpoints was established early on. During this phase, research methods were primarily traditional experiments and simple mathematical models, with machine learning not yet widely integrated.

2. Methodological Integration and the Rise of Single-Cell Omics (2015–2020): Two key methodological clusters emerged during this phase: #2 computational biology and #3 unsupervised learning. Within the LLR for #2 computational biology, “computational biology” achieved the highest score across the entire dataset and was highly integrated with terms such as “precision medicine,” “cancer research,” and “machine learning,” marking the systematic integration of computational biology and precision medicine. The specific terms in the LLR for #3 Unsupervised Learning were “unsupervised learning,” “single-cell RNA sequencing,” and “metformin,” reflecting a paradigm shift toward using unsupervised learning methods to analyze single-cell transcriptomic data to explore TNBC heterogeneity, while new applications of the established drug metformin also became a hot topic. Furthermore, #10 “metabolic signatures” and #8 “tumors” focus on metabolomic features and pan-cancer issues, respectively. During this phase, machine learning and single-cell technologies began to reshape research paradigms.

3. The Boom in Translational Medicine and Multimodal Integration (2020–2022): This was the phase with the highest density of cluster emergence, during which multiple clusters directly related to clinical translation appeared in concentrated clusters. Some early nodes in #6 drug repurposing, #7 magnetic resonance imaging, #9 molecular dynamics, and #1 drug delivery. The top term in the LLR for #6 drug repurposing is “drug repurposing,” and the LSI points to WEE1 kinase and its inhibitors, indicating that AI-driven drug repurposing has emerged as an independent research direction. In the LLR of #7 Magnetic Resonance Imaging, “magnetic resonance imaging” serves as the core, integrating with radiogenomics, axillary lymph node metastasis, and other concepts to form a complete chain from image acquisition to lymph node metastasis prediction, formally establishing radiogenomics as an independent knowledge module. The LLR of #9 Molecular Dynamics focuses on the intersection of molecular dynamics simulation and immune checkpoints TDO/IDO, representing the cutting-edge convergence of computational chemistry and tumor immunology. #1 Drug Delivery is characterized by drug delivery, nanomedicine, and precision oncology, gradually shifting its focus toward nanomedicine delivery systems. This phase simultaneously advances methodologies, imaging, pharmaceuticals, and immunology, with the integration of multimodal data and deep learning algorithms as the mainstream approach. Research objectives have shifted from understanding diseases to developing intervention strategies.

4. Emerging Specializations and Clinical Translation Challenges (2023–2026): The most active clusters during this phase are #1 drug delivery and #12 Raman imaging. In the LLR for #1 Drug Delivery, “drug delivery” ranks first, while the MI features highly novel terms such as “mitophagy agonist” and “aptamer-siRNA conjugates.” With an average publication year of 2024, this indicates that cutting-edge research is focusing on smart nanodelivery systems and the regulation of mitochondrial autophagy. #12 Raman Imaging is a perfectly homogeneous emerging cluster, with core concepts including Raman imaging, metabolic reprogramming, therapeutic resistance, and acrotealatin A. This cluster represents Raman spectroscopy-based single-cell metabolic phenotyping technology used to elucidate drug resistance heterogeneity in TNBC, making it one of the most forward-looking directions in the field.

From 2011 to 2026, machine learning research on TNBC has undergone a clear evolution: “cellular/signaling pathway fundamentals → computational biology and single-cell omics → radiomics and drug repurposing → nanodelivery and Raman metabolic phenotyping.” The research focus has gradually shifted from basic biology toward translational medicine, and methodologies have transitioned from traditional mathematical modeling to deep learning-driven multimodal integration. However, none of the terms related to prospective clinical validation—such as “clinical trial,” “randomized controlled trial,” “prospective validation,” or “multi-center external validation”—appeared in the LLR, LSI, or MI metrics of any of the aforementioned clusters. Even the most recent cluster #12 is entirely devoid of such terms. This gap aligns closely with our subsequent quantitative analysis, which found that only 24 retrospective validation studies (6.1%) and 9 registered prospective trials (2.3%) were identified among 394 computational publications. This indicates that while the field is thriving in areas such as algorithms, imaging, and drug delivery, the systematic lag in clinical validation has become a critical bottleneck constraining translation.

A close-up view (**Figure 9**) focusing on 2025–2026 reveals that current cutting-edge research is characterized by a dual-track approach driven by “microscopic environment analysis” and “spectroscopic techniques.” On the one hand, the continuous interplay between immune cells (macrophages, T cells), matrix components (cancer-associated fibroblasts), and immune infiltration in the tumor microenvironment with molecular subtypes and MRI, combined with explorations of personalized therapy involving trastuzumab and personalized response, indicates that precision strategies targeting the microenvironment and molecular subtyping remain a core direction; The reappearance of “docking” and “metformin” suggests that computational modeling and research on repurposing existing drugs continue. On the other hand, Raman spectroscopy and Raman imaging, along with spectroscopy, circulating tumor cells, and breast cancer heterogeneity, form an emerging technological cluster. Combined with preclinical models and translational biology, this reflects the ongoing development of a transition from label-free metabolic phenotyping to liquid biopsy. Notably, the sporadic appearance of “open label” as a clinical trial design term coexists with clinical endpoint terms such as “recommendations” and “efficacy,” yet the weight of “preclinical models” remains far greater than that of prospective validation terminology. Overall, this zoomed-in view further confirms that while the field shows nascent signs of translation, such as individualized responses and open-label studies, key components like randomized controlled trials and multicenter external validation are still absent.

**Figure 9:**
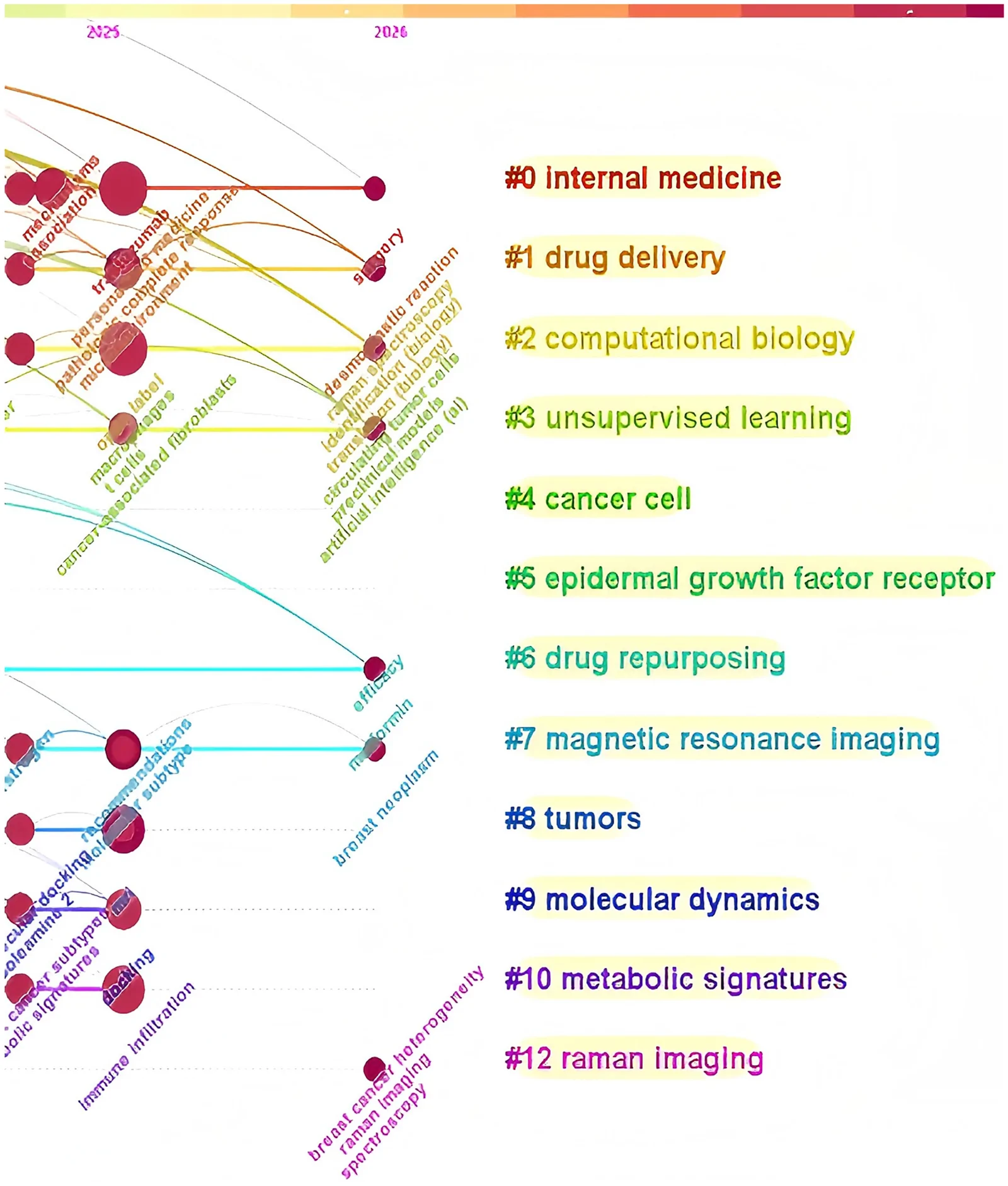
Enlarged timeline view of the 2025-2026 period.

To examine annual changes in keyword popularity from a macro perspective, this study used Biblioshiny to generate a trend topic map (**Figure 10**). With the year on the x-axis and the annual frequency of keyword occurrences (normalized) represented by bubble size, the figure visually illustrates the shifting focus of research.

**Figure 10:**
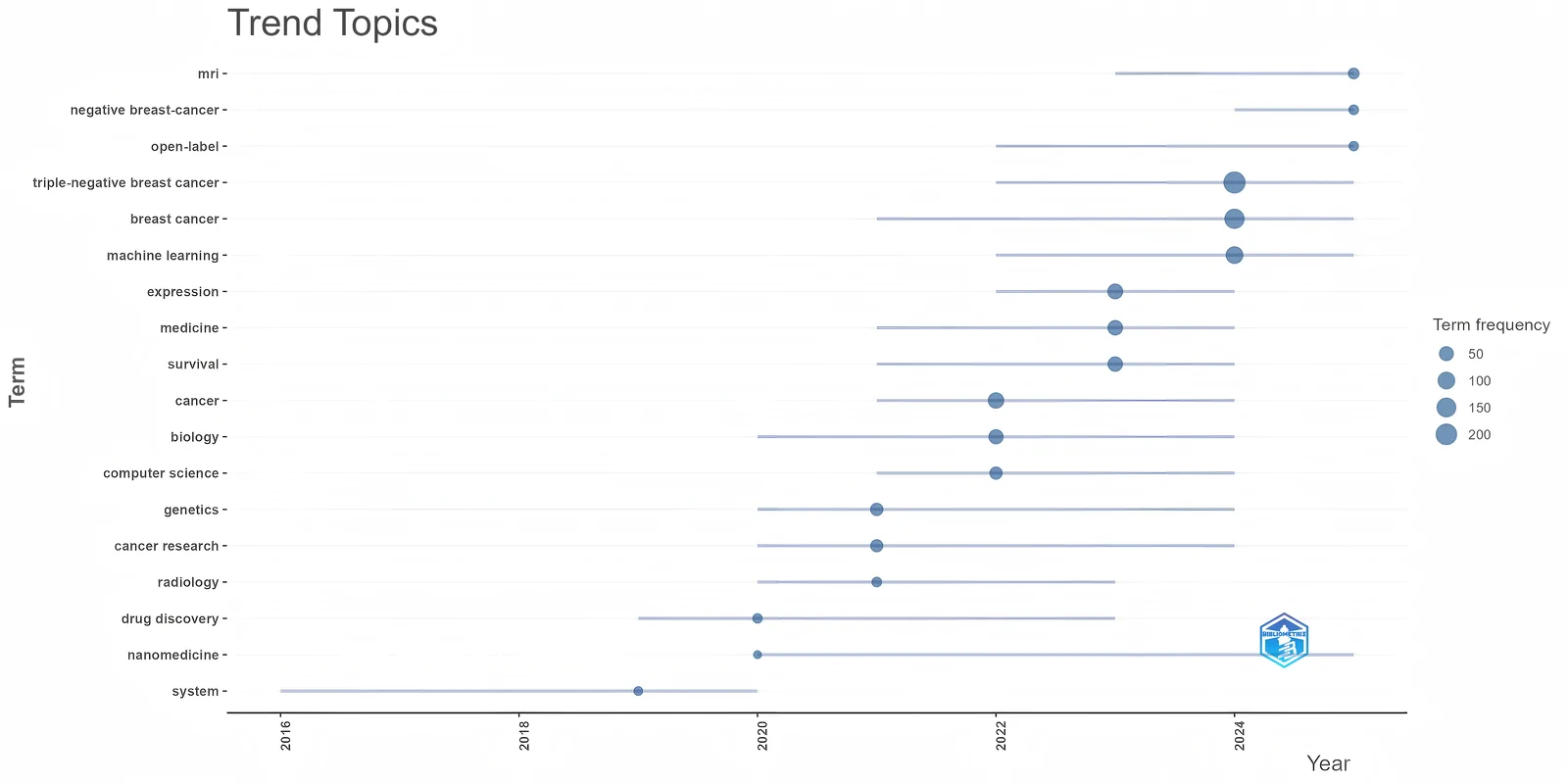
Trend topics of keywords overtime.

As shown in **Figure 10**, “triple-negative breast cancer” and “breast cancer” have consistently been the most frequently occurring keywords, peaking in 2024. “Machine learning” has been steadily gaining traction since 2016, with its frequency approaching that of “breast cancer” in 2024, indicating that machine learning has become an indispensable analytical tool in this field. Basic biological terms such as “expression,” “biology,” “genetics,” and “cancer research” maintained a consistently high frequency between 2020 and 2024, reflecting the continued focus on research into molecular mechanisms. The expansion of the “radiology” bubble is corroborated by the high frequency of imaging-related terms such as “radiomics,” “MRI,” “mammography,” and “ultrasound” within the yellow cluster, suggesting that imaging-driven machine learning analysis is a growing application area in this field. The expansion of the “drug discovery” and “nanomedicine” bubbles, meanwhile, indicates that these areas of drug development are beginning to attract attention.

Compared to the CiteSpace burst table (**Figure 11**), the trend topic map places greater emphasis on sustained popularity rather than sudden spikes. For example, “machine learning” appears frequently over the long term in the trend topic map, but because it is a core search term that appears consistently at high frequency, it does not appear in the emergence table; conversely, “tumor in filtrating lymphocytes” has a small bubble in the trend topic map (indicating low absolute frequency) but shows high-intensity emergence in the emergence table, indicating that it is a rapidly rising emerging research direction. The two analytical methods are complementary and together provide a comprehensive picture of research hotspots.

**Figure 11:**
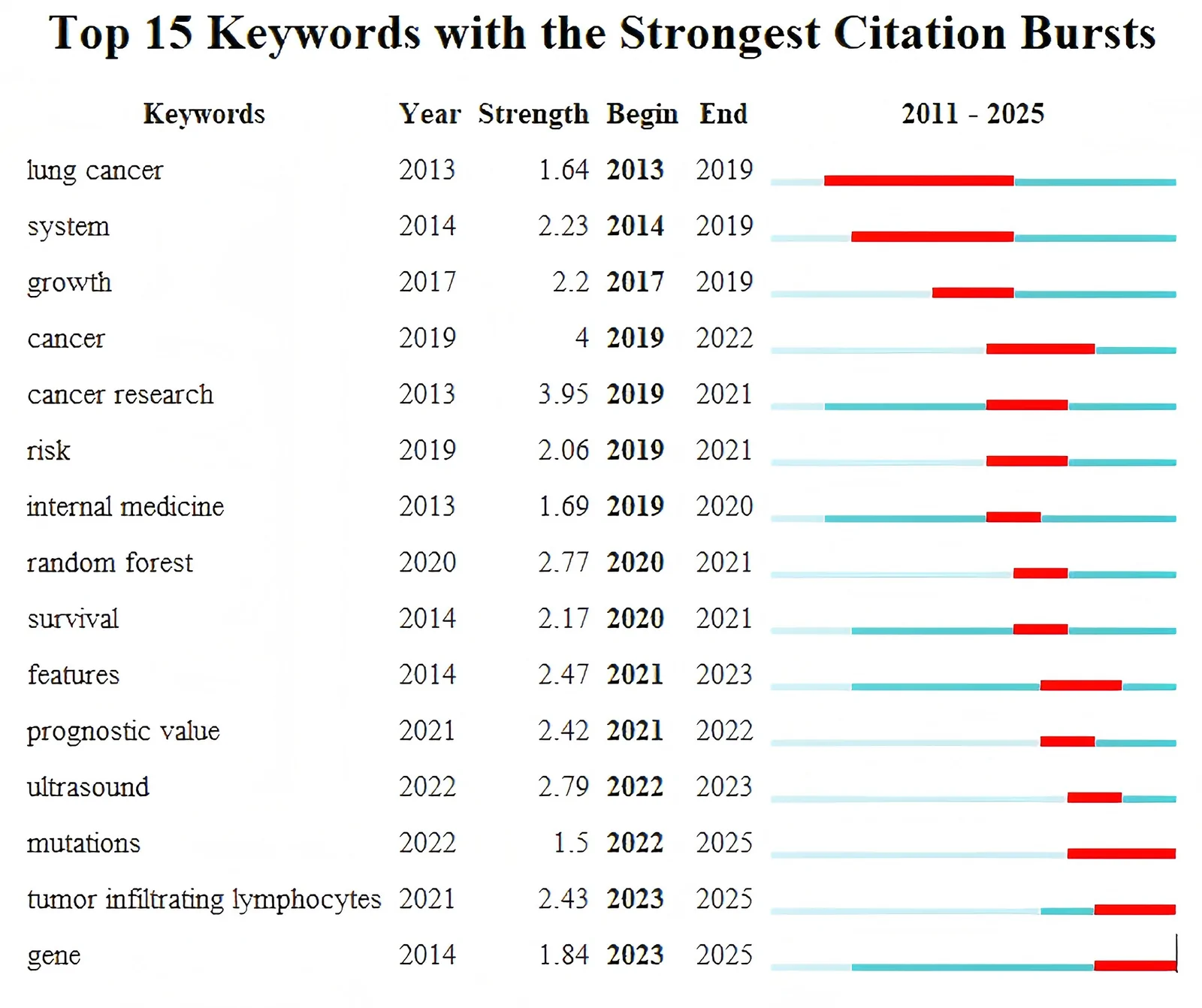
Top15 keywords with the strongest citation bursts (2011–2025).

**Figure 11** shows keywords that experienced a sudden increase in citation intensity (emerging terms), reflecting the evolution of research hotspots. The period is divided into three phases: Early (2011–2015): The emerging terms were “classification” (2011–2015) and “system” (2014–2019), indicating that the field initially focused on basic classification algorithms and system development. Mid-phase (2017–2022): “growth,” “cancer research,” “risk,” “random forest,” “prediction,” “selection,” “cancer,” “features,” and “ultrasound” emerged successively. Among these, “random forest” (2020-2021) had an intensity of 2.7, reflecting the widespread attention given to ensemble learning methods during this phase; the emergence of “ultrasound” (2022–2023) suggests that ultrasound imageomics is beginning to emerge as a hot topic. Recent period (2023–2026): “gene,” “tumor-infiltrating lymphocytes,” “neoadjuvant therapy,” and “gene expression” (1.74) have emerged as the latest prominent terms. This aligns closely with the activity observed at the 2025 node in the timeline diagram, collectively indicating that gene expression profiles, tumor-infiltrating lymphocytes, and the prediction of neoadjuvant therapy response are currently the most dynamic research directions.

Meanwhile, “machine learning” and its synonyms (such as “deep learning” and “artificial intelligence”) did not appear in the list of emerging terms. This is primarily because, as core search terms, these terms maintained a consistently high frequency throughout the entire time span, exhibiting a linear rather than sudden growth pattern; thus, they did not meet the emergence criteria of the Kleinberg algorithm. This indirectly confirms that machine learning persists as a common method in this field, while the list of emerging terms more effectively reveals the phased rise of specific application scenarios (such as “neoadjuvant therapy”) and particular techniques (such as “random forest”) against the backdrop of the widespread adoption of these methods. This provides a more precise depiction of the trajectory of research frontiers.

### 3.6 Co-citation clustering and evolution

**Table 6** lists the 10 most frequently cited papers in this field. Among them, the study by Keren L et al., published in *Cell* in 2018, ranks first. This paper utilized machine learning methods to analyze the spatial organization of the breast cancer tumor microenvironment, laying the foundation for subsequent spatial transcriptomics and immune infiltration analyses. A review by He Y et al. published in *J Exp Clin Cancer Res* in 2018 systematically summarized the application of machine learning in prognostic prediction for TNBC. Although the multi-omics integrated prognostic prediction study by Zou Y T et al. published in 2022 in *Int J Surg* and the radiomics study by Almansour NM et al. published in 2022 in *Front Mol Biosci* were published relatively recently, they have already entered the top ten in a short period of time, reflecting the high impact of their recent research.

**Table 6:** Top10 articles with the highest citations.

Author	Year	Journals	DOI	TotalCitations	TCperYear	NormalizedTC
KEREN L	2018	CELL	10.1016/j.cell.2018.08.039	764	84.89	3.95
HE Y	2018	JEXPCLINCANCRES	10.1186/s13046-018-1002-1	465	51.67	2.40
ZOU Y T	2022	INTJSURG	10.1016/j.ijsu.2022.106936	324	64.80	8.47
ALMANSOURNM	2022	FRONTMOLBIOSCI	10.3389/fmolb.2022.836417	269	53.80	7.03
XIAO Y	2022	CELLRES	10.1038/s41422-022-00614-0	241	48.20	6.30
MEDINA MA	2020	INTJENVRESPUBHE	10.3390/ijerph17062078	238	34.00	4.87
MEYERA S	2013	SCISIGNAL	10.1126/scisignal.2004155	228	16.29	1.00
SAHA A	2018	BRITJCANCER	10.1038/s41416-018-0185-8	216	24.00	1.12
TAFTAFR	2021	NATCOMMUN	10.1038/s41467-021-25189-z	179	29.83	4.99
THAKURV	2019	JEXPCLINCANCRES	10.1186/s13046-019-1443-1	142	17.75	3.79

A clinical appraisal of the 10 most-cited papers reveals a marked disconnect between citation impact and clinical validation maturity. Among these 10 papers, three were narrative reviews without primary patient data (7, 20, 21). Three were basic science studies that employed machine learning for image segmentation or protein interaction analysis but did not evaluate patient-level clinical prediction endpoints (22–24). Only four studies developed patient-level predictive models. Of these, two used public database cohorts (TCGA) with cross-validation or split-sample internal validation (25, 26), one performed metabolomic subtyping on 330 patients with preclinical organoid validation but no prospective clinical testing (27), and one applied machine learning to 922 breast cancer DCE-MRI examinations for molecular subtype prediction, achieving an AUC of only 0.654 for triple-negative breast cancer (28). Notably, none of the 10 most-cited papers reported independent external validation across multiple centers, and none were prospective interventional trials. This indicates that citation frequency in this field does not correlate with clinical validation strength; the most visible research outputs are predominantly retrospective proof-of-concept studies or reviews rather than clinically validated implementations.

Based on the co-citation network analysis conducted using CiteSpace, a total of 8 knowledge clusters were identified. Among these, 7 major knowledge clusters (size ≥ 29) were included in the visualization analysis (**Figure 12**) (Modularity Q = 0.8408, weighted average silhouette coefficient = 0.9184), indicating that the cluster structure is significant and the partitioning is reasonable. Detailed characteristics of each cluster (including size, silhouette coefficient, average year, representative publications, and top terms) are presented in **Supplementary Table 2**. Based on the average year and cluster terms, the seven major clusters exhibit a distinct three-stage evolutionary pattern.

**Figure 12:**
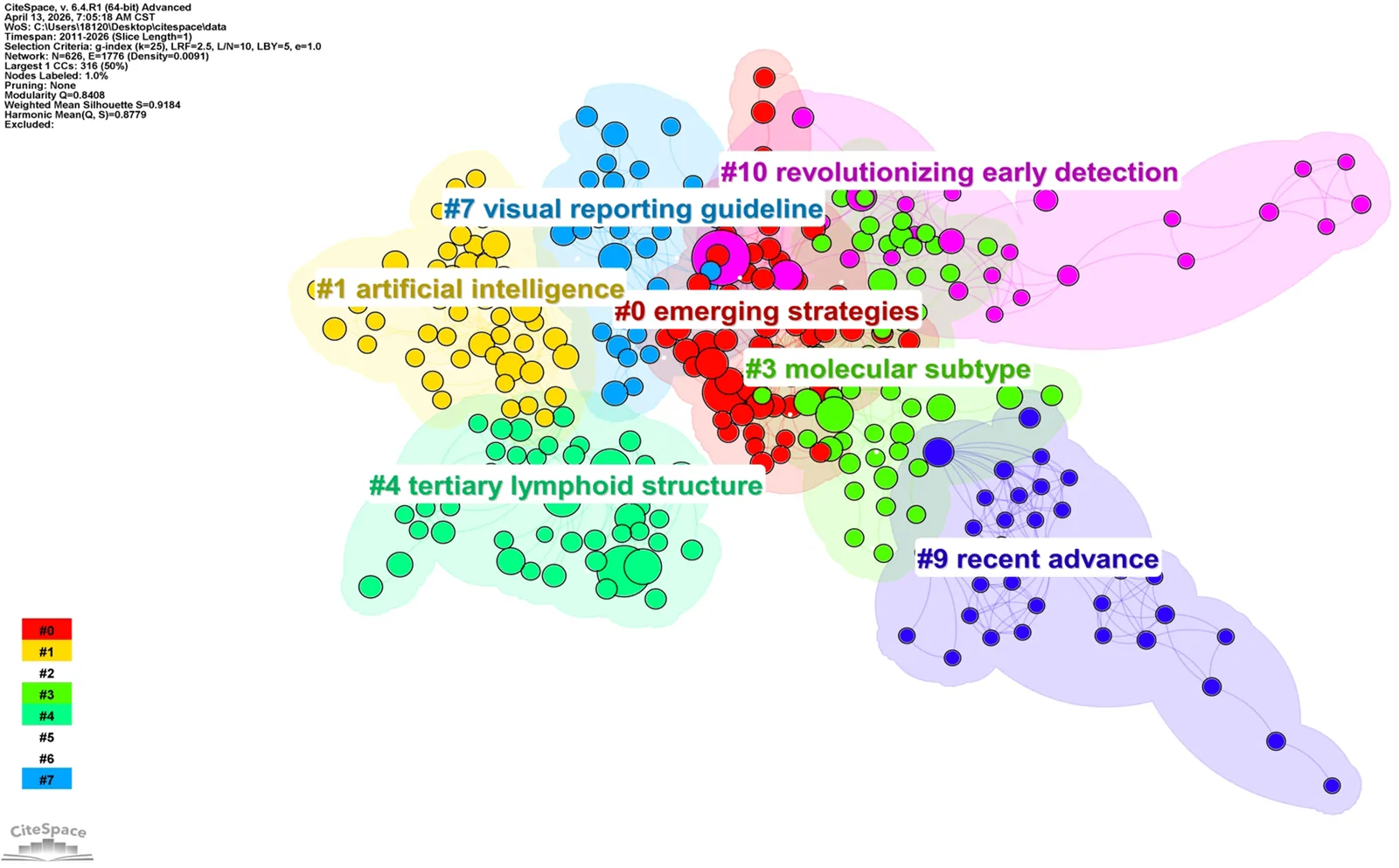
Cluster map of co-cited references.

Phase 1 (2015–2017) was the foundational framework establishment phase: Cluster #9 had the earliest average year (2015) and served as the core source of the knowledge base in this field. Its top terms focus on “triple-negative breast cancer treatment,” “neoadjuvant chemotherapy,” “epigenetic subtype,” and “transcriptomic change,” indicating that early research concentrated on treatment strategies for TNBC, responses to neoadjuvant chemotherapy, and the identification of epigenetic subtypes. This cluster has an extremely high coefficient of variation (0.999), suggesting a high degree of internal consistency within this foundational theme and extremely clear research boundaries. Cluster #7 builds upon this foundation, focusing on “computational tumor-in-filtering lymphocyte (TIL) assessment,” “visual reporting guideline,” and “open-source automated TIL algorithm.” This represents the beginning of standardization in computational pathology methods for tumor-infiltrating lymphocyte assessment during this phase, laying the methodological groundwork for subsequent AI-assisted pathological diagnosis.

Phase 2 (2019–2020) marked the period of AI integration: Clusters #1 and #3 emerged simultaneously, representing the two major pathways through which AI entered TNBC research. The top terms in Cluster #1 were “artificial intelligence,” “infiltrating lymphocyte,” “learning-based assessment,” and “computational pathology approaches,” reflecting the deep integration of AI and computational pathology, particularly in the applications of automated TIL assessment and breast cancer subtype prediction. Cluster #3, on the other hand, focuses on “molecular subtype,” “pathological complete response (pCR),” and “radiomics-based machine learning models,” demonstrating the clinical implementation of radiomics-driven ML models in molecular subtyping and preoperative pCR prediction. Additionally, although Cluster #17 is very small in scale, its theme focuses on “prediction of pCR to neoadjuvant systemic therapy using deep learning on multiparametric MRI.” It can be viewed as a precise subset of Cluster #3, representing the highly specialized field of using deep learning and MRI to predict pCR.

Phase 3 (2021–2022) represents a period of deepened clinical translation: Cluster #0, as the largest cluster, is located at the center of the network. Its top terms include “emerging strategies,” “pathologic complete response,” “therapeutic approaches,” and “molecular pathway,” suggesting that the field has entered a phase of treatment optimization involving multiple parallel strategies. Cluster #4, meanwhile, focuses on “tertiary lymphoid structure (TLS),” “single-cell analysis,” and “TH2-associated microenvironment,” reflecting that the refined analysis of the immune microenvironment has become a new growth area in research: tertiary lymphoid structures serve as biomarkers for immunotherapy response, while single-cell technologies are used to reveal tumor heterogeneity. Finally, Cluster #10 has the latest average publication year. The themes “revolutionizing early detection,” “next-generation diagnostic strategies,” and “non-invasive biomarkers” point toward innovations in early detection and non-invasive diagnostics, indicating that the field is beginning to take a forward-looking approach toward preventive medicine.

In summary, the evolution of knowledge in this field has progressed step by step from basic therapeutic frameworks → the integration of AI with pathology and imaging → innovations in the refinement of the immune microenvironment and early detection, with Cluster #0 serving as a central hub that spans multiple stages, reflecting the continuous optimization of treatment strategies.

An analysis of citation bursts (**Figure 13**) identified a total of 25 key publications exhibiting significant citation bursts. These publications, which remain active (2023–2026), collectively point to the prediction of immunotherapy response, deep learning-driven radiomics, and precision medicine as the most cutting-edge research directions, which is highly consistent with the results of the keyword burst analysis.

**Figure 13:**
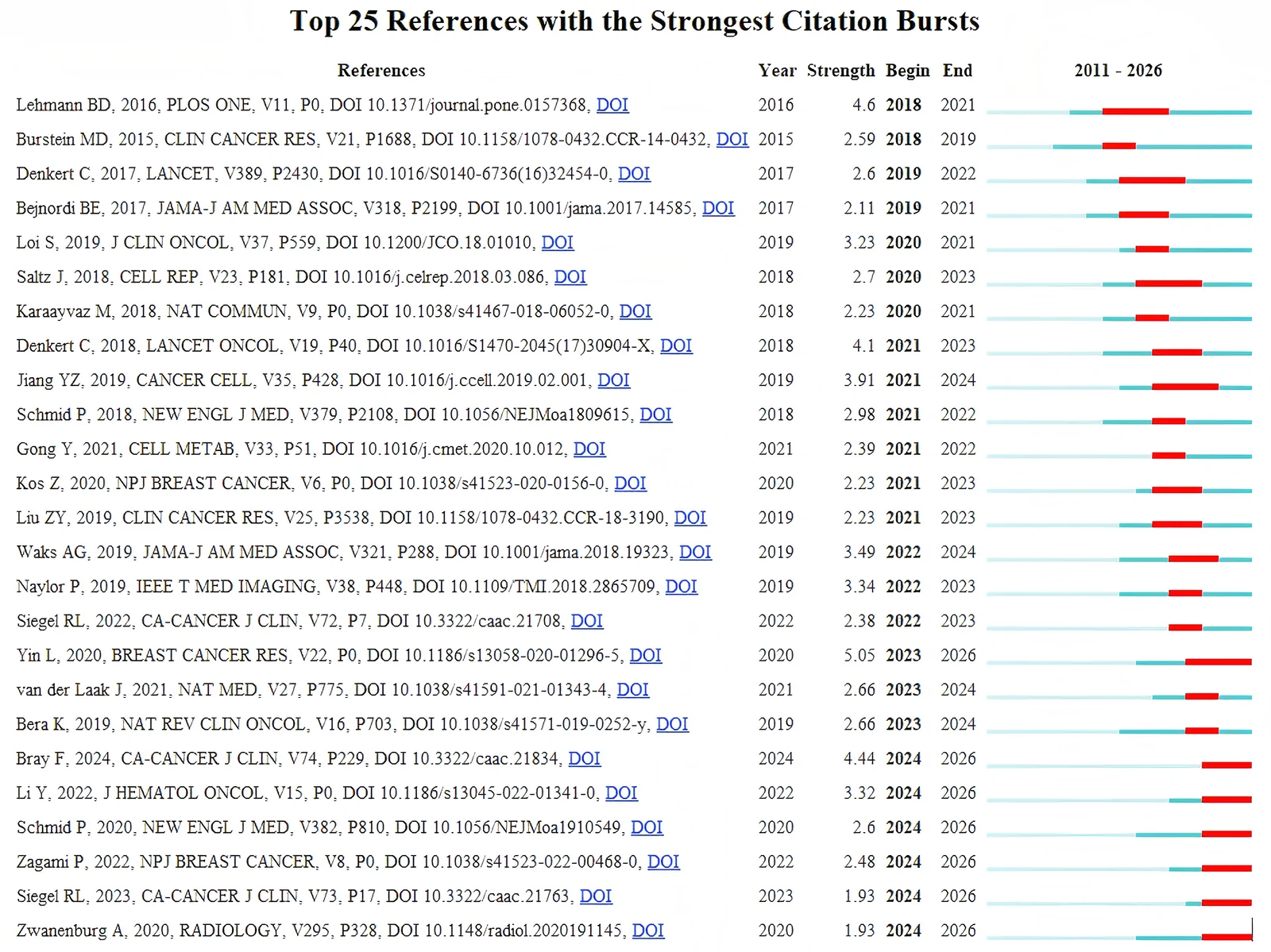
Top 25 references with the strongest citation bursts.

### 3.7 Representative PubMed validation results

To assess the representativeness of the main analysis (WoSCC+Scopus dataset, n=394) and the robustness of the bibliometric conclusions, we conducted an independent validation using the PubMed database. Using a semantically equivalent search strategy mapped to PubMed’s MeSH vocabulary and keyword fields (January 2011–January 2026, English, journal articles/reviews), we identified a total of 531 articles.

#### 3.7.1 Consistency in posting trends

First, we compared the annual publication trends across the two datasets (**Figure 14**). The annual publication trends in the main analysis (WoSCC+Scopus) were highly consistent with those in PubMed, with a Pearson correlation coefficient of r = 0.989 (p < 0.001). Both datasets show a rapid increase in publication volume starting in 2019, peaking in 2025 (121 papers in the main analysis and 127 in PubMed), and then declining to 16 and 34 papers, respectively, in 2026 (based on January data only). This strong correlation indicates that the PubMed validation set and the main analysis dataset follow exactly the same macro-level development trajectory.

**Figure 14:**
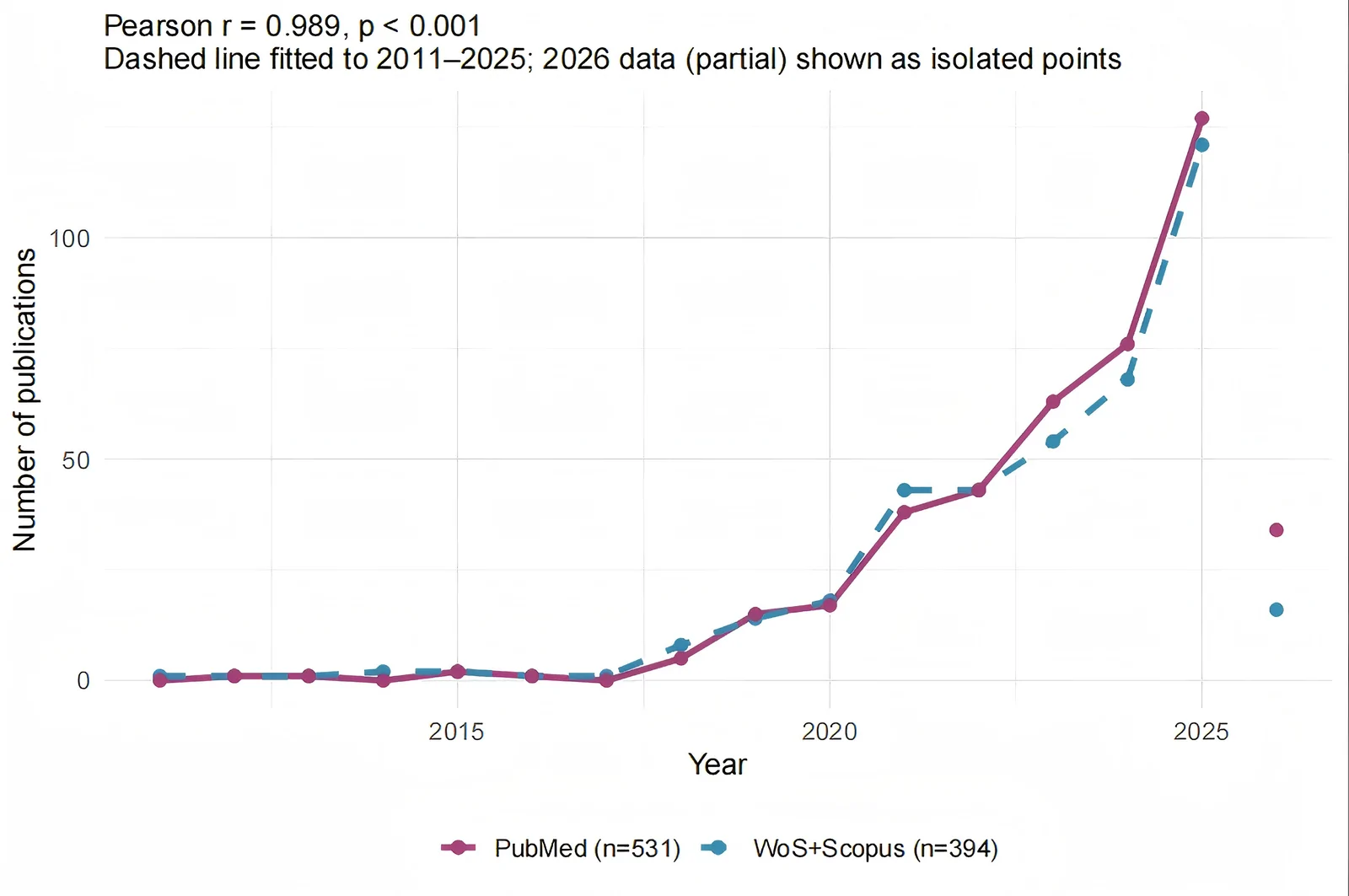
Annual publication trends in PubMed and the WoSCC + Scopus datasets.

#### 3.7.2 Document overlap and features unique to PubMed

Cross-database deduplication between PubMed and the main WOS+Scopus dataset was performed using normalized title substring matching: (i) titles were standardized (lowercased, punctuation removed, whitespace collapsed); (ii) the first 50 normalized characters of each PubMed title were searched against all WOS+Scopus titles; (iii) a match was declared if the normalized prefix appeared in any main-dataset record. This conservative approach identified 351 articles (66.1%) present in both databases, while 180 articles (33.9%) were exclusive to PubMed (**Figure 15A**). Categorization of the PubMed-exclusive articles (**Figure 15B**) revealed that the majority (113 articles, 62.8%) were published in journals not covered by WOS+Scopus, followed by recent publications from 2025–2026 (48 articles, 26.7%), preprints (12 articles, 6.7%), and conference papers (7 articles, 3.9%).This indicates that PubMed includes more very recently published articles as well as non-traditional publication types, complementing the main analysis dataset.

**Figure 15:**
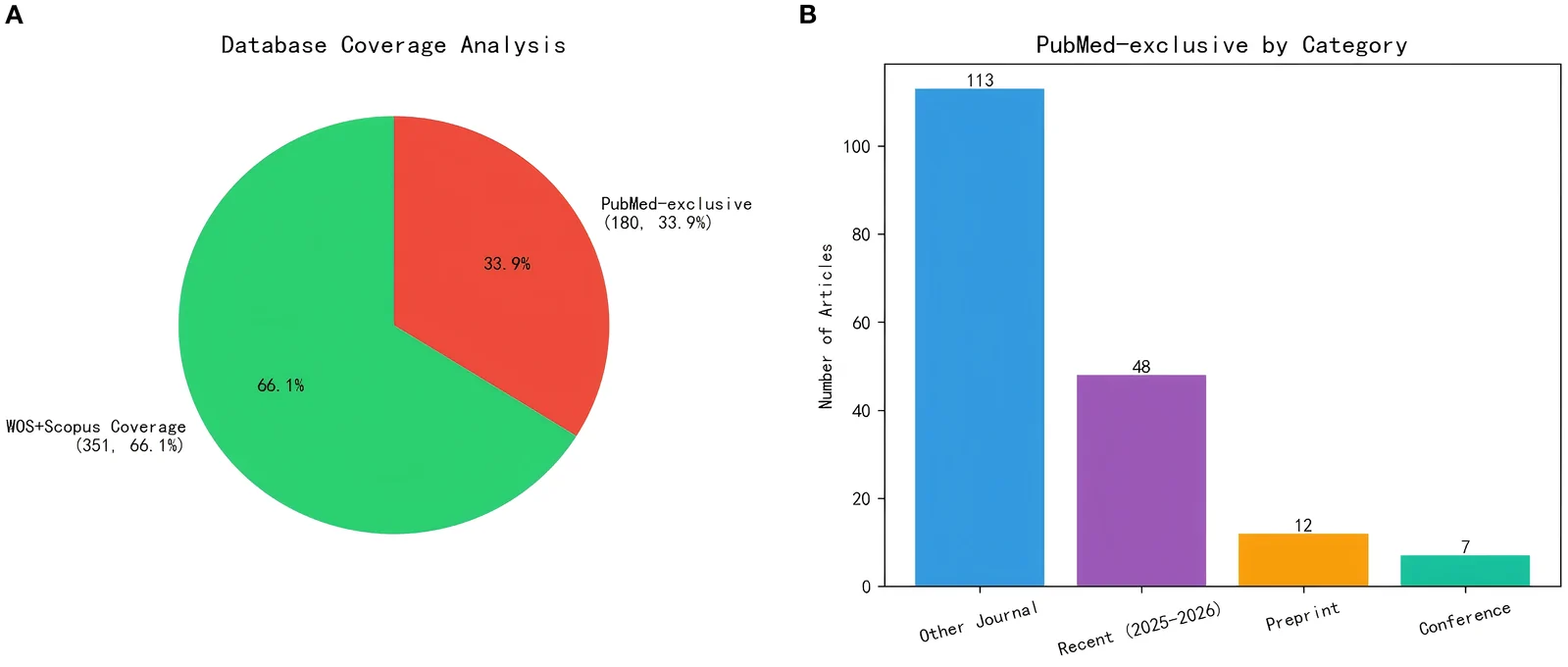
**(A)** Overlap between the two databases. **(B)** Categories of PubMed-exclusive publications.

To further assess the completeness of the dataset’s coverage of high-impact research, we extracted the 50 most-cited core publications from the PubMed search results and used the same title fuzzy matching method (threshold 0.85) to verify their inclusion in the combined Web of Science Citation Categories (WoSCC) and Scopus dataset (**Supplementary Table 3**). The results showed that 31 articles (62.0%) were included, a proportion close to the overall database overlap rate (66.1%). Of the 19 unindexed articles, 12 were preprints, and 7 were published in journals not included in the WoSCC core collection. This result indicates that the WoSCC+Scopus dataset exhibits no significant systematic bias in the inclusion of high-impact research findings and is consistent with overall representativeness.

#### 3.7.3 Coverage of high-impact publications

The 10 most frequently cited articles in the main analysis dataset (**Table 6**) are all available in PubMed, resulting in a coverage rate of 100% (10 out of 10). This indicates that key high-impact research findings have been fully indexed in PubMed, and there are no substantial differences between the two databases in terms of core literature.

#### 3.7.4 Keyword–theme consistency

Following keyword comparison analysis, the Jaccard similarity coefficient between the top 15 high-frequency keywords in WoSCC+Scopus and PubMed was 0.875, indicating a high degree of lexical overlap between the two datasets. The Spearman rank correlation coefficient was ρ = 0.824 (p = 0.0003), indicating a strong positive correlation that is statistically significant. **Supplementary Table 4** presents a detailed comparison of the rankings of the top 15 high-frequency keywords across the two datasets. The top 5 keywords were identical across both datasets, with the primary differences lying in their rankings. These minor discrepancies reflect the inclusion biases of the two databases: PubMed includes a higher proportion of clinical medical literature (e.g., “clinical,” “response”), while WoSCC+Scopus places greater emphasis on methodological papers (e.g., “features,” “based”). Nevertheless, the high consistency of core research themes (Jaccard = 0.875, Spearman’s ρ = 0.824) supports the robustness of the main analysis conclusions based on WoSCC+Scopus across databases.

In summary, the PubMed validation demonstrated that the main analysis dataset (WoSCC+Scopus) is highly representative (66.1% overlap); publication trends are highly synchronized with PubMed (r=0.989); high-impact publications are fully covered (100%); and core thematic keywords show a high degree of overlap (Jaccard = 0.875), confirming that the main analysis conclusions are not subject to database selection bias. PubMed-exclusive publications consist primarily of recently published articles, preprints, and conference papers; these differences do not affect the main conclusions. Therefore, the bibliometric findings based on the main analysis of the WoSCC+Scopus dataset demonstrate a high degree of robustness and generalizability.

### 3.8 Current status of clinical validation of machine learning in triple-negative breast cancer: a PubMed-based analysis

To assess the stage of progress from research to clinical validation of machine learning in triple-negative breast cancer, we initially searched PubMed with publication type restricted to “Clinical Trial” or “Randomized Controlled Trial” (time range: 2011–2026; language: English), retrieving 7 articles. Recognizing that this filter might miss clinically relevant validation studies lacking explicit trial indexing, we conducted an expanded Tier 2 search using clinical validation keywords without publication-type restriction, which retrieved 63 articles. After automated screening (40 excluded) and manual review to verify whether retained articles truly met inclusion criteria (6 excluded for lack of patient cohort validation), 17 additional studies were identified (**Supplementary Table 5**). Combined with the original 7, a total of 24 retrospective validation studies were found in the peer-reviewed literature; no prospective interventional trials were identified.

An analysis of the basic characteristics of the 24 studies (**Supplementary Table 5**) reveals that all were retrospective cohort studies or diagnostic accuracy studies. Sample sizes ranged from 32 to 37,818 cases. Eleven studies employed multicenter designs, while thirteen were single-center or did not specify center status. ML applications fell into four major clinical scenarios.

Radiomics and imaging-based diagnosis. Ten studies utilized radiomics or deep learning models for imaging diagnosis, molecular subtyping, or prognostic stratification based on MRI, mammography, PET, or CT. Three studies focused on molecular subtype classification of TNBC using machine learning with interpretable imaging features (29–31). Two studies applied multiparametric MRI-based deep learning to differentiate TNBC from benign fibroadenoma lesions (32) or to predict prognosis through imaging-based molecular subtyping (33). Five additional studies developed radiomic signatures from MRI, FDG-PET, or CT images for treatment response prediction or survival prognostication (34–38). Notable performance metrics in this category included AUC values of 0.971 for molecular subtype diagnosis (29, 35, 37).

Digital pathology. Four studies leveraged digital pathology and computational histology for immune microenvironment characterization, prognosis prediction, or molecular subtyping. One study used digital pathology combined with immune response analysis in TNBC (39). Two studies integrated digital pathology with multi-omics data for prognosis modeling in large cohorts (40, 41).One additional study combined immune-related digital pathology with clinicopathological features for prognostic stratification (42).

Neoadjuvant chemotherapy response prediction. Six studies focused on predicting pathological complete response or systemic relapse after neoadjuvant chemotherapy using radiomics or deep learning. FDG-PET radiomic signatures were developed to predict treatment response prior to therapy (34) MRI radiomics models demonstrated strong predictive capability for pCR (30, 37, 38). Additional studies used machine learning on imaging or clinicopathological data to model NAC response (36, 43).

Immunotherapy stratification. Four studies explored immune-related biomarkers and immunotherapy response prediction. One study developed automated tumor immunophenotyping to predict clinical benefit from anti-PD-L1 immunotherapy (44). Two studies modeled immune-related prognosis using machine learning on clinical or genomic data (45, 46). One study integrated immune response with treatment response prediction (47).

Other clinical validation scenarios. Five additional studies covered lymph node metastasis prediction (48), large-scale prognosis modeling (49–51), and proteomics-based biomarker discovery (52).

To identify ongoing or registered prospective trials not yet published in the peer-reviewed literature, we searched ClinicalTrials.gov and the WHO International Clinical Trials Registry Platform (ICTRP), identifying 9 registered trials (5 from ClinicalTrials.gov, 4 from WHO ICTRP [ChiCTR]; **Supplementary Table 6**). All 9 trials were observational or diagnostic studies; none were interventional or randomized controlled trials. Among the 5 ClinicalTrials.gov records, 2 are recruiting, 2 have unknown status, and 1 was terminated. Among the 4 WHO ICTRP records, 1 is recruiting and 3 are pending. No completed trials with published results were identified in either registry.

Analysis of ML application scenarios across these 9 trials reveals that imaging-based AI was the most prevalent modality, present in all 9 trials. Treatment response prediction was the second most common application, featured in 6 trials, encompassing neoadjuvant chemotherapy response, immunotherapy response, and recurrence risk assessment. Genomic or multi-omics AI was planned in 5 ClinicalTrials.gov trials, often combined with liquid biopsy (6 trials total). Multimodal AI integration was explicitly listed in 2 trials. Digital pathology AI was featured in 2 trials, and molecular subtyping in 1 trial. Prognostic modeling was included in 1 trial.

The 4 WHO ICTRP registrations were all Chinese trials (ChiCTR). Three of these remain in pending status, and the predominantly observational design across all 9 trials indicates that while prospective data collection is underway, no registered prospective interventional trials evaluating ML-driven clinical decision-making have been initiated. This registry analysis confirms that the prospective translational pipeline for ML in TNBC remains in early-phase observational validation, with no completed or published prospective trials to date.

The original 56:1 translational gap ratio conflated two distinct stages. Stratified analysis reveals a 16:1 retrospective-validation-to-development ratio (24 retrospective studies versus 394 development studies) and a 44:1 prospective-trial-to-development ratio (9 registered trials versus 394 development studies). This indicates that while retrospective clinical validation is increasingly common, the transition to prospective clinical implementation remains limited.

## 4 Discussion

This study conducted a systematic bibliometric analysis of 394 ML-related publications in the field of TNBC from 2011 to 2026, using PubMed to validate their representativeness. The analysis revealed a marked translational gap at the evidence level: while research output has expanded exponentially, the proportion of publications reporting clinical validation or prospective registration remains low (24 retrospective validation studies and 9 registered prospective trials out of 394 computational publications). This metric reflects publication and registration characteristics rather than direct assessment of clinical implementation. Publication volume has surged since 2018, reaching 121 articles in 2025, with publications from the past four years accounting for 61.7% of the total. A global academic landscape has emerged with China and the United States as dual hubs, where China leads in volume while the United States exerts greater influence. Research hotspots are highly concentrated in three major areas: radiomics, multi-omics integration, and analysis of the immune microenvironment. However, our three-tier retrieval strategy identified only 24 retrospective validation studies (6.1%) and 9 registered prospective trials (2.3%); yielding a 16:1 retrospective-validation-to-development ratio and a 44:1 prospective-trial-to-development ratio. This pattern is consistent with a developmental phase in which methodological publications accumulate more rapidly than clinical validation reports. Whether this constitutes a fundamental imbalance requires longitudinal tracking of how the 2019–2022 cohort of retrospective studies progresses toward prospective testing over the next 5–10 years; academic publication shows the expansion of research scale, while patient-benefit-oriented validation appears less frequently. This imbalance is not an isolated phenomenon but a structural dilemma woven from data, methodology, academic evaluation, and geopolitical dynamics, the essence of which is a research ecosystem that prioritizes “the value of papers over clinical value.” To understand this landscape of delayed clinical validation, this section begins by reviewing three-phase publication trends and the insights derived from them. We then analyze the structural characteristics of national, journal, and author collaboration networks. Subsequently, we cross-examine keyword co-occurrence and co-citation clustering to identify three misalignments and deduce future directions. Building on this, we distill the progress and validation gaps across three major technological strands, dissect the causes of translation lag from the dimensions of data standardization, cross-institutional sharing barriers, regulatory fragmentation, the interpretability-trust deficit, and temporal lag. Finally, we will compare our findings with other bibliometric studies, acknowledge methodological limitations, and propose phased, progressive action recommendations and future directions.

### 4.1 Time trends and research progress

The global output of publications in this field has shown an upward trend across three phases: the Embryonic Stage (2011–2018), the Growth Stage (2019–2022), and the Rapid Expansion Stage (2023–2025). This trend coincides with key milestones such as the gradual maturation of machine learning technologies, the growing need for such technologies in cancer diagnosis and treatment, and the introduction of AI healthcare policies. Between 2011 and 2019, thanks to advances in processing power, memory, and storage technologies, as well as the emergence of massive amounts of data, computers were equipped with the ability to handle increasingly complex learning tasks. Although a large number of papers had already explored the application of ML algorithms in medical data at that time, compared to the significant impact of machine learning in other fields, its application in the clinical field was still in the early stages of transitioning into the medical domain (53). The molecular subtyping system for TNBC was only preliminarily established around 2011 (54, 55), providing ML with learnable biological labels; however, public datasets suitable for training ML models had not yet reached a sufficient scale.

The number of publications saw a substantial surge between 2019 and 2023. The growth during this period was driven by improvements in technical infrastructure and the establishment of standardization frameworks: the Imaging Biomarker Standardization Initiative (IBSI) released reference standards in 2020, providing uniform definitions for 174 radiomic features and lowering the technical barriers to cross-study comparisons (56); Between 2020 and 2022, the development guidelines for CONSORT-AI, SPIRIT-AI, and TRIPOD-AI, along with the DECIDE-AI international reporting guidelines, were successively published (57–60), providing transparent reporting standards and evaluation frameworks for the application of ML in clinical research, lowering the barrier to entry for researchers in this field, and laying the foundation for systematic evaluation of algorithm performance and cross-study comparisons. Meanwhile, the TNBC field itself began to mature around 2019, ranging from phenotypic screening to identify therapeutic targets (61), to automated quantitative assessment of tumor-infiltrating lymphocytes (62), and on to precision diagnostics based on nanosensor platforms (63), all have demonstrated the feasibility of ML in various subfields of TNBC and attracted more research teams to verify that ML can uncover patterns in complex TNBC data that traditional statistical methods cannot detect.

The number of publications on ML in the field of TNBC has seen an exponential surge since 2023, a growth trend that closely aligns with the concentrated introduction of AI medical regulatory policies by major global economies between 2021 and 2023. A global systematic review covering the period from 2015 to 2025 shows that regulatory frameworks in the United States, the European Union, and China have become increasingly refined during this time, providing an increasingly clear institutional environment for AI medical research (64). In January 2021, the U.S. FDA released its AI/ML Medical Device Action Plan, which for the first time proposed a tailored regulatory framework for adaptive algorithms (65); in 2023, it issued the Planned Change Control Plan (PCCP) guidance, further refining the approval requirements for iterative and evolving algorithms. In April 2021, the European Commission proposed the Artificial Intelligence Act, classifying medical AI as a high-risk application category and establishing a tiered regulatory system (66). The divergence and convergence of the U.S. and EU regulatory approaches have been the subject of extensive academic discussion in recent years (67). China’s National Medical Products Administration (NMPA) also issued the “Guidance on the Registration Review of Artificial Intelligence Medical Devices” in March 2022; the concentrated issuance of these policies signaled to the academic community that the regulatory path for AI medical products was becoming increasingly clear, providing institutional support for the rapid growth in literature following 2023.

The three-stage evolution described above reveals a pattern: the surge in the volume of published papers is not the result of the natural diffusion of technology, but rather the product of a synergistic interplay among technological maturity, data infrastructure, and regulatory signals. Reporting guidelines around 2019 and regulatory policies from 2021 to 2023 coincided with for the two growth surges, respectively, but both lagged behind technological breakthroughs by two to three years. This implies that if the approval pathway for prospective clinical validation becomes clearer, it could trigger a fourth round of growth; however, if the gap between algorithm development and clinical evidence continues to widen, the current exponential growth may be unsustainable.

### 4.2 Network analysis of countries, journals, and co-authors

According to the National Co-author Network, the global landscape in this field exhibits a dual-core structure: China ranks first in terms of publication volume with 180 papers, while the United States leads in terms of impact with 116 papers and an average of 39.09 citations per paper; this is consistent with high research investment in these two countries relative to other nations. In addition to the aforementioned policy support, the United States’ leading position in this field is also attributable to its comprehensive and authoritative clinical databases, such as SEER, TCGA, and TCIA, which provide the data foundation for training and external validation of ML models; China’s rapid rise in this field aligns with the “New Generation Artificial Intelligence” major project under the “Science and Technology Innovation 2030” initiative, which has designated “human-machine collaborative medical consultations” and “multidisciplinary imaging analysis for cancer diagnosis and treatment” as priority areas since 2021. However, the systematic absence of Africa and Latin America (**Figure 3**) reflects a fundamental imbalance in the global distribution of research resources. The prevalence of TNBC among African American women is nearly three times that of European Americans (68); among women in West Africa, the prevalence reaches as high as 45.7%, making it one of the highest regions globally (69). The distribution of TNBC subtypes among African patients differs significantly from that of European patients, with specific genetic variants being more common in African populations (70), and these genetic characteristics may influence treatment response and prognostic assessment. Hispanic women in both the United States and Latin America are diagnosed with a higher proportion of advanced TNBC, and the 5-year overall survival rate for patients in Latin America is significantly lower than that of their counterparts in the United States (71).These epidemiological characteristics should have spurred more research in this field; however, the systematic exclusion of African and Latin American countries from international collaborative networks, in addition to our literature search being limited to English language sources, means that these clinically significant data have never been incorporated into the training process for ML-TNBC models. This absence stems from a structural imbalance in the global distribution of cancer research resources: clinical trial and research funding is highly concentrated in high-income countries, while low and middle income countries have long been excluded from global research networks. In West Africa, a severe lack of research infrastructure, minimal government funding, and a significant brain drain have left local research heavily reliant on institutions in North America and Europe (72); in Latin America, the number of oncology clinical trials is far lower than in Europe and the United States due to multiple factors, including insufficient government investment, language barriers, and lengthy regulatory approval cycles (73). While the models were trained, validated, and optimized on European and East Asian populations, there has been a lack of systematic evaluation of their predictive accuracy for African and Latin American patients. This bias is not revealed by internal validation because the validation sets are drawn from the same data sources.

In terms of journal analysis, *Journal of Clinical Oncology*, *Lancet Oncology*, *Cancers*, and *Annals of Oncology* form the four core nodes of the co-citation network. *JCO* and *Annals of Oncology* are the flagship journals of ASCO and ESMO, respectively, and publish the most influential clinical trials in the field of TNBC along with their long-term follow-up data. The final overall survival analysis of IMPassion-130 was published in *Annals of Oncology* (74); the ASCENT trial, as the first Phase III study to demonstrate that the ADC sacituzumab govitecan, in previously treated metastatic TNBC, significantly improves progression-free survival and overall survival, had its final analysis report published in *JCO (*75). Endpoint data from these trials, including pCR rates and event-free survival, form the clinical baseline for the training and validation of the ML-TNBC model.

Lancet Oncology’s interdisciplinary focus places it at a pivotal position: it publishes clinical trials as well as methodological explorations, policy analyses, and digital health innovations. At the methodological level, a 2024 study published in *eClinicalMedicine* (part of The Lancet Discovery series) systematically evaluated the interchangeability of 10 AI models for assessing tumor-infiltrating lymphocytes in TNBC. The study found significant differences in analytical validity among the algorithms but high consistency in their prognostic performance; this finding directly addresses a core bottleneck in the TNBC-ML field: whether different ML models can be used interchangeably in clinical settings (41). At the policy and integration level, the 2025 Lucerne Toolbox 3 Consensus Initiative (published in *The Lancet Oncology*) brought together 112 experts from 27 countries and 16 medical associations to identify 15 critical knowledge gaps that AI must address in the comprehensive management of early-stage breast cancer, and proposed 13 actionable clinical trial designs (76). In the realm of digital health translation, the ECTIL study (published in *The Lancet Digital Health*) utilized data from 2,340 breast cancer patients across multiple centers to validate a deep learning TIL assessment model that requires no detailed annotation. This model demonstrated a high correlation with pathologist scores and was independently associated with overall survival; this methodological exploration provides a scalable, evidence-based foundation for standardizing algorithms for immune microenvironment assessment (77). Furthermore, one of the first prospective validation studies on AI-assisted mammography screening (published in *The Lancet Digital Health*) confirmed that when AI replaced a single radiologist in a dual-reader scenario, the cancer detection rate was non-inferior to that of the traditional dual-reader model. This clinical trial marks the beginning of the accumulation of prospective evidence for AI in the field of breast cancer screening (78). Together, these four areas demonstrate the pivotal role of *Lancet Oncology* and its affiliated journals in bridging the three disciplinary frameworks of clinical medicine, computational science, and public health research.

As an MDPI open-access journal, *Cancers* has published a large volume of methodological papers on ML-TNBC since 2020, thanks to its high publication output and rapid review cycle. Its inclusion in the Citation Core reflects researchers’need for rapid publication channels in this field; however, most of these papers remain at the level of algorithm development and retrospective validation, consistent with the scarcity of prospective trials (44:1 prospective-trial-to-development ratio) and the limited proportion of retrospective validation studies (16:1 ratio) identified in this analysis.

In terms of author impact, Ciompi F and Jiang Y are tied for first place in h-index, but their academic impact trajectories differ significantly. Ciompi F has dedicated his research to computational pathology methodology, with a series of studies focusing on the automated quantitative assessment of tumor-infiltrating lymphocytes in TNBC (79), deep learning-based mitosis counting (80), and computer-aided analysis of whole-slide images (62, 81–83). These methodological breakthroughs were primarily published in specialized computational pathology journals such as *Modern Pathology* and *npj Breast Cancer*, with a total of 288 citations across six papers. This reflects a high degree of specialization in his academic trajectory but relatively limited broad-based influence. Jiang Y’s six papers primarily focus on the preoperative identification and prognostic assessment of TNBC using AI-based medical imaging. Representative work includes a 3D U-Net-based DCE-MRI automatic segmentation model that automatically delineates three-dimensional tumor regions from dynamic contrast-enhanced MRI and extracts 15 radiomic features. A support vector machine (SVM) was used to construct a classification model, achieving an AUC of 0.867 (84). These studies have been published in clinical oncology journals such as *Computational and Mathematical Methods in Medicine* and have been cited a total of 107 times. While Ciompi F’s research addresses the standardization of quantitative assessment using histological sections, and Jiang Y’s work tackles the clinical challenge of preoperative non-invasive identification using imaging, both types of research despite their differing approaches face a translational gap between algorithm development and prospective validation. Chen C topped the citation rankings with a total of 751 citations for a single review article (25). The paper systematically reviewed TNBC molecular subtyping systems based on the immunogenomic profile and proposed a classification framework comprising three subtypes: high, intermediate, and low immune activity. This conceptual framework provided a theoretical foundation for training labels and feature engineering in subsequent machine learning models.

In the analysis of author collaborations, all five clusters were dominated by Chinese authors; this pattern may be related to the incentive mechanisms of the domestic academic evaluation system. Currently, talent evaluation, professional title promotion, and disciplinary assessments in universities and hospitals rely heavily on the number of publications and impact factors, which may contribute to researchers prioritizing rapid output within their existing collaboration networks. Interviews with researchers also reveal that Chinese medical researchers generally adopt a cautious attitude toward data sharing, with security concerns and ethical uncertainties serving as the primary barriers (85).

### 4.3 Keywords, co-citation analysis, and research trends

The keyword co-occurrence network reflects the terms currently used by researchers, while the co-citation clustering reveals which documents form the foundation of knowledge. By examining the keyword co-occurrence network (**Figure 7**) and the co-citation clustering (**Figure 12**) within the same temporal framework, we can identify three discrepancies that would be difficult to detect by analyzing either graph individually.

First, the emergence of methodological trends preceded the establishment of a solid foundation for validation. The keyword timeline shows that radiomics (Cluster #7, LLR = 18.50) and drug repositioning (Cluster #6, LLR = 17.52) surged between 2020 and 2022, indicating that researchers are intensively pursuing these directions. However, although the corresponding knowledge bases in the co-citation clusters (Cluster #3, mean Year=2019; Cluster #0, mean S1Year=2021) are substantial in scale, they do not contain validation-related terms such as “clinical trial” or “prospective validation.” Only 9 registered trials and 24 retrospective validation studies were identified; all 24 peer-reviewed validation studies were retrospective in design, while the 9 registered trials were prospective observational or diagnostic studies with no interventional component, corroborating the assessment that a large number of new studies are continuing to build upon a knowledge base that has not undergone prospective validation.

Second, there is a disconnect between emerging technologies and mainstream knowledge frameworks. Cluster #12 in the CiteSpace timeline (Raman imaging, with only 6 papers, meanYear=2026, Silhouette=1.0) represents the latest methodological advances, but it is nearly invisible in the VOSviewer keyword co-occurrence network due to its small nodes, failing to form effective co-occurrence connections with deep learning (purple cluster) and radiomics (yellow cluster). Furthermore, no corresponding knowledge module has formed in the co-citation network. This indicates that the validation pathway for this research direction from functional metabolic assessment to prospective patient cohorts remains unclear (86, 87).

Third, the abundance of omics data coexists with insufficient algorithmic integration. The keyword emergence table shows that “tumor-infiltrating lymphocytes” (intensity 2.17) and “gene expression” (1.74) were the terms with the highest emergence intensity from 2023 to 2026, reflecting that the analysis of the immune microenvironment is currently the most active research focus. However, the connection strength between the light blue cluster (biomarkers, gene signature, immune infiltration) and the deep learning cluster (purple) in the keyword co-occurrence network is relatively weak; similarly, in the co-citation cluster, there is a lack of effective bridges between TLS and the single-cell cluster (#4) as well as the AI imaging clusters (#1/#3). This indicates that omics discoveries have not yet been systematically integrated into the knowledge workflow of ML predictive models, and a closed-loop has not been established among transcriptomic biomarkers, protein expression validated by immunohistochemistry, and imaging features (88, 89). Although previous studies have attempted to integrate multimodal data to construct predictors of treatment response, such cross-modal efforts remain rare in TNBC (90, 91).

These three discrepancies collectively point to a core conclusion: while methodological advancements in this field continue to accelerate, the validation process has not kept pace. Based on this assessment, future research should focus on achieving breakthroughs in three key areas: first, advancing from retrospective validation to prospective efficacy trials for radiomics and deep learning models, particularly in the context of neoadjuvant treatment decision-making; Second, achieving deep integration of multi-omics data with AI algorithms to drive models from “validating known biomarkers” toward “autonomously discovering new regulatory networks”; and third, advancing AI-screened molecular candidates into early-phase clinical trials to bridge the translational gap from *in vitro* to *in vivo* studies.

### 4.4 Clinical interpretation and validation landscape

To move beyond bibliometric co-occurrence frequencies and address the translational gap from algorithm development to clinical implementation, we interpreted the ML-TNBC research outputs from three complementary perspectives: citation-based translational readiness, institutional research origin, and scenario-specific clinical utility.

Citation impact versus clinical validation maturity. The clinical appraisal of the 10 most-cited papers (**Table 6**) reveals a marked disconnect between bibliometric visibility and clinical validation strength. Three were narrative reviews without primary patient data (7, 20, 21). Three were basic science studies that employed computational methods for image segmentation or mechanism analysis but did not evaluate patient-level clinical prediction endpoints (22–24). Only four were original studies that developed patient-level predictive models, all of which relied on retrospective cohorts or public databases (TCGA) with internal validation. None reported independent multi-center external validation or prospective interventional testing. This pattern indicates that the most frequently cited papers in this field are not those closest to clinical implementation. They are instead the papers that introduced novel biological concepts or methodological frameworks. The four patient-level predictive modeling studies among the Top 10 all relied on public databases or single-center retrospective cohorts, with none progressing to multi-center validation or prospective trial evaluation. This pattern is consistent with the broader translation bottleneck identified in our registry analysis (Section 3.8), and underscores a fundamental imbalance: the most visible research outputs are not those closest to clinical implementation, but those that are most accessible to produce within a computational framework. The observed decoupling is consistent with a field that may reward methodological novelty and database-scale analyses over evidence suitable for clinical deployment. Future efforts should prioritize independent external validation and prospective cohort studies to generate evidence that can genuinely inform clinical decision-making in TNBC management.

Research origins and translational orientation. Analysis of corresponding author affiliations across 393 studies with identifiable addresses demonstrates that the translational gap is not attributable to a lack of clinical involvement. Of these, 233 (59.3%) originated from clinical or medical affiliated institutions, including hospitals, cancer centers, clinical departments, and medical universities. The proportion of clinically affiliated institutions increased from 4 studies in 2018 to 83 in 2025, indicating growing participation of clinical centers. The most productive institutions, including Fudan University (11 studies), Sichuan University (8 studies), Fujian Medical University (7 studies), and Shanghai Jiao Tong University (5 studies), were all classified as clinical or medical affiliated. Only 2 of the top 15 most productive institutions were classified as academic or research (Sun Yat-sen University, 4 studies) or engineering or technical (University of Electronic Science and Technology of China, 3 studies). This institutional distribution reveals that clinical and medical institutions are the dominant contributors to ML-TNBC literature. Even these clinically affiliated outputs, however, remain predominantly retrospective proof-of-concept studies rather than prospective clinical validations. The bottleneck lies not in who is conducting the research, but in what type of evidence is being produced. A review of the first and corresponding author affiliations among the 10 most-cited papers reveals that studies originating from university medical departments or cancer centers were more likely to incorporate patient-level cohort data, whereas those led by pure computational or engineering departments predominantly relied on public repositories or cell line data. This pattern, observed in the highest-citation tier, suggests an institutional divergence in translational orientation: clinically affiliated research tends to produce patient-centered data, yet even these outputs rarely progress beyond retrospective proof-of-concept.

Scenario-specific clinical utility and limitations. The four major ML application domains identified in this study exhibit markedly different readiness for clinical deployment. For radiomics and imaging-based diagnosis, ML models have achieved high retrospective AUC values for molecular subtyping and lesion characterization. The clinical value of radiomics in TNBC lies in its potential to provide non-invasive, low-cost, and repeatable assessments of tumor heterogeneity, which could supplement conventional BI-RADS classification for indeterminate lesions. The clinical deployment of these models is constrained by scanner parameter heterogeneity (MRI field strength, sequence protocols, reconstruction kernels), feature instability across vendors, and the lack of prospective interventional trials demonstrating improved diagnostic accuracy or altered management decisions beyond conventional radiologist assessment. Until multi-center prospective trials validate that radiomics-guided biopsy decisions reduce unnecessary procedures or improve cancer detection rates, this application remains at the proof-of-concept stage.

For neoadjuvant chemotherapy response prediction, pathological complete response (pCR) is the standard endpoint for neoadjuvant chemotherapy efficacy in TNBC and is strongly associated with long-term event-free survival and overall survival. ML models are widely developed to predict pCR, yet the overwhelming majority rely on single-center retrospective data. The clinical utility of ML-predicted pCR lies in its potential to identify patients who may benefit from treatment escalation (additional platinum agents or immunotherapy) or de-escalation (omitting surgery for exceptional responders). pCR definitions vary across studies (Miller-Payne grade 5 versus Residual Cancer Burden index 0), and current ML models predict pCR as a *post-hoc* comparison rather than a utility trial that prospectively informs treatment modification. The KEYNOTE-522 trial established that pCR improvement translates to event-free survival benefit with pembrolizumab, but no prospective trial has demonstrated that ML-predicted pCR status can safely guide treatment decisions. Retrospective models are susceptible to time-dependent evaluation bias (only patients who complete NAC are included), and the surrogate endpoint validity of pCR for overall survival in ML-selected subgroups remains unproven.

For digital pathology and tumor-infiltrating lymphocyte assessment, ML-assisted quantification offers objectivity advantages over manual evaluation. Tumor-infiltrating lymphocytes (TILs) have been incorporated into ESMO and St. Gallen consensus guidelines as a prognostic biomarker in TNBC (92), and the International Immuno-Oncology Biomarkers Working Group has established standardized visual assessment criteria (93). The clinical utility of ML-based TIL quantification is its potential to replace labor-intensive manual scoring with objective, reproducible, and spatially resolved assessment. Automated TIL assessment could standardize pathology reporting and potentially stratify patients for immunotherapy eligibility. The clinical importance of immune biomarker assessment in TNBC has been further underscored by phase 3 trials demonstrating that PD-L1 positivity, which correlates with TILs density, predicts benefit from immune checkpoint inhibitors in both advanced and early-stage settings (94, 95). However, ML-based TIL assessment relies on retrospective training on manually annotated regions of interest. Inter-laboratory staining variability, whole-slide scanner differences, and the absence of internationally validated algorithmic thresholds preclude its incorporation into routine pathology reporting (62).Recent multicenter work has demonstrated the feasibility of scalable computational TIL assessment across 2340 breast cancer patients in a multi-institutional setting (77). Extension of such frameworks to TNBC-specific cohorts would directly address the current 16:1 retrospective-validation gap in this domain. Nevertheless, the clinical validation of digital pathology TIL quantification requires not only algorithmic accuracy but also demonstration that ML-guided TIL assessment changes adjuvant therapy recommendations compared to standard pathologist evaluation.

For multi-omics and immune microenvironment-based prognostic models, multi-omics integration theoretically addresses TNBC molecular heterogeneity by combining genomic, transcriptomic, and proteomic data to discover novel therapeutic targets and refine prognostic stratification. The clinical value is the potential to move beyond anatomical staging toward molecular subtype-guided therapy selection. Numerous gene signatures have been identified, yet their clinical applicability is limited by dependence on transcriptomic profiling, which is not routinely available in most clinical settings, and by the lack of prospective validation in independent real-world cohorts. The clinical operability of multi-omics models is further constrained by platform accessibility, sequencing cost, batch effects, and the absence of standardized bioinformatic pipelines. The majority of multi-omics ML studies rely on retrospective public database cohorts (TCGA, GEO, METABRIC) with batch-confounded training and validation. Hayes and colleagues established criteria for clinical utility of tumor markers, which require not only analytical validity but also prospective demonstration of clinical validity and utility. No multi-omics ML model in TNBC has met these criteria. The weak co-occurrence connection between the biomarker cluster and the deep learning cluster in the keyword network structurally reflects this disconnect: omics discoveries have not been systematically integrated into mainstream ML-driven imaging workflows.

Taken together, these layered interpretations demonstrate that while ML research in TNBC has achieved robust retrospective validation across diverse clinical scenarios, the field has not yet transitioned to prospective clinical implementation. The translational bottleneck is not a shortage of clinical research participation or algorithmic performance in retrospective settings, but a systemic absence of prospective interventional trials, independent multi-center external validation, and clinically actionable endpoints that satisfy the evidentiary standards for clinical deployment. Addressing this gap will require deliberate prioritization of clinically anchored research questions, prospective trial designs, and multi-center validation frameworks that evaluate real-world patient outcomes rather than retrospective predictive accuracy alone.

### 4.5 Technological advances and validation gaps in research hotspots

Based on the results of keyword co-occurrence, co-citation clustering, and bibliometric analysis, three distinct technical approaches to machine learning have emerged in the field of TNBC. While each has progressed to a different stage, they all face similar validation challenges.

Radiomics and deep learning constitute the most mature technological approaches in this field. From screening (mammography CAD) to diagnosis (MRI/ultrasound radiomics) to predicting the efficacy of neoadjuvant therapy (pCR), a technology stack has been established that covers the entire clinical continuum. The close interweaving of keywords in the purple cluster (deep learning, CNN) and the yellow cluster (MRI, radiomics, features, pCR), along with the knowledge support from co-cited clusters #3 (radiomics-based ML model, meanYear=2019) and #10 (early detection, meanYear=2022), confirms the maturity of this direction from the perspective of the graph structure. Furthermore, its retrospective performance metrics are also excellent: ultrasound radiomics has been shown to improve the prognostic predictive ability of TNBC clinical and pathological features (96, 97), and when combined with ML models, it performs well in predicting overall survival and disease-free survival (74); AUC values for pCR prediction models typically range from 0.80 to 0.95, and multicenter studies such as BSTNet have achieved external validation AUCs exceeding 0.85 across three independent centers (98, 99). However, the validation gap in this technical line is particularly striking: all 9 registered trials were non-interventional in design, with no prospective randomized controlled trials; Although screening-level AI models demonstrate excellent AUC performance in retrospective data (100, 101), they have not been shown to reduce the incidence of interstitial cancer or the proportion of advanced breast cancer in prospective population trials (102); the endpoint for pCR prediction remains “*post-hoc* comparison” rather than a utility trial (103); In the external validation of Chemo-prAIdict for TNBC, the AUC plummeted to 0.677 (104), suggesting that subtype generalization remains an unsolved challenge. Furthermore, systematic reviews reveal that feature extraction and validation methods vary greatly within the field, with AUCs ranging from 0.64 to 1.00 and a widespread lack of calibration curves (105, 106).

Multi-omics integration and analysis of the immune microenvironment represent cutting-edge technological advancements. The keyword “red cluster” delves from bulk tissue typing to single-cell, RNA-Seq, TILs, and PD-L1. Citation clusters #4 (tertiary lymphoid structure, single-cell analysis, meanYear=2021) and #7 (computational TIL assessment, meanYear=2017) provide the underlying knowledge foundation. Technological advancements are remarkable: single-cell transcriptomics can now deconstruct the evolutionary trajectories of TNBC-resistant clones under chemotherapy stress on a cell-by-cell basis (107); differential response patterns following immunotherapy combined with radiotherapy have been precisely characterized using single-cell and spatial mapping (108); TLS assessment has advanced from binary classification to refined quantification of spatial distribution, maturity, and topological structure (109, 110); XGBoost-based ML models can predict TLS spatial distribution and response to neoadjuvant therapy from MRI features (111), and the integration of multimodal information has even clarified the role of antigen-presenting mast cells in anti-PD-1 efficacy, leading to the initiation of clinical trials (112). However, the isolated presence of light blue keyword clusters (biomarkers, gene signature, immune infiltration) within the network reveals a structural bottleneck: an effective knowledge loop has not been established among the three components: gene biomarkers identified through transcriptomics, protein expression validated by immunohistochemistry, and features extracted from radiomics. The application of ML models in this field has largely focused on “predicting known biomarkers” rather than “autonomously discovering new mechanisms from massive datasets.” Although previous studies have attempted to integrate digital pathology, RNA sequencing, and DNA sequencing data to construct predictors of treatment response (88), or proposed analytical frameworks for multimodal data integration (89), such cross-modal integration remains rare in TNBC, with most studies still limited to a single omics modality or a single imaging modality (90, 91).

Drug repurposing and functional spectroscopy have opened up the latest methodological avenues. The keyword timeline cluster #6, “drug repurposing” (meanYear=2021, LLR = 17.52, LSI pointing to WEE1 kinase), marks the emergence of algorithm-driven drug repurposing as an independent research direction; reports have already appeared on ML models predicting the affinity of small molecules for kinase pockets and screening for lead compounds (111). By the 2023–2026 frontier period, Cluster #1, drug delivery (meanYear=2024), focuses on the intersection of smart nanodelivery systems and the regulation of mitochondrial autophagy (112), while Cluster #12, Raman imaging (meanYear=2026, Silhouette=1.0), represents label-free metabolic phenotyping technologies based on Raman spectroscopy, used to elucidate drug resistance heterogeneity in TNBC (86, 87). However, the validation gap in this technology line is the most fundamental: from virtual screening to nanodelivery to Raman metabolic phenotyping, the entire chain is almost entirely confined within the closed loop of in silico → *in vitro* → preclinical. Although preclinical models and translation (biology) appear in the zoomed-in view of **Figure 9**, terms such as Phase I and clinical trials are systematically absent. Validation of WEE1 inhibitors has been limited to proliferation inhibition in TNBC cell lines, while ML-enhanced nanoparticle design has been confined to *in vitro* cellular uptake experiments (113). A comprehensive assessment of AI-driven drug discovery reveals that a significant proportion of studies remain in the preclinical stage; the very few cases that have advanced to early clinical trials primarily target pan-cancer targets rather than TNBC-specific indications (114, 115). Although Raman spectroscopy offers unique advantages in terms of single-cell resolution, and there have been a few imaging studies involving retrospective FFPE pathological specimens (116), it lacks systematic validation in prospective patient cohorts. Integration with existing deep learning frameworks has not yet been established, and research remains entirely confined to *in vitro* cellular experiments.

Behind this three-pronged technological approach lies a common bottleneck in translation: whether it be imaging diagnosis and pCR prediction, analysis of the immune microenvironment and biomarker discovery, or virtual screening and metabolic phenotype assessment, nearly all efforts have stalled at the retrospective validation or *in vitro* experimentation stage. The systematic absence of terms such as “prospective clinical trials” and “multicenter external validation” across the entire knowledge graph indicates that while the field is thriving in areas such as algorithms, imaging, and drug delivery, the accumulation of knowledge regarding clinical validation has not yet entered the core structure of the network. This disconnect between technology and clinical practice serves as the starting point for the structural analysis presented below.

### 4.6 Possible causes of delays in clinical translation

The marked translational gap identified in this study may be related to the following reasons. The original 56:1 ratio conflated two distinct translational stages. The revised stratified analysis reveals a 16:1 retrospective-validation-to-development ratio (24 retrospective studies versus 394 development studies) and a 44:1 prospective-trial-to-development ratio (9 registered trials versus 394 development studies). Both ratios indicate substantial delays in clinical translation, but the underlying causes differ by stage.

First, multi-center data standardization and clinical endpoint harmonization remain unresolved (117). Our keyword co-occurrence and co-citation analyses revealed that although radiomics and deep learning models have achieved high retrospective AUC values, the heterogeneity of imaging protocols, scanner manufacturers, and reconstruction kernels across institutions may limit model generalizability (56, 118). The Image Biomarker Standardization Initiative (IBSI) has provided standardized definitions for 174 radiomic features, yet compliance remains limited in published studies. For pCR prediction, the absence of a unified endpoint definition (Miller-Payne grade 5 versus Residual Cancer Burden index 0) means that models trained on one standard cannot be directly validated against another (119). The lack of calibrated reporting standards and the absence of prospective utility trials further mean that even technically sound models lack the evidentiary foundation required for clinical adoption. These gaps in data standardization and endpoint definition may create barriers to multi-center validation and regulatory approval, contributing to the scarcity of prospective trials that would satisfy the 44:1 ratio.

Second, barriers to cross-institutional data sharing create structural constraints on external validation (120). Our co-authorship network analysis revealed that Chinese institutions form tightly clustered domestic collaboration networks, while inter-institutional data sharing within the United States is also limited by independent bioinformatics infrastructure at major cancer centers. Cross-border data transfer faces additional ethical review and de-identification requirements (85). When training data are confined to single institutions or narrow geographic regions, algorithms trained on homogeneous data may learn population-specific and protocol-specific patterns rather than broadly generalizable disease biology. This homogeneity in training data is one factor that may contribute to the drops in external validation AUCs frequently reported in the literature, and why multi-center prospective trials remain scarce.

Third, the regulatory pathway for AI-based medical devices in oncology remains incomplete and regionally fragmented. The FDA AI/ML Medical Device Action Plan (2021) and the Planned Change Control Plan (PCCP) guidance (2023) have clarified approval requirements for adaptive algorithms in the United States. The European Commission Artificial Intelligence Act (2021) classifies medical AI as high-risk. China’s NMPA issued guidance on AI medical device registration in 2022. However, these frameworks have not yet converged on a unified approach to validating ML models for cancer diagnosis and treatment response prediction. No ML-TNBC model has obtained FDA approval or NMPA class III certification for clinical deployment. Regulatory considerations and validation protocols designed to meet regulatory evidentiary standards were not prominent in the titles, abstracts, and keywords of the 394 computational studies in our analysis. The absence of clear regulatory endpoints for clinical utility trials (as distinct from analytical validation trials) means that researchers lack guidance on what evidence standards are required for approval. This regulatory uncertainty has been cited in the literature as a potential barrier to investment in prospective clinical validation. The observed 44:1 prospective-trial deficit is consistent with this hypothesis, although direct causal attribution cannot be established from bibliometric data alone.

Fourth, the current development of explainable artificial intelligence (XAI) faces a dual misalignment. One is a temporal misalignment: explainability is often added as a *post-hoc* measure after the algorithm is completed, rather than being embedded into the architecture from the very beginning of model design (121). Second, a subject-oriented misalignment: clinicians are rarely involved in the evaluation and iteration of explanation tools, leading to continuous optimization of technical metrics (accuracy, precision) while the core question of whether the explanation is sufficient to support clinical decision-making is sidelined (122, 123). Even though methods such as LIME, SHAP, and Grad-CAM have been widely adopted, the explanations they generate are technically coherent but fail to address the core issues of clinical trust and decision accountability. Meanwhile, while XAI can help clinicians understand the primary factors influencing model predictions, the black-box nature of these models still undermines clinicians’ confidence and the effectiveness of their communication with patients (122). This has led current XAI tools to focus their research on why a model makes a particular prediction, rather than on the extent to which that prediction should alter clinical treatment decisions. The black-box nature of models, combined with the limited clinical integration of explanatory tools, may contribute to a trust deficit that affects clinician acceptance. This trust deficit affects both the 16:1 retrospective validation gap (clinicians are reluctant to adopt even externally validated models without interpretable explanations) and the 44:1 prospective trial gap (regulators and ethics committees require transparent decision logic before approving interventional trials). Recent work has begun to demonstrate that XAI frameworks can be integrated directly into TNBC therapeutic discovery pipelines. Explainable models for molecular classification and inhibitor screening have shown that feature attribution methods can identify biologically actionable targets rather than merely explaining predictions (123, 124). These studies suggest that XAI may eventually bridge the gap between black-box prediction and mechanistic insight, though they remain at the preclinical validation stage.

Finally, the 44:1 prospective trial ratio may be somewhat overestimated due to time lags. Research on machine learning in the TNBC field only entered a period of rapid growth starting in 2019 (**Figure 2**), whereas a prospective randomized controlled trial typically takes 5 to 7 years or even longer to complete, from design and registration to subject enrollment, follow-up, and publication of results. In contrast, the publication cycle for retrospective algorithm-based papers is much shorter. The 16:1 retrospective validation ratio is less affected by time lags, as retrospective studies can be completed more quickly. Therefore, the prospective gap currently observed may partly reflect a natural time lag inherent in emerging fields, where algorithmic exploration precedes clinical validation. It is anticipated that as the retrospective studies published between 2019 and 2022 are gradually translated into prospective trials, the 44:1 ratio is expected to decrease over the next 5 to 10 years, while the 16:1 retrospective validation ratio may remain relatively stable as retrospective external validation becomes more routine.

### 4.7 Recommendations for addressing delays in clinical translation and future prospects

Based on the aforementioned shortcomings and the key issues identified across the three levels, future actions can be divided into several phases based on urgency, with each phase corresponding to specific clinical benefits.

To address the systemic translation barriers ranging from data homogeneity to validation gaps, this study proposes a phased, progressive action framework to bridge the gap between computational outputs and clinical evidence. In the near term (1–2 years), priority should be given to utilizing public data platforms such as TCIA and TCGA to initiate multicenter external validation of retrospective models that have received high citations and are open-source. For example, the 2018 radiomics model developed by Saha A et al. based on 922 breast DCE-MRI images (29)or the 2020 IBSI radiomics standardization initiative by Zwanenburg A (56)can be used to obtain evidence of cross-institutional generalizability at minimal cost. Concurrently, reporting standards such as TRIPOD-AI and MI-CLAIM must be elevated from submission recommendations to mandatory journal requirements, thereby rapidly narrowing the gap between method development and reporting quality by enhancing methodological reproducibility. In the medium term (2–5 years), we need to adopt the governance models of TCIA/TCGA to implement a federated learning framework, enabling cross-continental, multi-center model training and validation without transferring raw data, thereby addressing the systemic exclusion of African and Latin American countries from national collaborative networks (**Figure 3**). In the long term (5–10 years), randomized controlled trials comparing AI-assisted decision-making with conventional decision-making, centered on patient endpoints (such as pCR rates and event-free survival), must be designed. Concurrently, efforts should be made to explore embedding AI models as secondary or exploratory endpoints within existing TNBC drug clinical trial frameworks. Establishing a foundation of trust through short-term external validation, expanding geographic and population coverage via mid-term data consortia, and confirming clinical utility through long-term prospective trials may help reduce the current 16:1 retrospective-validation gap and 44:1 prospective-trial gap and shift machine learning research in the TNBC field from being method-driven to truly clinical-driven.

These three emerging research areas should also focus on different clinical endpoints. In the field of radiomics, the immediate goal is to conduct prospective pCR efficacy trials to demonstrate that AI predictions can influence treatment decisions; for the multi-omics approach, the near-term goal is to break down the barriers between imaging, pathology, and omics data, enabling models to evolve from predicting known biomarkers to discovering new mechanisms, ultimately yielding biomarker combinations that can guide immunotherapy stratification; and for the drug and spectroscopy approach, the near-term goal is to advance AI-screened lead compounds and Raman metabolic phenotypic features into Phase I or II clinical trials to definitively determine their efficacy in humans.

If these three tiers of action can be advanced step by step, the role of machine learning in the field of TNBC will evolve from its current status as an auxiliary diagnostic tool to become the infrastructure for precision treatment decisions. Imagingomics will become the standard assessment method before and after neoadjuvant therapy, multi-omics integration will generate dynamically evolving molecular profiles for each patient, and AI-driven drug screening will shorten the timeline from target discovery to clinical validation. However, this vision hinges on validation keeping pace; Closing this gap requires that clinical validation efforts keep pace with methodological publication output.

Beyond these immediate priorities, emerging technological directions warrant attention. Foundation models trained on spatial proteomics data are beginning to demonstrate zero-shot biomarker discovery in TNBC, with initial evidence of treatment response prediction outperforming established clinical stratification (125) Multimodal architectures that integrate imaging, pathology, and molecular data within unified frameworks are also advancing rapidly (126). These approaches remain retrospective and preclinical, but they represent the most likely pathways through which the current evidence gap may begin to narrow.

## 5 A comparison with previous bibliometric studies

Previous bibliometric studies have largely focused on the overall intersection of breast cancer and artificial intelligence, but have rarely addressed triple-negative breast cancer, a highly heterogeneous subtype. An analysis by Wu et al. of 2,701 articles from the Web of Science Core Collection revealed that the number of publications on AI and radiomics in the field of breast cancer imaging has grown rapidly since 2018. China and the United States rank first in terms of publication volume and citation count, respectively, while “explainable AI,” “neoadjuvant chemotherapy,” and “lymphovascular invasion” have emerged as prominent research trends in recent years (127). Another bibliometric analysis based on 1,537 articles from the Web of Science database similarly confirmed a surge in research after 2019, noting that “deep learning,” “radiomics,” and “transfer learning” have become consistently active keywords (128). The above macro-level bibliometric studies provide important insights into the overall development landscape of AI in the field of breast cancer; however, since their analysis encompasses all breast cancer subtypes, they struggle to capture the unique knowledge structure of TNBC—the most aggressive subtype and the one with the most intensive application of machine learning.

Regarding specialized metagenomic research on TNBC, existing studies have largely focused on specific technical areas or clinical stages rather than the systematic application of machine learning as a cross-disciplinary method. Wang et al.’s metagenomic analysis of TNBC biomarkers identified six major research hotspots, including immune checkpoint markers, microenvironment-related markers, and circulating tumor DNA, and prospectively noted that “the application of machine learning and artificial intelligence” represents a key future direction for the field (129). Yang et al. systematically reviewed the literature on TNBC neoadjuvant therapy from 2002 to 2023, finding that the research focus is gradually shifting from molecular biology and genetics toward immunology and clinical research (130). Additionally, Li et al.’s omics analysis of antibody-drug conjugates in TNBC focused on clinical translation progress for specific drug classes, while omics studies on immune checkpoints and immunotherapy were similarly limited to single-mechanism pathways (131). While the aforementioned bibliometric analyses on TNBC each have their own specific focus, none of them treat machine learning as a core analytical object, nor do they provide a quantitative assessment of the translation chain from algorithm development to clinical validation. Recent comprehensive reviews have begun to address this gap by documenting the dominance of radiomics and deep learning across the full continuum (122). Our bibliometric analysis builds on these qualitative observations by providing quantitative evidence for this pattern and extending it through the first quantification of the clinical translation gap.

Compared with the aforementioned studies, the innovation of this study lies in three aspects. First, by focusing on the TNBC subtype and using ML as the sole methodological approach, this study bridges the gap between previous macro-level bibliometric analyses of breast cancer and specialized bibliometric analyses of TNBC that have focused on specific areas such as biomarkers and neoadjuvant therapy. Second, it quantifies the clinical translation gap in this field for the first time: among 394 ML computational studies, 24 retrospective validation studies (6.1%) and 9 registered prospective trials (2.3%) were identified, yielding a 16:1 retrospective-validation-to-development ratio and a 44:1 prospective-trial-to-development ratio. No prospective interventional trials with published results were found. This finding represents a direction that has not been primarily addressed in any previous metaanalytic work. Third, the study employed an analytical strategy combining the merger of two databases with independent database validation. This approach integrated Web of Science and Scopus to enhance literature coverage while using PubMed for independent validation to confirm the robustness of core conclusions. These features enable the study not only to present a comprehensive panorama of ML research in the TNBC field but also to reveal the structural disconnect between algorithmic proliferation and clinical implementation, thereby providing quantifiable, evidence-based grounds for subsequent research to shift from methodological exploration toward clinical application.

## 6 Limitations

This study has several limitations. First, the search strategy was limited to the Web of Science Core Collection (WoSCC) and Scopus databases, as described in Section 2.1, and validated using PubMed for clinical trial identification. Relevant studies published exclusively in Embase, IEEE Xplore, arXiv, or MEDLINE may have been missed. Specifically, this analysis likely excluded studies published in MICCAI, CVPR, IEEE ISBI, and other engineering-oriented conferences, where numerous technical innovations in medical imaging AI have been reported. This gap in coverage could further inflate the calculated number of computational studies, thereby making the relative proportion of the translation deficit appear even larger than it actually is. Second, the quantification of the clinical translation gap relies on PubMed document type filtering (“Clinical Trial” or “Randomized Controlled Trial”). While this search strategy has high specificity, it may omit some studies that do not explicitly include these terms but actually contain elements of prospective validation, such as papers employing “prospective cohort,” “validation cohort,” or “clinical utility” designs that are not classified as clinical trials in the document type field. Therefore, the 44:1 prospective-trial-to-development ratio primarily reflects the gap in strictly defined clinical trials rather than the full scope of all prospective work. The 16:1 retrospective-validation-to-development ratio is less affected by this classification bias, as retrospective validation studies are more consistently indexed. Third, high citation counts do not necessarily indicate high quality or clinical relevance. Some highly cited review or methodological papers may mask a large number of studies with low levels of validation. Considering that the gap between computational research and clinical validation may be partly due to insufficient citation accumulation for prospective validation studies, the 44:1 ratio may be moderately reduced as these studies accumulate citations over time. The 16:1 ratio is less affected by citation lag, as retrospective validation studies are published more quickly and accumulate citations sooner. Fourth, citation accumulation is subject to temporal bias. The citation metrics in this study reflect the current bibliometric landscape as of January 2026, and the 2020–2023 COVID-19 pandemic may have affected the publication volume and citation dynamics of cancer AI research. Papers published earlier in the analysis period have had more time to accumulate citations than those published in 2024-2025, creating a publication year bias that favors older publications. This temporal bias is particularly relevant for the rapidly evolving AI literature, where methodological innovations in the most recent 2–3 years may have high scientific impact but low current citation counts. Fifth, this study only included English-language publications, and important contributions published in Chinese or other languages may have been overlooked. This may impact analyses of international collaboration and institutional rankings and, to some extent, limits the cross-linguistic generalizability of the study’s conclusions. Sixth, the study protocol of this bibliometric analysis was not registered in the PROSPERO database, which may have introduced a certain degree of subjectivity in the analysis design and conclusions. Finally, the retrospective cross-sectional design of this study means that we were unable to infer causal relationships between research characteristics and citation patterns.

## 7 Conclusion

This study conducted a systematic bibliometric analysis of 394 core publications from 2011 to 2026. The results indicate that research on machine learning in the field of triple-negative breast cancer has evolved from an early exploratory phase into a high-output research area. The number of publications has entered a phase of exponential growth since 2023, reaching 121 in a single year by 2025. The global research landscape exhibits a dual-core structure centered on China and the United States: China leads with 180 publications, while the United States ranks first in terms of impact, with an average of 39.09 citations per paper. Three major technical approaches have emerged: radiomics and deep learning, multi-omics biomarker discovery, and treatment response prediction. However, the rapid expansion of technical output stands in stark contrast to a severe lag in clinical validation: our three-tier retrieval strategy identified only 24 retrospective validation studies (6.1%) through expanded PubMed screening and 9 registered prospective trials (2.3%) through ClinicalTrials.gov and WHO ICTRP searches, relative to 394 computational development studies, yielding a 16:1 retrospective-validation-to-development ratio and a 44:1 prospective-trial-to-development ratio. No prospective interventional trials with published results were identified. Independent validation via PubMed ruled out potential database bias; publication trends across the two datasets were highly consistent (r = 0.989, p < 0.001), and core keywords showed high overlap (Jaccard = 0.875), indicating that this translation deficit represents a genuine structural issue rather than an artifact of search strategy.

The causes of this translation lag span multiple dimensions. Data standardization and clinical endpoint harmonization remain unresolved across institutions. Cross-institutional data sharing faces structural and ethical barriers. Regulatory pathways for AI-based medical devices in oncology are incomplete and regionally fragmented. The current development of explainable artificial intelligence suffers from a dual misalignment between technical optimization and clinical decision support. Finally, the natural 5–7 year cycle of prospective trials contributes to a temporal lag, though this alone cannot explain the magnitude of the gap.

To break this deadlock, a phased approach is required. In the short term, public data platforms should be utilized to conduct multicenter external validation of highly cited published models, while simultaneously advancing AI model reporting standards from recommendations to mandatory requirements. In the medium term, federated learning should be employed to enable cross-continental collaboration without transferring raw data, thereby correcting geographical bias at the institutional level. In the long term, AI endpoints should be integrated into existing TNBC drug clinical trials, and the threshold for prospective validation should be lowered. Only when the pace of algorithm development truly aligns with the depth of clinical validation, and when clinicians are empowered as active partners in AI implementation rather than passive recipients can machine learning transition from a technical showcase on retrospective datasets to an effective tool for improving actual outcomes for patients with triple-negative breast cancer.

## Data Availability

The original contributions presented in the study are included in the article/**Supplementary Material**. Further inquiries can be directed to the corresponding author.

## Supplementary material

Supplementary Figure 1 LOESS fit sensitivity to span parameter. Alternative span values (0.30, 0.35, 0.45, 0.50) are compared against the selected span of 0.40 (red solid line). The S-shaped growth trajectory is preserved across all candidate spans, with predicted 2025 publication counts varying by less than 2.1%.
