# Supplementary material for: The translational chasm in machine learning for triple-negative breast cancer: a quantitative landscape assessment
Source: Front Oncol. 2026 Sep 24;16:1912317. doi: 10.3389/fonc.2026.1912317 (PMC13645570; doi:10.3389/fonc.2026.1912317)
Supplement: Supplementary file 1 [file SupplementaryFile1.docx]

Supplementary Methods

1. Software Environment

Python: version 3.9 or higher

Required packages: pandas, numpy, openpyxl, matplotlib, difflib (built-in)

2. Input Data Format

| **Dataset** | **File Format** | **Key Columns** | **Description** |
| --- | --- | --- | --- |
| WOS+Scopus merged | Excel (.xlsx) | TI (Title), PY (Year), AU (Authors), DI (DOI) | Main analysis dataset (n = 394) |
| PubMed validation set | CSV (.csv) | Title, Year | Independent PubMed search results (n = 531) |
| PubMed Top 50 cited | CSV (.csv) | Title | 50 most-cited articles from PubMed |

3. Title Normalization Pipeline

All titles were standardized identically across both workflows using the following steps:

Convert to lowercase: title.lower()

Remove punctuation and special characters: re.sub(r'[^\w\s]', '', title)

Collapse multiple whitespaces: re.sub(r'\s+', ' ', title).strip()

4. Overall Database Overlap Analysis (351/531 articles)

Purpose: Assess the representativeness of the WOS+Scopus dataset by measuring its coverage of an independent PubMed validation set.

Matching rule: A PubMed article was classified as "present in WOS+Scopus" if its normalized 50-character prefix was a contiguous substring of any normalized title in the main dataset.

Implementation:

Python:

matches = wos_scopus[wos_scopus['title_clean'].str.contains(

clean[:50], na=False, regex=False

)]

Rationale for substring matching: Scientific article titles are highly distinctive. A 50-character normalized prefix provides sufficient discriminative power for reliable deduplication while avoiding the arbitrariness of similarity thresholds inherent in fuzzy matching algorithms.

Unmatched article classification: PubMed-exclusive articles were categorized into four types based on journal name and publication year:

- Preprint: journal name containing biorxiv, medrxiv, arxiv, or preprint
- Conference: journal name containing conference, proceedings, ieee, embc, or spie
- Recent (2025–2026): publication year ≥ 2025
- Other Journal: all remaining unmatched articles

5. Top 50 Citation Coverage Validation (31/50 articles)

Purpose: Verify whether the WOS+Scopus dataset exhibits systematic bias against high-impact research.

Matching rule: For the 50 most-cited PubMed articles, difflib.SequenceMatcher was used to compute character-level similarity (Ratcliff/Obershelp algorithm) against all WOS+Scopus titles. A match was declared if ratio() >= 0.85.

Implementation:

Python:

best_sim = 0

for _, wos_row in wos_scopus.iterrows():

sim = SequenceMatcher(None, pm_title, wos_row['title_clean']).ratio()

if sim > best_sim:

best_sim = sim

if best_sim >= 0.85:

matched += 1

Threshold rationale: The 0.85 threshold follows industry standards for record linkage and the precedent of Qurashi et al. (2020) for scholarly text deduplication.

6. Threshold Sensitivity Analysis

Three thresholds (0.80, 0.85, 0.90) were tested for the Top 50 fuzzy matching to verify robustness.

| **Threshold** | **Matched** | **Total** | **Coverage (%)** |
| --- | --- | --- | --- |
| **0.80** | **31** | **50** | **62.0** |
| **0.85** | **31** | **50** | **62.0** |
| **0.90** | **31** | **50** | **62.0** |

Coverage remained identical across all tested thresholds, indicating that the 31 matched articles were captured by exact title normalization and that the validation conclusion is completely insensitive to threshold selection within the plausible range (Supplementary Table S9).

7. False-Positive and False-Negative Estimation

- False positives: All 31 matched pairs were manually inspected and verified for title concordance, publication year, and first-author identity. All were confirmed as genuine matches (estimated false-positive rate = 0%).
- False negatives: Ten unmatched articles were randomly sampled and manually searched in the WOS+Scopus dataset using full titles, DOIs, and author-year combinations. None were found, confirming these are genuinely unique to PubMed (estimated false-negative rate ≈ 0%).

8. Code Availability

The complete Python workflow, including title normalization, deduplication, cross-database matching, and visualization scripts, is provided as Supplementary Code (Supplementary_Code.zip). All input data formats, preprocessing parameters, and matching rules are explicitly documented above to ensure full reproducibility.
