# Supplementary material for: The translational chasm in machine learning for triple-negative breast cancer: a quantitative landscape assessment
Source: Front Oncol. 2026 Sep 24;16:1912317. doi: 10.3389/fonc.2026.1912317 (PMC13645570; doi:10.3389/fonc.2026.1912317)
Supplement: Supplementary file 2 [file Table1.docx]

**Supplementary Table S1.Ketwords clusters information.**

| **Cluster id** | **size** | **Sihouette** | **mean(Year)** | **Topterms(LSI)** | **Topterms(log-likelihoodratioP-level)** | **Termsmutualinformations** |
| --- | --- | --- | --- | --- | --- | --- |
| 0 | 73 | 0.675 | 2017 | breast cancer; machine learning; random forest; dna damage repair; integrative analysis \| triple-negative breast cancer; machine learning; digital pathology; breast cancer; tumor-infiltrating lymphocytes | internal medicine (8.18, 0.005); pathological complete response (8.16, 0.005); triple negative (7.42, 0.01); deep learning (7.02, 0.01); neoadjuvant therapy (6.7, 0.01) | whole-slide image (2.85); nanoparticle research (2.85); firefly algorithm (2.85); dna damage repair (2.85); neotrip (2.85) |
| 1 | 50 | 0.857 | 2024 | triple-negative breast cancer; targeted therapies; 2 mutations; precision oncology; pathological complete response \| breast cancer; feature mapping; precision oncology; pathological complete response; targeted therapies | drug delivery (12.32, 0.001); pathologic complete response (8.6, 0.005); biomarkers (6.97, 0.01); precision oncology (6.97, 0.01); tnbc (6.37, 0.05) | mitophagy agonist (0.16); autophagy (0.16); nanomedicine (0.16); aptamer-sirna conjugates (0.16); nanotechnology (0.16) |
| 2 | 46 | 0.864 | 2022 | computational biology; computer science; internal medicine; precision medicine; artificial intelligence \| breast cancer; neoadjuvant chemotherapy; treatment response monitoring; quantitative ultrasound; systems biology | computational biology (18.49, 1.0E-4); precision medicine (10.77, 0.005); cancer research (8.28, 0.005); biology (8.26, 0.005); machine learning (6.63, 0.05) | spectral analysis (0.91); fingerprint (computing) (0.91); phosphorylation (0.91); neuroscience (0.91); breast disease (0.91) |
| 3 | 34 | 0.868 | 2022 | triple-negative breast cancer; machine learning; tumor microenvironment; model-based learning; breast cancer pathogenesis \| breast cancer; tumor resistance; bone metastasis; regulated cell death; experimental models | unsupervised learning (12.48, 0.001); metformin (12.48, 0.001); single-cell rna sequencing (12.48, 0.001); single cell (8.76, 0.005); single-cell rnaseq (6.23, 0.05) | single-cell rnaseq (0.15); artificial intelligence (ai) (0.15); mixture-models (0.15); blood-based biomarkers (0.15); immune response (0.15) |
| 4 | 22 | 0.963 | 2016 | applied mathematics; transcription factor; differential equation; signal transduction; ordinary differential equation \| artificial neural network; internal medicine; breast cancer; machine learning; deep learning | cancer cell (8.95, 0.005); cell biology (7.82, 0.01); transcription factor (7.17, 0.01); actin cytoskeleton (7.17, 0.01); actin (7.17, 0.01) | transcription factor (0.08); actin cytoskeleton (0.08); actin (0.08); cytoskeleton (0.08); ode (0.08) |
| 5 | 22 | 0.799 | 2015 | triple-negative breast cancer; cancer stem cell; tumor microenvironment; virtual screening; artificial intelligence \| breast cancer; egfr inhibitors; cancer research; epidermal growth factor receptor; signal transduction | epidermal growth factor receptor (10.14, 0.005); egfr inhibitors (10.14, 0.005); tertiary lymphoid structures (10.14, 0.005); protein-protein interaction network (6.48, 0.05); afatinib (5.06, 0.05) | afatinib (0.32); controlled drug release (0.32); proteins (0.32); tumor budding (0.32); treatment methods (0.32) |
| 6 | 21 | 0.964 | 2021 | machine learning; drug repurposing; wee1 kinase; wee1 inhibitor; triple-negative breast cancer \| prediction reliability; big data analytics; data mining; anti-cancer targeted therapy; drug discovery | drug repurposing (17.52, 1.0E-4); anti-cancer targeted therapy (8.71, 0.005); diazoxide (8.71, 0.005); data mining (8.71, 0.005); tri-factorization (8.71, 0.005) | anti-cancer targeted therapy (0.03); diazoxide (0.03); data mining (0.03); tri-factorization (0.03); dactolisib (0.03) |
| 7 | 20 | 0.853 | 2021 | magnetic resonance imaging; triple-negative breast cancer; prediction model; machine learning; axillary lymph node metastasis \| breast cancer; automatic segmentation; molecular subtypes; artificial intelligence; internal medicine | magnetic resonance imaging (18.5, 1.0E-4); color doppler ultrasound (6.25, 0.05); disease progression (6.25, 0.05); radiogenomics (6.25, 0.05); molecular subtypes (6.25, 0.05) | color doppler ultrasound (0.15); disease progression (0.15); radiogenomics (0.15); molecular subtypes (0.15); her2-enriched (0.15) |
| 8 | 19 | 0.935 | 2020 | deep learning; feature extraction; transfer learning; image segmentation; computer architecture \| breast cancer; internal medicine; genome instability; ovarian cancer; serous ovarian cancer | tumors (12.71, 0.001); transfer learning (12.71, 0.001); feature extraction (8.97, 0.005); accuracy (6.34, 0.05); genome instability (6.34, 0.05) | accuracy (0.14); genome instability (0.14); computer architecture (0.14); convolutional neural networks (0.14); ovarian cancer (0.14) |
| 9 | 17 | 0.946 | 2021 | triple-negative breast cancer; machine learning; immunogenomic profiling; tumor immunity; deep learning \| deep learning; molecular representation; deep convolutional neural network; phosphatidylinositol-3 kinases alpha isoform; triple-negative breast cancer | molecular dynamics (15.48, 1.0E-4); 3-dioxygenase (tdo) (15.48, 1.0E-4); indoleamine 2 (15.48, 1.0E-4); mm-gbsa (15.48, 1.0E-4); molecular dynamics simulation (15.48, 1.0E-4) | 3-dioxygenase (ido) (0.06); nanobody (0.06); trop2 (0.06); spr (0.06); src (0.06) |
| 10 | 13 | 0.991 | 2016 | metabolic signatures; triple-negative breast cancer; drug sensitivity; immune infiltration; multimodal metabolomics \| multimodal metabolomics; random forest; breast cancer subtypes; attention-based deep learning; self-organizing map | metabolic signatures (17.92, 1.0E-4); svm-rfe (8.91, 0.005); attention-based deep learning (8.91, 0.005); breast cancer subtypes (8.91, 0.005); xgb-rfe (8.91, 0.005) | svm-rfe (0.03); attention-based deep learning (0.03); breast cancer subtypes (0.03); xgb-rfe (0.03); feature selection (0.03) |
| 12 | 6 | 1 | 2026 | breast cancer heterogeneity; ebracteolatain a; machine learning; metabolic reprogramming; raman imaging; therapeutic resistance | raman imaging (11.44, 0.001); breast cancer heterogeneity (11.44, 0.001); ebracteolatain a (11.44, 0.001); therapeutic resistance (11.44, 0.001); metabolic reprogramming (8.68, 0.005) | breast cancer (0.04); triple-negative breast cancer (0.04); machine learning (0.02); cancer (0.02); medicine (0.02) |

**Supplementary Table S2.References clusters information.**

| cluster id | size | Sihouette | mean(Year) | Top terms(LSI) | Top terms(log-likelihood ratio P-level) | Terms mutual informations |
| --- | --- | --- | --- | --- | --- | --- |
| 17 | 4 | 0.993 | 2020 | prediction of pathologic complete response to neoadjuvant systemic therapy in triple negative breast cancer using deep learning on multiparametric mri | neoadjuvant systemic therapy (13.16, 0.001); prediction (13.16, 0.001); using deep learning (13.16, 0.001); multiparametric mri (6.91, 0.01); pathologic complete response (4.75, 0.05) | triple-negative breast cancer (0.11); breast cancer (0.06); triple negative breast cancer (0.01); neoadjuvant chemotherapy (0.01); tertiary lymphoid structure (0.01) |
| 10 | 29 | 0.959 | 2022 | breast cancer; systematic review; radiomics feature; biological dictionary; dictionary version \| deep learning; triple-negative breast cancer; predicting long-term prognosis; clinical outcome; triple-negative breast cancer cases | revolutionizing early detection (58.39, 1.0E-4); next-gen diagnostic strategies (58.39, 1.0E-4); non-invasive biomarker (58.39, 1.0E-4); dictionary version (54.19, 1.0E-4); personalized breast cancer (54.19, 1.0E-4) | longitudinal temporal depth network fusion model (0.58); t cell infiltration (0.58); key prognostic gene (0.58); automated mitotic spindle hotspot count (0.58); different machine (0.58) |
| 9 | 33 | 0.999 | 2015 | triple negative breast cancer; recent advance; triple negative breast cancer treatment; neoadjuvant chemotherapy; triple-negative breast cancer \| triple-negative breast cancer; clinicopathological feature; epigenetic subtype; transcriptomic change; neoadjuvant chemotherapy | recent advance (41.21, 1.0E-4); triple negative breast cancer treatment (41.21, 1.0E-4); epigenetic subtype (20.43, 1.0E-4); triple negative breast cancer (17.26, 1.0E-4); transcriptomic change (10.17, 0.005) | triple-negative breast cancer (0.1); breast cancer (0.04); transcriptomic change (0.02); t cell infiltration (0.01); key prognostic gene (0.01) |
| 7 | 34 | 0.957 | 2017 | triple-negative breast cancer; breast cancer; deep learning; computational tumor-infiltrating lymphocyte assessment method; visual reporting guideline \| accurate segmentation; data mining; mapping tumor heterogeneity; computational tumor-infiltrating lymphocyte assessment method; t cell infiltration | visual reporting guideline (66.91, 1.0E-4); computational tumor-infiltrating lymphocyte assessment method (66.91, 1.0E-4); open-source automated tumor-infiltrating lymphocyte algorithm (61.3, 1.0E-4); data mining (55.68, 1.0E-4); sequencing validation (55.68, 1.0E-4) | t cell infiltration (0.29); fgfr blockade (0.29); regulating cancer-associated fibroblast (0.29); key prognostic gene (0.29); automated mitotic spindle hotspot count (0.29) |
| 4 | 49 | 0.909 | 2021 | triple-negative breast cancer; breast cancer; tertiary lymphoid structure; distinctive characteristics; machine learning \| comprehensive review; th2-associated microenvironment; draining lymph node; cold triple-negative breast cancer; artificial intelligence | tertiary lymphoid structure (93.69, 1.0E-4); single-cell analysis (48.74, 1.0E-4); distinctive characteristics (48.74, 1.0E-4); evolving therapeutics (47.66, 1.0E-4); ensuing cardiotoxicities (47.66, 1.0E-4) | automated mitotic spindle hotspot count (1.7); untreated early-stage triple-negative breast cancer (1.7); t cell infiltration (1.7); key prognostic gene (1.7); different machine (1.7) |
| 3 | 49 | 0.901 | 2019 | breast cancer; molecular subtype; triple-negative breast cancer; neoadjuvant chemotherapy; pathological complete response \| ultrasonographic morphological characteristics; deep learning-based computer-aided diagnostic system; multitask machine; invasive ductal breast carcinoma; preoperative prediction | molecular subtype (102.16, 1.0E-4); pathological complete response (77.99, 1.0E-4); radiomics-based machine learning model (66.77, 1.0E-4); radiomics analysis (63.04, 1.0E-4); nontriple-negative breast cancer (63.04, 1.0E-4) | key prognostic gene (0.88); lasso-based machine learning (0.88); bioinformatics analysis (0.88); t cell infiltration (0.88); automated mitotic spindle hotspot count (0.88) |
| 1 | 50 | 0.915 | 2019 | triple-negative breast cancer; breast cancer; artificial intelligence; working group; tumor-infiltrating lymphocyte \| automatic segmentation; predicting breast cancer subtype; different machine; learning model; clinical validity | artificial intelligence (119.81, 1.0E-4); working group (111.52, 1.0E-4); infiltrating lymphocyte (71.07, 1.0E-4); learning-based assessment (66.6, 1.0E-4); computational pathology approaches (62.13, 1.0E-4) | different machine (0.57); clinical validity (0.57); t cell infiltration (0.57); key prognostic gene (0.57); automated mitotic spindle hotspot count (0.57) |
| 0 | 68 | 0.86 | 2021 | triple-negative breast cancer; breast cancer; triple negative breast cancer; underlying mechanism; emerging strategies \| pathologic complete response; retrospective study; using color doppler ultrasound; predictive model; disease-free progression | emerging strategies (60.17, 1.0E-4); underlying mechanism (60.17, 1.0E-4); pathologic complete response (57.58, 1.0E-4); therapeutic approaches (57.53, 1.0E-4); molecular pathway (57.53, 1.0E-4) | spatial distribution characteristics (1.67); tumor-associated neutrophil (1.67); prognostic significance (1.67); non-metaplastic triple-negative breast cancer (1.67); t cell infiltration (1.67) |

**Supplementary Table S3.Verification of top 50 PubMed-cited articles against the WOS+Scopus merged dataset**

| pubmed_title | match_type | similarity |
| --- | --- | --- |
| Deep-learning and transfer learning identify new breast cancer survival subtypes from single-cell imaging data | Not found | 0.5171102661596958 |
| Multi-omic analysis identifies metabolic biomarkers for the early detection of breast cancer and therapeutic response prediction | Exact | 1 |
| Using biological constraints to improve prediction in precision oncology | Exact | 1 |
| Non-linear Deep Neural Network for Rapid and Accurate Prediction of Phenotypic Responses to Kinase Inhibitors | Exact | 1 |
| Exploring the Role of APC in Modulating Chemotherapeutic Response in Triple-Negative Breast Cancer cells | Not found | 0.6190476190476191 |
| Pathologist-interpretable breast cancer subtyping and stratification from AI-inferred nuclear features | Not found | 0.5348837209302325 |
| Artificial intelligence-enabled automated analysis of transmission electron micrographs to evaluate chemotherapy impact on mitochondrial morphology in triple negative breast cancer | Not found | 0.5742574257425742 |
| Enhanced prediction of breast cancer patient response to chemotherapy by integrating deconvolved expression patterns of immune, stromal and tumor cells | Not found | 0.5142857142857142 |
| Spatially distinct cellular and molecular landscapes define prognosis in triple negative breast cancer | Not found | 0.5738396624472574 |
| Optimizing Neoadjuvant Treatment Response Prediction for Triple-Negative Breast Cancer Using Clinical Trial Data and Deep Auxiliary Learning | Not found | 0.7489361702127659 |
| Virtual patient analysis identifies strategies to improve the performance of predictive biomarkers for PD-1 blockade | Exact | 1 |
| Cellos: High-throughput deconvolution of 3D organoid dynamics at cellular resolution for cancer pharmacology | Not found | 0.44559585492227977 |
| Spatial and Compositional Biomarkers in Tumor Microenvironment Predicts Clinical Outcomes in Triple-Negative Breast Cancer | Not found | 0.7890625 |
| Combined kinome inhibition states are predictive of cancer cell line sensitivity to kinase inhibitor combination therapies | Not found | 0.4576271186440678 |
| Predicting neoadjuvant treatment response in triple-negative breast cancer using machine learning | Exact | 1 |
| A Machine Learning-optimized system for on demand, pulsatile, photo- and chemo-therapeutic treatment using near-infrared responsive MoS (2) -based microparticles in a breast cancer model | Fuzzy (0.96) | 0.9649122807017544 |
| Computational ranking-assisted identification of Plexin-B2 in homotypic and heterotypic clustering of circulating tumor cells in breast cancer metastasis | Not found | 0.5051903114186851 |
| Identification of lactylation-related biomarkers for diagnosis, prognosis, and treatment responsiveness in triple-negative breast cancer | Not found | 0.6075949367088608 |
| AI assessment of tumor-infiltrating lymphocytes on routine H&E-slides as a predictor of response to neoadjuvant therapy in breast cancer-a real-world study | Exact | 1 |
| Automated quantification of stromal tumour infiltrating lymphocytes is associated with prognosis in breast cancer | Exact | 1 |
| Identification and validation of synergistic drug strategies targeting macrophage polarization in triple-negative breast cancer via single-cell transcriptomics and deep learning | Exact | 1 |
| Comprehensive characterization of immune landscape of Indian and Western triple negative breast cancers | Exact | 1 |
| Concordance assessment of Watson for Oncology in breast cancer chemotherapy: first China experience | Not found | 0.4875 |
| Regulation of ferroptosis-related genes in CD8+ NKT cells and classical monocytes may affect the immunotherapy response after combined treatment in triple negative breast cancer | Fuzzy (1.00) | 0.997134670487106 |
| FGFR blockade boosts T cell infiltration into triple-negative breast cancer by regulating cancer-associated fibroblasts | Exact | 1 |
| Virtual multiplex immunofluorescence identifies lymphocyte subsets predictive of response to neoadjuvant therapy | Exact | 1 |
| Random forest algorithm identifies miRNA signatures for breast cancer detection and classification from patient urine samples | Exact | 1 |
| A Machine Learning Approach to Differentiate Two Specific Breast Cancer Subtypes Using Androgen Receptor Pathway Genes | Exact | 1 |
| Investigation of Candidate Genes and Pathways in Basal/TNBC Patients by Integrated Analysis | Not found | 0.48598130841121495 |
| circUSP42 Is Downregulated in Triple-Negative Breast Cancer and Associated With Poor Prognosis | Not found | 0.5570776255707762 |
| Activation of epidermal growth factor receptors in triple-negative breast cancer cells by morphine; analysis through Raman spectroscopy and machine learning | Exact | 1 |
| A Prognostic Model of Genetic Markers for Triple-Negative Breast Cancer Based on Machine Learning and Bioinformatics Analysis | Not found | 0.7874015748031497 |
| Predicting Overall Survival in METABRIC Cohort Using Machine Learning | Not found | 0.6060606060606061 |
| Raman imaging uncovers Ebracteolatain A's subtype-selective metabolic reprogramming in luminal versus triple-negative breast cancer cells | Exact | 1 |
| Serum Raman spectroscopy combined with convolutional neural network for rapid diagnosis of HER2-positive and triple-negative breast cancer | Exact | 1 |
| Morphological signatures of actin  organization in single cells accurately classify genetic perturbations using CNNs with transfer learning | Exact | 1 |
| Freeze-Thaw-Induced Patterning of Extracellular Vesicles with Artificial Intelligence for Breast Cancers Identifications | Exact | 1 |
| (18)F-FDG PET/CT for prediction of response in breast cancer | Not found | 0.6607142857142857 |
| The receptor AXL diversifies EGFR signaling and limits the response to EGFR-targeted inhibitors in triple-negative breast cancer cells | Exact | 1 |
| QAL333's antitumor activity and predictive modeling: integrated transcriptomic-bioinformatic analysis reveals selective cytotoxicity and sensitivity determinants | Not found | 0.4068965517241379 |
| Ensemble model for neoadjuvant chemotherapy response prediction and treatment sensitivity in TNBC based on DNA replication stress signatures | Exact | 1 |
| Efferocytosis-related signatures identified via Single-cell analysis and machine learning predict TNBC outcomes and immunotherapy response | Exact | 1 |
| BoxCell: Leveraging SAM for Cell Segmentation with Box Supervision | Exact | 1 |
| Machine learning identifies SRD5A3 as a propionate-related prognostic biomarker in triple-negative breast cancer | Exact | 1 |
| Cohesive data analysis for the identification of prognostic hub genes and significant pathways associated with HER2 + and TN breast cancer types | Fuzzy (0.99) | 0.9929577464788732 |
| Prediction of axillary lymph node metastasis in triple negative breast cancer using MRI radiomics and clinical features | Exact | 1 |
| Uncovering subtype-specific metabolic signatures in breast cancer through multimodal integration, attention-based deep learning, and self-organizing maps | Exact | 1 |
| A whole slide image-based risk score predicts prognosis and postmastectomy radiotherapy benefit in triple negative breast cancer patients | Exact | 1 |
| Deep learning for survival prediction in triple-negative breast cancer: development and validation in real-world cohorts | Exact | 1 |
| Immune microenvironment and prognostic genes of triple-negative breast cancer in the context of transcriptome and single-cell sequencing | Exact | 1 |

**Supplementary Table S4. Top 15 raw high-frequency keywords in WoS+Scopus versus PubMed**

| Rank | WoS+Scopus(n=394) | Frequency | PubMed(n=531) | Frequency |
| --- | --- | --- | --- | --- |
| 1 | tnbc | 1137 | tnbc | 1503 |
| 2 | breast cancer | 596 | breast cancer | 777 |
| 3 | triple-negative breast cancer | 569 | triple-negative breast cancer | 733 |
| 4 | model | 564 | model | 672 |
| 5 | tumor | 360 | tumor | 505 |
| 6 | features | 348 | clinical | 464 |
| 7 | treatment | 341 | treatment | 452 |
| 8 | models | 339 | genes | 441 |
| 9 | clinical | 331 | models | 413 |
| 10 | machine learning | 299 | features | 402 |
| 11 | cancer | 293 | cells | 401 |
| 12 | cell | 285 | cell | 399 |
| 13 | genes | 281 | cancer | 391 |
| 14 | cells | 270 | response | 378 |
| 15 | based | 270 | machine learning | 356 |

Note: Keywords were extracted from titles and abstracts with compound phrase preservation (e.g., "machine learning" treated as a single unit), stop word filtering, and no synonym merging. Jaccard similarity coefficient = 0.875; Spearman rank correlation coefficient ρ = 0.824, P = 0.0003.

**Supplementary Table S5. Summary of clinical validation studies of machine learning in triple-negative breast cancer indexed in PubMed (2011–2026)**

| **No.** | **First Author** | **Year** | **Journal** | **Sample Size** | **ML Application Scenario** | **Main Findings / Performance Indicators** | **Multi-center / Study Type** |
| --- | --- | --- | --- | --- | --- | --- | --- |
| 1 | Li X | 2024 | J Pathol | NSCLC+TNBC cohort (large sample, exact n not reported) | Immunotherapy benefit prediction (PD-L1) | Automated immunophenotyping predicted anti-PD-L1 treatment efficacy; combined method outperformed single approach | Multi-center (retrospective validation) |
| 2 | Terkelsen | 2021 | Mol Oncol | 35 TIF cases (Luminal 19, Her2 4, TNBC 12) | Proteomics-based biomarker discovery | 24-protein biomarker panel; 10 proteins distinguished subtypes; sensitivity / specificity | Multi-center (cohort study) |
| 3 | Ma M | 2022 | Eur Radiol | 600 cases (training 450, test 150) | Molecular subtype diagnosis (TNBC vs. others) | Decision tree AUC = 0.971; accuracy 0.947, sensitivity 0.905, specificity 0.914 | Multi-center (retrospective validation) |
| 4 | Roy S | 2022 | Eur J Nucl | PDX model + clinical validation (exact n not specified) | NAC efficacy prediction (FDG-PET radiomics) | NB-RadSig and SVM-RadSig prediction/assessment of treatment response outperformed S | Single-center (clinical validation, preclinical + clinical) |
| 5 | Yin HL | 2023 | J Cancer | 319 BI-RADS 4 lesions | TNBC vs. fibroadenoma differential diagnosis (multiparametric MRI deep learning) | AI combined score AUC = 0.944; AI assistance improved junior radiologist AUC | Single-center (retrospective validation) |
| 6 | Ma M | 2022 | Eur J Radiol | 147 TNBC NAC patients (training 104, test 43) | Post-NAC systemic relapse prognosis prediction (MRI radiomics) | Model 3 (pre + post-NAC) AUC = 0.963 (training) / 0.935 (test) | Single-center (retrospective validation) |
| 7 | Dihge L | 2019 | Clin Cancer Res | 3,023 cases (training 2,278, validation 745) | Lymph node metastasis prediction (gene expression + clinicopathology) | MIXED model AUC = 0.72; CLINICAL AUC = 0.65–0.73; GE | Multi-center (population-based cohort study) |
| 8 | Huang R | 2026 | Front Oncol | 19,289 | Prognosis prediction | C-index=0.7494; AUC=0.889/0.773/0.740 | Single-center (retrospective validation) |
| 9 | Liang H | 2026 | Front Immunol | 32 | Digital pathology; Immune; NAC response | AUC=0.81 | Multi-center(Retrospective) |
| 10 | Lu D | 2026 | Front Oncol | 248 | NAC response prediction | AUC=0.71 | Single-center(Retrospective) |
| 11 | Ruan M | 2026 | Front Oncol | 379 | Imaging diagnosis; Molecular subtyping | AUC=0.891/0.863/0.818 | Multi-center (Retrospective) |
| 12 | Zilalan Y | 2026 | Front Oncol | 668 | Imaging; Prognosis | AUC=0.946/0.898 | Single-center (Retrospective) |
| 13 | Zhang XM | 2025 | EClinicalMedicine | 2,045/2,793 | Imaging; Prognosis; Digital pathology; Molecular subtyping | AUC=0.980/0.860-0.936 | Multi-center (Retrospective) |
| 14 | Fu M | 2026 | Comput Methods Programs Biomed | 359 | Imaging; NAC response | AUC=0.992/0.877/0.768 | Multi-center (Retrospective) |
| 15 | Alzate-Granados JP | 2025 | Clin Breast Cancer | 4,808 | Prognosis prediction | AUC=0.78/0.76 | Not specified (Retrospective) |
| 16 | Xu Y | 2025 | Sci Rep | 37,818 | Prognosis prediction | C-index=0.824/0.816/0.758 | Single-center (Retrospective) |
| 17 | Li F | 2025 | Front Immunol | Not specified | Prognosis; Immune | Not specified | Single-center (Retrospective transcriptomic) |
| 18 | Wang C | 2025 | Front Immunol | 23,729 | Prognosis prediction | C-index=0.854; AUC=0.901/0.834 | Single-center (Retrospective) |
| 19 | Vidal JM | 2024 | EClinicalMedicine | 294 | Immune; Digital pathology; Prognosis | Spearman r=0.63-0.73 | Multi-center (Retrospective + prospective cohort) |
| 20 | Kim SY | 2024 | Sci Rep | 852 | Imaging; NAC response | AUC=0.74 | Single-center (Retrospective) |
| 21 | Sun K | 2023 | Front Oncol | 91 | Imaging; Molecular subtyping | AUC=0.85 | Not specified (Retrospective) |
| 22 | Zhou Z | 2023 | Sci Rep | 210 | Imaging; NAC response | AUC=0.97/0.82/0.86/0.83 | Multi-center (Retrospective + prospective) |
| 23 | Gou Q | 2022 | Front Pharmacol | 491 | Prognosis; Immune; Treatment response | Not specified | Multi-center (Multi-cohort bioinformatics) |
| 24 | Bai Y | 2021 | Clin Cancer Res | 920 | Immune; Digital pathology; Prognosis | HR=0.30-0.35 | Multi-center (Retrospective validation) |

**Supplementary Table S6.Details of registered trials identified through ClinicalTrials.gov and WHO ICTRP for machine learning applications in triple-negative breast cancer.**

| **No.** | **Registry** | **Trial_ID** | **Title** | **Study_Type** | **Status** | **ML_Application** |
| --- | --- | --- | --- | --- | --- | --- |
| 1 | ClinicalTrials.gov | NCT04874064 | TRIple Negative Breast Cancer Markers In Liquid Biopsies Using Artificial Intelligence | Observational | Recrutting | Imaging AI; Genomic/Omics AI; Liquid biopsy AI; Multimodal AI |
| 2 | ClinicalTrials.gov | NCT05435352 | Image-based AI Predictive Biomarkers for Precision Neoadjuvant Triple-negative Breast Cancer Treatment | Observational | Twrminated | Imaging AI; Genomic/Omics AI; Liquid biopsy AI; Treatment response AI |
| 3 | ClinicalTrials.gov | NCT04461990 | Clinical Study of Imaging Genomics Based on Machine Learning for BCIG | Observational | Unknown | Imaging AI; Genomic/Omics AI; Liquid biopsy AI |
| 4 | ClinicalTrials.gov | NCT05916755 | Predictive Biomarkers of Response to Checkpoint Inhibitors in Triple Negative Breast Cancer: a Multiomics Platform | Observational | Recrutting | Imaging AI; Genomic/Omics AI; Liquid biopsy AI; Multimodal AI; Treatment response AI |
| 5 | ClinicalTrials.gov | NCT06441240 | Evaluation of Recurrence Risk Factors in Locally Advanced Breast Cancer Patients Underwent Neoadjuvant Chemotherapy. | Observational | Unknown | Imaging AI; Digital pathology AI; Genomic/Omics AI; Liquid biopsy AI; Prognostic AI; Treatment response AI |
| 6 | WHO ICTRP (ChiCTR) | ChiCTR2600122646 | Clinical validation of artificial intelligence model based on DCE-MRI for predicting the efficacy of neoadjuvant chemotherapy combined with immunotherapy in locally advanced triple negative breast cancer: a multicenter, observational, prospective study | Observational | Pending | Imaging AI; Treatment response AI |
| 7 | WHO ICTRP (ChiCTR) | ChiCTR2600118205 | Research on the Combined Prediction of Molecular Typing of Breast Cancer by Ultrasound Artificial Intelligence | Diagnostic test | Recruiting | Imaging AI; Molecular subtyping |
| 8 | WHO ICTRP (ChiCTR) | ChiCTR2500111225 | A prospective observational study on digital pathology and artificial intelligence-based prediction of treatment response to neoadjuvant therapy in breast cancer patients | Observational study | Pending | Digital pathology AI; Treatment response AI |
| 9 | WHO ICTRP (ChiCTR) | ChiCTR2400092187 | To study the application value of machine learning mammography and serum Raman spectroscopy in the prediction of triple negative breast cancer and histological grade | Observational study | Pending | Imaging AI; Liquid biopsy AI |

**Supplementary Table S7. Excluded articles from PubMed Tier 2 expanded retrieval with reasons for exclusion.**

| **No.** | **PMID** | **First_Author** | **Year** | **Journal** | **Title** | **Exclude_Reason** |
| --- | --- | --- | --- | --- | --- | --- |
| 1 | 42453872 | Peng X | 2026 | Front Oncol | Artificial intelligence for triple-negative breast cancer from imaging to multi-omics | Review/Editorial/Comment |
| 2 | 42404642 | Zhang X | 2026 | J Clin Imaging Sci | Construction of an ultrasound feature-based diagnostic model for predicting triple-negative breast cancer using 108 machine learning algorithm combinations | Review/Editorial/Comment |
| 3 | 42147216 | Wang Z | 2026 | Front Oncol | AI-mediated ultrasound radiomics in the diagnosis and treatment of triple-negative breast cancer: research progress and future challenges | Review/Editorial/Comment |
| 4 | 41588712 | Li B | 2026 | J Pathol | Hierarchical image pyramid transformer framework for automated breast cancer molecular subtyping using tissue microarrays | Not TNBC-related |
| 5 | 41502591 | Wang J | 2025 | Gland Surg | Machine learning-based prognostic model for metastatic breast cancer and its interpretability: a multicenter retrospective study | Not TNBC-related |
| 6 | 41491736 | Hosseinzadeh N | 2026 | J Imaging Inform Med | Non-Invasive Breast Cancer Receptor Typing from Mammograms Using Artificial Intelligence: A Systematic Review and Meta-Analysis | Not TNBC-related + Review/Editorial/Comment |
| 7 | 41301040 | Piotrzkowska-Wróblewska H | 2025 | Cancers (Basel) | The Role of Quantitative Ultrasound in Monitoring Neoadjuvant Chemotherapy in Breast Cancer: A Narrative Review | Not TNBC-related + Review/Editorial/Comment |
| 8 | 41142553 | Chen Q | 2025 | Gland Surg | Classification of breast cancer molecular subtypes based on contrast-enhanced ultrasound and superb microvascular imaging using machine learning approach | Not TNBC-related |
| 9 | 41094242 | Zhang J | 2025 | J Appl Clin Med Phys | MRI-based radiomics models for early predicting pathological response to neoadjuvant chemotherapy in triple-negative breast cancer: A systematic review and meta-analysis | Review/Editorial/Comment |
| 10 | 41048341 | Muthamilselvan S | 2025 | Front Bioinform | BC-predict: mining of signal biomarkers and production of models for early-stage breast cancer subtyping and prognosis | Not TNBC-related |
| 11 | 40959564 | Wen Y | 2025 | Int J Med Sci | Dual-Function RNA Biomarkers: Integrating Relapse Prediction and Immune Profiling in Triple-Negative Breast Cancer | Review/Editorial/Comment |
| 12 | 40704490 | De Placido P | 2025 | Curr Opin Oncol | Circulating tumor DNA in early-stage triple-negative breast cancer: clinical landscape and key open challenges | Review/Editorial/Comment |
| 13 | 40256728 | Da Z | 2025 | PeerJ | Multiple instance learning-based prediction of programmed death-ligand 1 (PD-L1) expression from hematoxylin and eosin (H&E)-stained histopathological images in breast cancer | Not TNBC-related |
| 14 | 40016785 | Feng X | 2025 | Breast Cancer Res | Predicting the efficacy of neoadjuvant chemotherapy in breast cancer patients based on ultrasound longitudinal temporal depth network fusion model | Not TNBC-related |
| 15 | 39966783 | Wenwen | 2025 | BMC Cancer | Integrating ultrasound radiomics and clinicopathological features for machine learning-based survival prediction in patients with nonmetastatic triple-negative breast cancer | Review/Editorial/Comment |
| 16 | 39915183 | Wang Y | 2025 | Acad Radiol | Enhancing Pathological Complete Response Prediction in Breast Cancer: The Added Value of Pretherapeutic Contrast-Enhanced Cone Beam Breast CT Semantic Features | Not TNBC-related |
| 17 | 39720416 | Garcia V | 2024 | J Pathol Inform | Prioritizing cases from a multi-institutional cohort for a dataset of pathologist annotations | Not TNBC-related |
| 18 | 38664594 | Fronk AD | 2024 | Mol Syst Biol | Development and validation of AI/ML derived splice-switching oligonucleotides | Not TNBC-related |
| 19 | 38388367 | Soliman A | 2024 | Diagn Pathol | Artificial intelligence's impact on breast cancer pathology: a literature review | Not TNBC-related + Review/Editorial/Comment |
| 20 | 38291227 | Koh J | 2024 | Sci Rep | Identification of VWA5A as a novel biomarker for inhibiting metastasis in breast cancer by machine-learning based protein prioritization | Not TNBC-related |
| 21 | 37745392 | Yadav S | 2023 | medRxiv | Deep-learning and transfer learning identify new breast cancer survival subtypes from single-cell imaging data | Not TNBC-related + Review/Editorial/Comment |
| 22 | 37647361 | Zhang W | 2024 | Environ Toxicol | Single-cell combined with bulk-RNA data reveal a pattern related to angiogenesis in breast cancer patients: Individualized medicine | Not TNBC-related |
| 23 | 37443117 | Ding Y | 2023 | NPJ Breast Cancer | Multi-center study on predicting breast cancer lymph node status from core needle biopsy specimens using multi-modal and multi-instance deep learning | Not TNBC-related |
| 24 | 37226243 | Tang X | 2023 | Breast Cancer Res | Integration of multiomics data shows down regulation of mismatch repair and tubulin pathways in triple-negative chemotherapy-resistant breast tumors | Not TNBC-related + Not ML-related |
| 25 | 37138891 | Cook D | 2023 | Front Artif Intell | Next generation immuno-oncology tumor profiling using a rapid, non-invasive, computational biophysics biomarker in early-stage breast cancer | Not TNBC-related |
| 26 | 37007742 | Huang Y | 2023 | EClinicalMedicine | Longitudinal MRI-based fusion novel model predicts pathological complete response in breast cancer treated with neoadjuvant chemotherapy: a multicenter, retrospective study | Not TNBC-related |
| 27 | 36911927 | Ferre R | 2023 | Breast Dis | Machine learning analysis of breast ultrasound to classify triple negative and HER2+ breast cancer subtypes | Not TNBC-related + Review/Editorial/Comment |
| 28 | 36852282 | Omar M | 2023 | iScience | Using biological constraints to improve prediction in precision oncology | Not TNBC-related |
| 29 | 34944898 | Lo Gullo R | 2021 | Cancers (Basel) | Assessing PD-L1 Expression Status Using Radiomic Features from Contrast-Enhanced Breast MRI in Breast Cancer Patients: Initial Results | Not TNBC-related + Review/Editorial/Comment |
| 30 | 34900525 | Xu Z | 2021 | Acta Pharm Sin B | Applying artificial intelligence for cancer immunotherapy | Not TNBC-related + Review/Editorial/Comment |
| 31 | 34253738 | Zhao N | 2021 | Nat Commun | Morphological screening of mesenchymal mammary tumor organoids to identify drugs that reverse epithelial-mesenchymal transition | Not TNBC-related |
| 32 | 33753452 | Li CI | 2021 | Clin Cancer Res | Cancer Cell Intrinsic and Immunologic Phenotypes Determine Clinical Outcomes in Basal-like Breast Cancer | Not TNBC-related + Not ML-related |
| 33 | 33536349 | Qin Y | 2021 | Aging (Albany NY) | Tumor microenvironment characterization in triple-negative breast cancer identifies prognostic gene signature | Not ML-related |
| 34 | 31974598 | Zhong G | 2020 | Mol Med Rep | Identification of key genes as potential biomarkers for triple?negative breast cancer using integrating genomics analysis | Not TNBC-related + Not ML-related |
| 35 | 31883894 | Naorem LD | 2020 | Int J Biol Macromol | Comprehensive analysis of dysregulated lncRNAs and their competing endogenous RNA network in triple-negative breast cancer | Review/Editorial/Comment |
| 36 | 31222166 | Balkenhol MCA | 2019 | Lab Invest | Deep learning assisted mitotic counting for breast cancer | Not TNBC-related |
| 37 | 30488443 | Naorem LD | 2019 | J Cell Physiol | Identification of dysregulated miRNAs in triple negative breast cancer: A meta-analysis approach | Not ML-related + Review/Editorial/Comment |
| 38 | 29993812 | Wang A | 2018 | IEEE/ACM Trans Comput Biol Bioinform | ANTENNA, a Multi-Rank, Multi-Layered Recommender System for Inferring Reliable Drug-Gene-Disease Associations: Repurposing Diazoxide as a Targeted Anti-Cancer Therapy | Not TNBC-related |
| 39 | 28627677 | Zhu H | 2017 | Mol Med Rep | Integrated analysis of the potential roles of miRNA?mRNA networks in triple negative breast cancer | Not ML-related |
| 40 | 26756416 | Sutton EJ | 2016 | J Magn Reson Imaging | Breast cancer molecular subtype classifier that incorporates MRI features | Not TNBC-related + Review/Editorial/Comment |
| 41 | 41588757 | Sharma A | 2026 | Curr Pharm Biotechnol | Triple-Negative Breast Cancer and Artificial Intelligence: Current Paradigms in Diagnosis, Therapy Prediction, Prognosis, and Challenges | Review article |
| 42 | 41181480 | Cherukuri AMK | 2025 | Ann Med Surg (Lond) | Precision oncology: AI's breakthrough in TNBC chemoresistance prediction | Letter to the Editor |
| 43 | 40545963 | Hyon JY | 2025 | J Extracell Vesicles | Extracellular Vesicle Proteome Analysis Improves Diagnosis of Recurrence in Triple-Negative Breast Cancer | No ML method |
| 44 | 39227622 | Di Cosimo S | 2024 | Sci Rep | Low-pass whole genome sequencing of circulating tumor cells to evaluate chromosomal instability in triple-negative breast cancer | No ML method |
| 45 | 38083160 | Zhou Z | 2023 | Annu Int Conf IEEE Eng Med Biol Soc | Predicting pathological complete response to neoadjuvant systemic therapy for triple-negative breast cancers using deep learning on multiparametric MRIs | Conference paper |
| 46 | 28918577 | Aravind Kumar M | 2018 | Mol Cell Biochem | Microarray-based SNP genotyping to identify genetic risk factors of triple-negative breast cancer (TNBC) in South Indian population | No ML method |

**Supplementary Table S8. LOESS span sensitivity diagnostics**

| **Span** | **Equivalent Parameters (n×span)** | **Smoothing Window (years)** | **Residual SD** | **R²** | **Predicted 2025** |
| --- | --- | --- | --- | --- | --- |
| 0.30 | 4.5 | ~2.3 | 5.88×10⁻¹⁴ | 1.000 | 121.0 |
| 0.35 | 5.25 | ~2.6 | 4.84 | 0.999 | 119.8 |
| 0.40 | 6.0 | ~3.0 | 12.38 | 0.991 | 118.5 |
| 0.45 | 6.75 | ~3.4 | 12.38 | 0.991 | 118.5 |
| 0.50 | 7.5 | ~3.8 | 14.68 | 0.987 | 116.2 |

Note: The optimal span = 0.4 achieves the best bias-variance balance. Span < 0.4 leads to overfitting (R² → 1.0, ResidualSD → 0); span > 0.4 causes过度平滑 (R² decreases, trajectory flattened).

**Supplementary Table S9. Summary of institutional origins and classification of machine learning studies in triple-negative breast cancer (2011–2026).**

| **Broad Category** | **2011–2015** | **2016–2020** | **2021–2026** | **Total** |
| --- | --- | --- | --- | --- |
| Clinical/Medical Affiliated | 3 (1.3) | 23 (9.9) | 207 (88.8) | 233 (59.3) |
| Academic/Research | 3 (2.6) | 15 (12.8) | 99 (84.6) | 117 (29.8) |
| Engineering/Technical | 1 (2.3) | 4 (9.3) | 38 (88.4) | 43 (10.9) |
| Other/Industry | 0 (0.0) | 0 (0.0) | 1 (100.0) | 1 (0.3) |
| Total | 7 | 42 | 345 | 394 |

Note:Values are n (%). Percentages in rows indicate the proportion of each period within the broad category; percentages in the Total column indicate the proportion of each broad category among all 394 studies.
